# Haloperidol for the treatment of delirium in critically ill patients: an updated systematic review with meta-analysis and trial sequential analysis

**DOI:** 10.1186/s13054-023-04621-4

**Published:** 2023-08-26

**Authors:** Nina Christine Andersen-Ranberg, Marija Barbateskovic, Anders Perner, Marie Oxenbøll Collet, Lone Musaeus Poulsen, Mathieu van der Jagt, Lisa Smit, Jørn Wetterslev, Ole Mathiesen, Mathias Maagaard

**Affiliations:** 1 Department of Anaesthesiology and Intensive Care, Zealand University Hospital, Lykkebækvej 1, 4600 Køge, Denmark; 2 Collaboration for Research in Intensive Care (CRIC), Copenhagen University Hospital - Rigshospitalet, Copenhagen, Denmark; 3 Copenhagen Trial Unit, Centre for Clinical Intervention Research, Copenhagen, Denmark; 4 Department of Intensive Care, Copenhagen University Hospital - Rigshospitalet, Copenhagen, Denmark; 5 Department of Intensive Care, Erasmus MC – University Medical Center, PO Box 2040, 3000 CA Rotterdam, The Netherlands; 6 Private Office, Tuborg Sundpark 3, 1. Th., 2900 Hellerup, Copenhagen, Denmark; 7 Department of Clinical Medicine, Copenhagen University, Copenhagen, Denmark

**Keywords:** Delirium, Haloperidol, Antipsychotics, Systematic review, Meta-analysis

## Abstract

**Background:**

Haloperidol is frequently used in critically ill patients with delirium, but evidence for its effects has been sparse and inconclusive. By including recent trials, we updated a systematic review assessing effects of haloperidol on mortality and serious adverse events in critically ill patients with delirium.

**Methods:**

This is an updated systematic review with meta-analysis and trial sequential analysis of randomised clinical trials investigating haloperidol versus placebo or any comparator in critically ill patients with delirium. We adhered to the Cochrane handbook, the PRISMA guidelines and the grading of recommendations assessment, development and evaluation statements. The primary outcomes were all-cause mortality and proportion of patients with one or more serious adverse events or reactions (SAEs/SARs). Secondary outcomes were days alive without delirium or coma, delirium severity, cognitive function and health-related quality of life.

**Results:**

We included 11 RCTs with 15 comparisons (*n* = 2200); five were placebo-controlled. The relative risk for mortality with haloperidol versus placebo was 0.89; 96.7% CI 0.77 to 1.03; *I*^2^ = 0% (moderate-certainty evidence) and for proportion of patients experiencing SAEs/SARs 0.94; 96.7% CI 0.81 to 1.10; *I*^2^ = 18% (low-certainty evidence). We found no difference in days alive without delirium or coma (moderate-certainty evidence). We found sparse data for other secondary outcomes and other comparators than placebo.

**Conclusions:**

Haloperidol may reduce mortality and likely result in little to no change in the occurrence of SAEs/SARs compared with placebo in critically ill patients with delirium. However, the results were not statistically significant and more trial data are needed to provide higher certainty for the effects of haloperidol in these patients.

*Trial registration*: CRD42017081133, date of registration 28 November 2017.

**Supplementary Information:**

The online version contains supplementary material available at 10.1186/s13054-023-04621-4.

## Background

Delirium is a common, acute and fluctuating disturbance of consciousness, attention and cognition [1]. Critically ill patients are particularly vulnerable, with estimates suggesting that 30–50% of patients in the intensive care unit (ICU) may experience delirium [2, 3]. Delirium is a serious condition with deleterious effects on patient-important outcomes. Studies have associated delirium with increased duration of mechanical ventilation, hospital and ICU stay, increased cognitive impairment and disability 1-year after hospital discharge [3–5]. Delirium has also been associated with increased mortality with longer episodes of delirium translating into higher mortality risk [6].

Currently, no evidence-based pharmacological treatment exists for delirium, and guidelines do not support the routine use of any pharmacological agent for its prevention or treatment [7]. However, in clinical settings, patients with delirium are often treated with various agents, including antipsychotics, alpha-2 agonists, benzodiazepines, opioids, sedatives and others [8]. Among these agents, haloperidol, a typical antipsychotic compound, is the most frequently used agent to treat delirium in the ICU [9]. A recent systematic review and meta-analysis highlighted that the evidence for the use of haloperidol in critically ill patients with delirium was sparse and inconclusive [10]. Since then, new randomised clinical trials (RCTs) have been published, necessitating an updated systematic review, summarising the available evidence on the effects of haloperidol in critically ill patients with delirium.

The aim of this study was to assess patient-important benefits and harms of haloperidol versus placebo or any comparator in critically ill patients with delirium. The primary comparison was haloperidol versus placebo.

## Methods

This updated systematic review was conducted in accordance with a pre-specified and published protocol [11]. The protocol was registered in the international prospective register of systematic reviews (PROSPERO) (CRD42017081133) and the conduct of the review followed the recommendations of the Cochrane Handbook for Systematic Reviews [12], the Preferred Reporting Items for Systematic Reviews and Meta-Analyses (PRISMA) [13] and the Grading of Recommendations Assessment, Development and Evaluation (GRADE) [14].

### Types of trials

We included RCTs, irrespective of publication status, date, language and reported outcomes. We excluded quasi-randomised trials, crossover trials and observational studies.

### Types of participants

We included RCTs randomising critically ill adults with delirium. Delirium, as defined by the trialists, had to be present at the time of randomisation in all participants for a trial to be considered for this review. Critical illness was defined as patients who were at high risk of dying or who had actual or potential life-threatening health problems and who were admitted to a high-dependency facility in the hospital.

### Types of interventions

We included any trial comparing haloperidol with placebo, any other pharmacological agents or combinations of pharmacological and non-pharmacological interventions. The intervention group was defined as those who received haloperidol.

### Outcomes

We assessed two primary outcomes: (1) all-cause mortality and (2) the proportion of patients with one or more serious adverse events or reactions (SAEs/SARs). We defined SAEs and SARs according to the International Conference on Harmonisation Good Clinical Practice (ICH-GCP) and as reported in each trial. Consequently, an SAE was defined as any reported adverse event that resulted in death, was life-threatening, required hospitalisation or prolongation of existing hospitalisation, resulted in persistent or significant disability or incapacity. A SAR was defined as any reported serious adverse event related to haloperidol (according to the Summary of Product Characteristics of haloperidol [15]) that resulted in death, was life-threatening, required hospitalisation or prolongation of existing hospitalisation, resulted in persistent or significant disability or incapacity. Two methods were used to analyse SAEs/SARs: (1) highest proportion of reported SAEs/SARs which was the most frequently reported SAE/SAR in each group and (2) calculating the cumulative number of SAEs/SARs which is the sum of all reported SAEs/SARs in each group. We expected the actual number of patients experiencing one or more SAEs/SARs to fall between these two measures.

The secondary outcomes were (1) days alive without delirium or coma, (2) delirium severity, (3) cognitive function and (4) health-related quality of life. We also assessed QTc prolongation as an exploratory outcome. We assessed all outcomes at maximum follow-up, except for days alive without delirium or coma which was assessed at 14 days after randomisation.

### Search methods

We systematically searched the Cochrane Central Register of Controlled Trials (CENTRAL), MEDLINE, Embase, Science Citation Index, Biosis Previews, Cumulative Index to Nursing & Allied Health Literature (CINAHL) and Latin American Caribbean Health Science Literature (LILACS) from inception to 18 April 2023. Additionally, we screened for ongoing and unpublished trials in trial registries and manually searched reference lists of previous systematic reviews. The full search strategy is available in Additional file 1.

### Trial selection and data extraction

Two authors (NCAR and MM) independently screened titles and abstracts, assessed full-text reports for inclusion and extracted data using pre-defined data extraction forms. We extracted all available data on trial characteristics, characteristics of trial participants, interventions and outcomes.

### Risk of bias assessment

Two authors (NCAR and MM) independently assessed the risk of bias of each reported outcome in the included trials. The risk of bias of outcomes from one trial was assessed by MM and MvdJ as NCAR was the first author of this trial [16]. Risk of bias for each reported outcome was assessed with the Cochrane Risk of Bias 2 Tool (RoB 2) [17]. The overall risk of bias for an outcome was judged as low if all domains were judged to be at low risk of bias and judged as high if one or more domains were either at some concern or at high risk of bias.

We planned to assess publication bias by inspection of funnel plots [12] for signs of asymmetry when 10 or more trials were included in an analysis and planned to test for asymmetry with the Harbord or Thompson test dependent for dichotomous outcomes and Egger test for continuous outcomes [12, 18].

### Measures of treatment effect

For dichotomous outcomes, we reported risk ratios (RRs), and continuous outcomes were reported as mean difference (MD) or standardised mean difference (SMD) if different scales were used. We used a family-wise error rate of 5% and as we have two primary outcomes a *P* value of 0.05/((2 + 1)/2) = 0.033 or less was considered statistically significant (corresponding to 96.7% CI) and correspondingly for the four secondary outcomes a *P* value < 0.02 (corresponding to a 98% CI) was considered statistically significant [19]. We also calculated trial sequential analysis (TSA)-adjusted CIs accounting for the uncertainty due to sparse data and multiple outcomes.

### Dealing with missing data

Corresponding authors were contacted at least twice if data were missing or unclear (Additional file 1; details of included trials). Medians and interquartile ranges were converted to means and standard deviation by methods described by Lou et al. [20] and Wang et al. [21]. To assess the impact of patients lost to follow-up, we conducted pre-planned sensitivity analyses with best-/worst-case and worst-/best-case scenarios (Additional file 1).

### Meta-analysis

We calculated pooled effect estimates using the statistical software R, version 4.2.0 (R, Core Team, R Foundation for Statistical Computing, Vienna, Austria) using the *Meta* and *Tidyverse* packages. We assessed the intervention effect with both a random-effects model and a fixed-effects model and reported the most conservative estimate (closest to no effect) with the widest CI [11, 19].

All meta-analyses were subgrouped according to control intervention (e.g. placebo, other antipsychotics, benzodiazepines, etc.).

### Assessment of heterogeneity

We assessed heterogeneity primarily by visual inspection of forest plots. We also calculated inconsistency (*I*^2^) and diversity (*D*^2^) statistics. Clinical heterogeneity was explored by conducting pre-specified subgroup analyses.

### Sensitivity and subgroup analyses

We planned to conduct the following pre-defined subgroup analyses: trials at overall high risk of bias compared with trials at overall low risk of bias, grouping according to patient population and delirium motor subtype.

### Assessment of risk of random errors

We assessed the risk of random errors of each outcome with TSA. We used a power of 90% (beta 10%) and a diversity (D^2^) as suggested by the trials in the meta-analysis or a diversity of 20% if the actual measured diversity was zero as diversity will most likely increase when further trials are added until the required information size is reached [22]. As anticipated intervention effects, we used a priori relative risk reduction (RRR) or relative risk increase (RRI) of 20%. For continuous outcomes, this was calculated as a 20% RRR/RRI of the weighted mean in the control groups. A post hoc TSA sensitivity analysis was conducted where we used the pooled effect estimate and diversity from the meta-analysis of each outcome (alpha level 3.3% or 2%, power 90%).

### Assessment of the overall certainty of evidence

We evaluated the certainty of evidence for each outcome using the Grading of Recommendations Assessment, Development and Evaluation (GRADE) approach [23]. The overall certainty of evidence was rated as either high, moderate, low or very low based on our evaluation of the identified risk of bias, inconsistency, indirectness, imprecision and publication bias.

Our conclusions followed the GRADE guideline 26 [24], which provides recommendations on how to communicate results of systematic reviews with informative statements rather than merely describe results as statistically or not statistically significant and avoid the common misinterpretation that large P values mean ‘no difference’ or ‘no effect’. Instead, review authors are encouraged to focus on the point estimate and the certainty of that estimate which considers multiple factors (GRADE assessment) [24].

### Protocol deviations

We used RoB 2 tool to assess risk of bias of each reported outcome. In accordance with the original review, we used a power of 90% in the TSA and not 80% as pre-defined in the review protocol [11], as meta-analysis should use higher (or same) power as its included trials, to be able to communicate the best available evidence. In addition, we used a diversity of 20% if the measured diversity was zero.

## Results

**Fig. 1:**
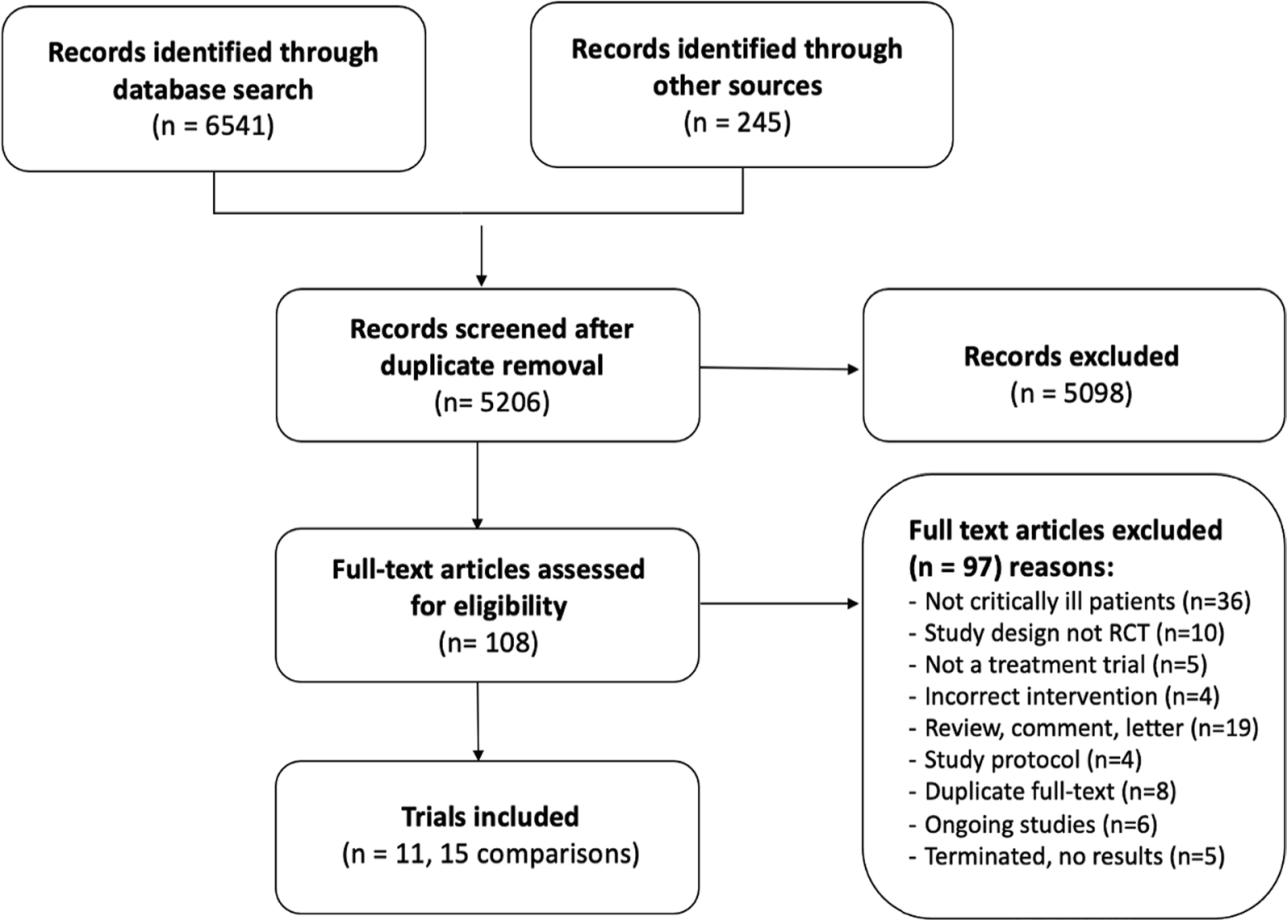
PRISMA flow diagram

We screened 6541 records, assessed 104 trials in full text and included 11 trials with 15 reported comparisons and a total of 2200 randomised patients in our review (Fig. 1). Two trials [25, 26] from the original review were excluded as they were identified as quasi-randomised (e.g. even/odd day allocation). The main reason for excluding trials was that included patients were not critically ill. We listed reasons for the exclusion of trials at full-text level. We identified 6 ongoing trials and 5 terminated trials with no results (Additional file 1).

### Characteristics of included trials

The included trials were published between 1996 and 2022, except for one trial that had unpublished data [27] (Table 1). This trial provided data for mortality and SAEs/SARs, but data on days alive without delirium or coma and QTc prolongation were not included as data were privileged until publication of the trial. The 11 trials included 15 comparisons. Of these, five trials used placebo as comparator [16, 27–30], five trials used other antipsychotics (chlorpromazine [31], ziprasidone [29], risperidone [32] and quetiapine [30, 33]), one trial used dexmedetomidine [34], one trial used benzodiazepines (lorazepam) [31], one trial used morphine [35], one trial used antiemetics (ondansetron) [34], and one trial used no intervention [36]. Two trials used haloperidol as rescue medication [34, 36], and five trials reported exposure to open-label antipsychotics during the trial intervention period [16, 27, 29, 34, 36].

Eight trials included patients admitted to an ICU, two trials included patients admitted to an ICU and emergency department or general ward, and one trial included patients from a high-dependency unit. The number of included patients ranged from 10 to 1000 patients. The mean age ranged from 31 to 71 years, and the proportion of females ranged from 9 to 47%.

**Table 1:** Characteristics of included trials

Trial	Country	No. of patients; no. of sites	Clinical setting	Intervention	Comparator	Duration of intervention	Outcomes
*Control intervention: Placebo*							
Andersen-Ranberg et al. [16]	Denmark, Finland, UK, Italy	1000; 18	Adult patients admitted to mixed ICUs	*Haloperidol (IV)* 2.5mg × 3 daily with as needed dosing to max 20mg/daily	*Placebo (IV)* matching intervention	Maximum 90 days	Mortality SAEs/SARs Days alive without delirium or coma QTc prolongation
EuRIDICE	The Netherlands	142; 8	Adult patients admitted to ICUs	*Haloperidol (IV)* 2.5-5mg × 3 daily, max dose 15mg/daily	*Placebo (IV)* matching intervention	Maximum 14 days	Mortality SAEs/SARs
Garg et al.[30]	India	45; 1	Adult patients admitted to an ICU	*Haloperidol (oral)* max dose 30mg/daily	*Placebo (oral)* matching intervention *Quetiapin (oral)* max dose 300mg	Maximum 14 days (unclear)	Mortality SAEs/SARs days alive without delirium or coma
Girard et al. [29]	USA	566; 16	Adult patients admitted to mixed ICUs	*Haloperidol (IV)* 1.25–2.5 × 2 daily max dose 10/20mg daily	*Placebo (IV):* matching intervention *Ziprasidone (IV):* 2.5–5mg × 2 daily, max dose 20/40mg	Maximum 14 days	Mortality SAEs/SARs Days alive without delirium or coma QTc prolongation
ORIC-I [28]	USA	30; 1	Adult, mechanically ventilated patients admitted to an ICU	*Haloperidol (IV)* 5mg × 2 daily	*Placebo (IV)* matching intervention	Maximum 14 days	Mortality SAEs/SARs QTc prolongation
*Control intervention: other antipsychotics*							
Talebi et al. (2022)	Iran	200; 1	Adult patients admitted to emergency department or ICU	*Haloperidol (IM)* 5mg × 2 daily adjusted according to clinical evaluation	*Quetiapin (oral)* 25mg × 2 daily adjusted according to clinical evaluation	3 days	Delirium severity
Han et al. [32]	Korea	28; 1	Adult patients admitted to 2 ICUs, 4 medical wards and 2 oncology wards	*Haloperidol (oral)* 0.75 mg × 2 daily increasing according to delirium symptoms. Mean dose during 7 days was 1.71mg	*Risperidone (oral)* 0.5 mg × 2 daily, increasing according to delirium symptoms. Mean dose during 7 days was 1.02mg	7 days	SAEs/SARs Delirium severity
Breitbart et al. [31]	USA	32; 1	AIDS patients admitted to high-dependency AIDS unit	*Haloperidol (oral/IM)* Dose according to delirium symptoms. Mean haloperidol dose the first 24h was 2.8mg. Average maintenance dose: 1.4mg	*Chlorpromazine (oral/IM)* Mean dose first 24 h was 50 mg. Average maintenance dose was 36mg *Lorazepam (oral/IM)* mean dose the first 24h was 3 mg. Average maintenance dose 4.6mg	Maximum 6 days	Mortality SAEs/SARs Delirium Severity Cognitive function QTc prolongation
*Control intervention: dexmedetomidine, antiemetic or no intervention*							
Bakri et al. [34]	Saudi Arabia	96; 1	Adult, patients admitted to trauma ICU	*Haloperidol (infusion)* 5mg twice daily	*Dexmedetomidine (infusion)* 1 μg/kg × 2 daily *Ondansetron (infusion)* 4mg × 2 daily	3 days	SAEs/SARs Delirium severity QTc prolongation
Atalan et al. [35]	Turkey	53; 1	Adult patients with hyperactive delirium admitted to cardiac ICU	*Haloperidol (IM)* 5 mg every hour until adequate RASS (-1 to + 1), max 20mg daily	*Morphine (IM)* 5mg every hour until adequate RASS (-1 to + 1), max 20mg daily	Maximum 10 days	Mortality SAEs/SARs
Graham et al. [36]	USA	10; 1	Adult, intubated patients admitted to an ICU	*Haloperidol (IV)* Titrated up to max 80mg daily	No intervention	NR	Mortality SAEs/SARs Cognitive function

### Haloperidol versus placebo

#### Primary outcomes

##### All-cause mortality

**Fig. 2:**
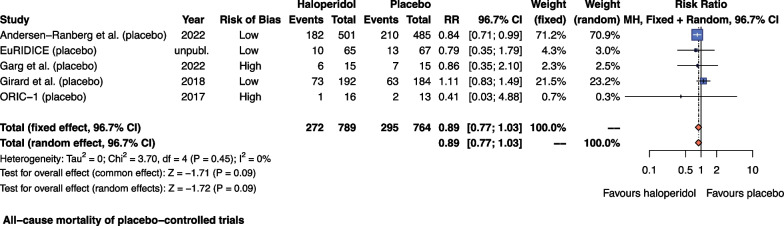
Forest plot of all-cause mortality in placebo-controlled trials. Forest plot of all-cause mortality in placebo-controlled trials. Three trials were at overall low risk of bias, and two trials were at overall high risk of bias. Size of the squares reflects the size of the trial (sample size). The horizontal bars represent 96.7% confidence intervals

**Fig. 3:**
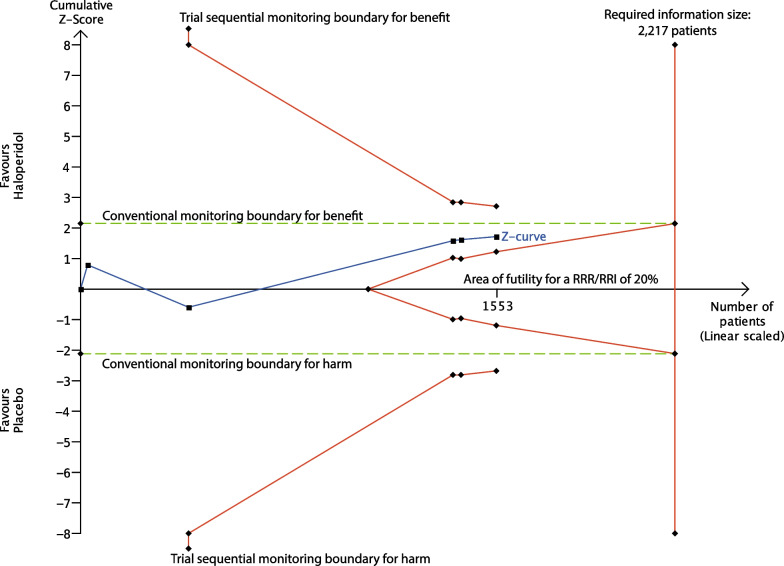
Trial sequential analysis of all-cause mortality in placebo-controlled trials. Trial sequential analysis of all-cause mortality for placebo-controlled trials (3 trials at overall low risk of bias and 2 trials at overall high risk of bias). We used a control event proportion of 38.6%, *α* of 3.3% (two-sided), *β* of 10% (power of 90%), diversity of 20% and a priori relative risk reduction or increase (RRR/RRI) of 20%. The *z*-curve (blue line) did not cross the trial sequential boundaries for benefit or harm (red outward sloping lines) or the inner-wedge futility line (red inward sloping red lines). The green dashed line shows the conventional boundaries for benefit/harm (alpha 0.033)

Five placebo-controlled trials (1553 patients, follow-up 28 to 90 days) reported on all-cause mortality. Three trials (1518 patients) were at overall low risk of bias (Aditional file 1; Fig. 1). The proportion of patients who died during follow-up was 272 of 789 (34.5%) in the haloperidol group and 295 of 764 (38.5%) in the placebo group. Meta-analysis (Fig. 2) showed no statistically significant difference in mortality between haloperidol and placebo (RR 0.89; 96.7% CI 0.77 to 1.03; *I*^2^ = 0%; TSA-adjusted CI 0.75 to 1.07). TSA showed that we had insufficient information to confirm or reject a 20% relative change (Fig. 3 and Additional file 1: Figure S5).

Subgroup analysis of trials at overall high risk of bias versus trials at overall low risk of bias was consistent with the primary analysis (test of interaction: *P* = 0.70, Additional file 1: Figure S2). No subgroup analysis was performed for patient population or delirium motor subtype as the included trials did not differ in these domains. Sensitivity analyses on missing data were consistent with the primary analysis (Additional file 1: Figure S3–S4). The certainty of evidence was judged to be moderate due to imprecision (Table 2).

**Table 2:** GRADE evaluation of the certainty of evidence

Certainty assessment							№ of patients		Effect		Certainty	Importance
№ of studies	Study design	Risk of bias	Inconsistency	Indirectness	Imprecision	Other considerations	Haloperidol	Placebo	Relative (96.7%/98% CI)*	Absolute (96.7%/98% CI)*		
*All-cause mortality (follow-up: range 28 days to 90 days)*												
5	Randomised trials	Not serious^a^	Not serious	Not serious	Serious^b^	None^c^	272/789 (34.5%)	295/764 (38.6%)	RR 0.89 (0.87 to 1.03)	42 fewer per 1.000 (from 50 fewer to 8 more)	⨁⨁⨁◯ Moderate	Critical
*SAE/SAR (highest proportion) (follow-up: range 3 days to 90 days)*												
5	Randomised trials	Not serious^a^	Serious^d^	Not serious	Serious^j^	None^c^	311/789 (39.4%)	331/764 (43.3%)	RR 0.94 (0.81 to 1.10)	26 fewer per 1.000 (from 82 fewer to 43 more)	⨁⨁◯◯ Low	Critical
*SAEs/SARs (cumulated events) (follow-up: range 3 days to 90 days)*												
5	Randomised trials	Not serious^a^	Serious^d,e^	Not serious	Very serious^f^	None^c^	426/789 (54.0%)	440/764 (57.6%)	RR 0.97 (0.85 to 1.11)	17 fewer per 1.000 (from 86 fewer to 63 more)	⨁◯◯◯ Very low	Critical
*Days alive without delirium or coma (follow-up: 14 days)*												
3	Randomised trials	Not serious^g^	Not serious	Not serious	Not serious^h^	Missing data have the potential to change evaluation^c^	7.2	6.8	–	MD 0.33 days more (0.31 fewer to 0.97 more)	⨁⨁⨁◯ Moderate	Important
*Delirium severity*												
0	Randomised trials								–	Not estimable	–	Important
*Cognitive function*												
0	Randomised trials								–	Not estimable	–	Critical
*Health-related quality of life*												
0	Randomised trials								–	Not estimable	–	Critical
*QTc prolongation (follow-up: range 3 days to 90 days)*												
3	Randomised trials	Not serious	Not serious	Not serious	Very serious^i^	None^c^	28/709 (3.9%)	18/683 (2.6%)	RR 1.47^§^ (0.78 to 2.76)	12 more per 1.000 (from 5 fewer to 43 more)	⨁⨁◯◯ Low	Important

CI, confidence interval; MD, mean difference; RR, risk ratio

*TSA-adjusted 96.7% CI for primary outcomes and TSA-adjusted 98% CI for secondary outcomes

^§^95% CI

^a^3 of 5 trials were at overall low risk of bias. Trials with high risk of bias had small sample size and did not have much influence on the pooled effect estimate

^b^TSA showed that we did not have sufficient information to confirm or reject a 20% RRR. The cumulative z-curve did not cross monitoring boundaries. The accrued information size was between 50 and 100% of required information size, and therefore downgraded 1 level

^c^3 trials comparing haloperidol with placebo were identified in trial registers. These trials were terminated or had status unknown and trial results were not available. These unpublished trials were not considered as serious publication bias

^d^Trials did not adhere to ICH-GCP. Some trials reported SAEs others reported SARs

^e^I^2^ was 87% (*P* < 0.01) for cumulated SAEs. Inconsistencies in number of events, but overlapping CIs

^f^The accrued information size was < 50% of the required information size estimated by TSA. No TSA monitoring boundaries were crossed. Therefore, we downgraded with 2 levels for imprecision

^g^3 of 4 trials were at low risk of bias and included the majority of patients

^h^TSA showed that the cumulative *z*-curve crossed the futility area with 80% of the required information size

^i^Accrued information was < 5% of the required information size. Downgraded 2 levels

^j^TSA reached futility for a RRR of 20%, but CI was wide and did include a potential 20% RRR but also a potential 10% RRI, indicating uncertainty. Downgraded 1 level

#### Serious adverse events and reactions (SAEs/SARs)

Five placebo-controlled trials (1553 patients, follow-up three to 90 days) reported on SAEs or SARs. Two trials were at overall low risk of bias. Details on reported SAEs/SARs are presented in Additional file 1: Table S2. All trials reported on mortality, but few specified this outcome as an SAE; we included mortality in the reported SAEs as we defined SAE according to ICH-GCP definition [37]. We found no statistically significant difference between haloperidol and placebo for the two estimates of SAEs/SARs measured as the SAE/SAR with the highest proportion (RR 0.94; 96.7% CI 0.81 to 1.10; *I*^2^ = 18%; TSA-adjusted 95% CI 0.78 to 1.14) and measured as cumulated SAEs/SARs (RR 0.97; 96.7% CI 0.85 to 1.11; *I*^2^ = 83%; TSA-adjusted CI 0.60 to 1.58) (Additional file 1: Figure S7 and S14). For SAE highest proportion, TSA reached the futility area meaning that haloperidol does not cause a 20% relative change. TSA for cumulated SAEs showed insufficient information to confirm or reject a relative change of 20% (Additional file 1: Figure S11 + S12 + S18).

The subgroup analysis of risk of bias and sensitivity analysis on missing data for both highest proportion and cumulated SAEs indicated that risk of bias and incomplete outcome data could influence the results (Additional file 1: Figure S8–S10 + Figure S15–S17). The certainty of evidence for SAE/SAR highest proportion was judged to be low due to inconsistency and imprecision. The certainty of evidence for cumulated SAEs/SARs was judged to be very low due to inconsistency and imprecision (Table 2).

#### Secondary outcomes

##### Days alive without delirium or coma (14 days)

Three trials (1349 patients) reported on days alive without delirium or coma. Two trials were at low risk of bias. Meta-analysis showed no statistically significant difference between haloperidol and placebo (MD 0.33 days; 98% CI − 0.31 to 0.97 days; *I*^2^ = 0%; TSA-adjusted CI − 0.41 to 1.08 days) (Additional file 1: Figure S20). TSA found that with 81% of the required information size, the cumulated z-curve crossed into the futility area; hence, haloperidol does not cause a 20% relative change in days alive without delirium or coma compared with placebo (Additional file 1: Figure S24 + S25). Subgroup analyses of risk of bias were consistent with the primary findings (test of interaction: *P* = 0.65, Additional file 1: Figure S21). Sensitivity analyses on missing data indicated that incomplete data alone had the potential to influence the results (Additional file 1: Figure S22-S23). The certainty of evidence was judged to be moderate due to the potential influence of missing data (Table 2).

#### Delirium severity, cognitive function and health-related quality of life

No placebo-controlled trials reported on delirium severity, cognitive function or health-related quality of life.

#### Explorative outcome

##### QTc prolongation

Three trials (1392 patients, follow-up three to 90 days) reported on QTc prolongation. Two trials were at overall low risk of bias. Twenty-eight patients (4%) assigned to haloperidol experienced QTc prolongation, while 18 patients (3%) assigned to placebo experienced QTc prolongation. Meta-analysis did not show a statistically significant difference between haloperidol and placebo (RR 1.47; 95% CI 0.83 to 2.64; *I*^2^ = 0) (Figure S31). TSA revealed that less than 5% of the required information size was accrued. Subgroup analyses on risk of bias were consistent with the primary findings (test of interaction: *P* = 0.63, Additional file 1: Figure S32). Sensitivity analyses on missing data were consistent with the primary analysis (Additional file 1: Figure S33-S34). The certainty of evidence was judged to be low due to imprecision (Table 2).

#### Haloperidol versus other comparators

A total of 5 trials with 664 patients compared haloperidol to other antipsychotics (chlorpromazine, ziprasidone, risperidone, quetiapine), and one trial was at overall low risk of bias for all reported outcomes. Meta-analysis on mortality (3 trials), SAEs/SARs (highest proportion and cumulated events; 4 trials), days alive without delirium or coma (2 trials) and delirium severity (3 trials) showed no statistically significant differences in these outcomes between haloperidol and other antipsychotics (Additional file 1: Figure S1, S7, S14, S20, S27, S28, S29). TSA on mortality, SAEs/SARs and days alive without delirium or coma found that less than 50% of the required information size was accrued to accept or reject a 20% change in these outcomes (Additional file 1: Figure S6, S13, S19, S26). Only one trial reported on cognitive function (Additional file 1: Figure S30) and QTc prolongation (Additional file 1: Figure S31). The certainty of evidence for all outcomes was judged either low or very low due to indirectness and imprecision.

For the comparators dexmedetomidine, benzodiazepines, morphine, antiemetics and no control, data could not be pooled as there was only one trial with each comparator (Additional file 1: Figure S1, S7, S14, S20, S27 and S31). Further details on haloperidol versus other comparators are available in Additional file 1.

## Discussion

In this systematic review of haloperidol versus placebo or any comparator for critically ill adult patients with delirium, we found that haloperidol may reduce mortality and likely results in little to no difference in the occurrence of SAEs/SARs compared with placebo. For the secondary outcomes, we found that haloperidol probably does not reduce or increase the number of days alive without delirium or coma and may result in little to no change in the occurrence of QTc prolongation. No placebo-controlled trials reported on delirium severity, cognitive function or health-related quality of life. Sparse data were available for haloperidol versus other comparators, and the effect of haloperidol on reported outcomes is either very uncertain or may result in little to no difference when compared with other comparators [24].

### Mortality

We chose mortality as one of our primary outcomes as it serves as a useful indicator for assessing the overall benefits and harms of an intervention in a population with high mortality. Delirium has been associated with increased mortality [6]; thus, interventions targeted at managing delirium may therefore potentially impact mortality.

The quantity and quality of data have increased significantly since the original review [10] as two RCTs with overall low risk of bias have provided data for the effect of haloperidol versus placebo on mortality. The effect estimate is in favour of haloperidol, but the pre-specified threshold for significance was not passed and TSA found that the required information size was not reached to firmly detect or reject a 20% relative change in mortality, and even more data are needed to establish firm evidence of smaller effect sizes as estimated in the meta-analysis (11% RRR). An anticipated 20% relative change in mortality may seem large as most interventional trials in critically ill patients find either small, clinically unimportant or statistically insignificant differences [38, 39]. More RCTs are therefore needed to establish firm evidence of the effect of haloperidol on mortality.

When we examine the meta-analysis for mortality of trials comparing haloperidol versus placebo, it is noticeable that the largest RCT (AID-ICU [16]) included in the review found benefit of haloperidol while the second largest RCT (MIND-USA [29]) indicated harm. Both trials are at overall low risk of bias. The opposing effect of haloperidol on mortality in the two trials may indicate that the effect of haloperidol differs dependent on patient population. Marked differences between the AID-ICU and MIND-USA trial populations were that patients in the AID-ICU trial were older and had more hyperactive delirium and fewer patients received mechanical ventilation.

### Serious adverse events and serious adverse reactions

The reporting of SAEs and SARs was heterogeneous, and few trials reported SAEs in accordance with ICH-GCP definitions. Some trials reported zero SAEs/SARs in both groups, yet did report mortality. Accordingly, SAEs/SARs are likely to be underreported. The pooled effect estimates of both measures of SAEs/SARs were rather similar and no significant differences were found. While we had sufficient information to reject a 20% relative change when SAEs/SARs were analysed as highest proportion, we had insufficient data when analysed as cumulated number of SAEs/SARs. This conflicting result is due to differences in proportions that affect the TSA analysis and as the true effect is expected to be between the two estimates, we cannot firmly detect or reject a 20% relative change in the proportion of patients experiencing SAEs/SARs when comparing haloperidol with placebo.

### Other systematic reviews

A comprehensive Cochrane review on pharmacological interventions for the treatment of delirium in critically ill adults was published in 2019 [40]. Data from two RCTs with overall low risk of bias have emerged since then, warranting an update. The Cochrane review included RCTs randomising patients with high risk of delirium; these trials were excluded in this review as patients were required to have diagnosed delirium at randomisation to be eligible for inclusion. Of note, we excluded the HOPE-ICU trial [41] and the feasibility MIND trial [42] as these trials randomised mechanically ventilated patients irrespective of delirium status. The Cochrane review found high-certainty evidence for no significant difference between typical antipsychotics (haloperidol) versus placebo on delirium duration, which is in line with our findings on days alive without delirium or coma for haloperidol versus placebo. The outcomes are not identical, but do measure similar events. Four trials were included in the meta-analysis for mortality in the Cochrane review, but only one was an actual treatment trial [29].

Other systematic reviews have been conducted in recent years [43–45], but like the Cochrane review, they included trials that randomised patients at risk of delirium or assessed the effects of haloperidol on preventing delirium. These systematic reviews are therefore evaluating the effects of haloperidol in populations that differ from this review that only included treatment trials.

### Implication for clinical practice and perspectives

A high number of critically ill patients develop delirium and haloperidol is still the most used pharmacological intervention [9]. The summarised evidence in this review indicates possible benefit on mortality and SAE/SAR, although uncertainty remains. A recent Bayesian analysis of the largest RCT included in this review, the AID-ICU trial, found high probability of benefit and low probability of harm on reported outcomes, most importantly 94% probability of a clinically important benefit (2% risk difference or more) on mortality [46]. Taken together, the available evidence does not indicate harm of haloperidol treatment, and it may be beneficial in critically ill adult patients with delirium. Moreover, it is currently the best studied antipsychotic in this population. Consequently, if strategies of prevention and non-pharmacological interventions fails, haloperidol is possibly a beneficial agent to use if pharmacological interventions are needed for the treatment of delirium. This statement is given as haloperidol is already frequently used in clinical practice, is well known to health-care personnel, and is easy to use and titrate.

### Strengths and limitations

We adhered to the Cochrane handbook, the PRISMA and the GRADE approach [12–14]. We published the protocol and updated the protocol registration in PROSPERO before conducting the literature search for this updated systematic review. We used the RoB 2 tool to assess risk of bias at outcome level. We used TSA to minimise the risk of random errors due to sparse data and multiple outcomes.

This systematic review also has limitations. First, five placebo-controlled trials provided data for our primary outcomes, but we still do not have sufficient data to firmly detect or reject a 20% relative change for our primary outcomes. Data were increasingly sparse for other comparators than placebo.

Second, a considerable number of trials reported exposure to open-label antipsychotics which may have contaminated the placebo group with antipsychotics and driven a potential intervention effect towards null. Third, we included trials randomising patients with diagnosed delirium and excluded trials randomising patients at high risk of delirium [40, 45]. We believe this is reasonable as we aim to examine the effect of treatment of delirium and not prevention, but with this approach, we may have lost information and power from these trials. Fourth none of the included trials reported on health-related quality of life or cognitive function. Both outcomes are highly patient-important and should be included in future trials as described in the core outcome set for delirium in critically ill patients [47]. Fifth, we planned to examine clinical heterogeneity by performing pre-defined subgroups, but data were not available to conduct such analyses.

## Conclusions

In this review, we found that haloperidol may reduce mortality and likely result in little to no change in the occurrence of SAEs/SARs in critically ill patients with delirium based on moderate- and low-certainty evidence, respectively. For other outcomes, the certainty of evidence ranged from very low to moderate. However, the results were not statistically significant and more trials are therefore needed to establish more certain evidence of the effect of haloperidol. Only sparse data were available for other comparators than placebo.

### Supplementary Information

**Additional file 1**. Haloperidol for the treatment of critically ill patients – updated SRMA and TSA.

## Data Availability

All data generated and/or analysed during the current study are included within the published article and its additional files.

## References

[1] Association AP. Diagnostic and statistical manual of mental disorders, Fifth edition. American Psychiatric Association; 2013. p. 991.

[2] Krewulak KD (2018). Incidence and prevalence of delirium subtypes in an adult ICU: a systematic review and meta-analysis. Crit Care Med.

[3] Salluh JI (2015). Outcome of delirium in critically ill patients: systematic review and meta-analysis. BMJ.

[4] Brummel NE (2014). Delirium in the ICU and subsequent long-term disability among survivors of mechanical ventilation. Crit Care Med.

[5] Pandharipande PP (2013). Long-term cognitive impairment after critical illness. N Engl J Med.

[6] Ely EW (2004). Delirium as a predictor of mortality in mechanically ventilated patients in the intensive care unit. JAMA.

[7] Devlin JW (2018). Clinical practice guidelines for the prevention and management of pain, agitation/sedation, delirium, immobility, and sleep disruption in adult patients in the ICU. Crit Care Med.

[8] la Cour KN (2022). Distribution of delirium motor subtypes in the intensive care unit: a systematic scoping review. Crit Care.

[9] Collet MO (2018). Prevalence and risk factors related to haloperidol use for delirium in adult intensive care patients: the multinational AID-ICU inception cohort study. Intensive Care Med.

[10] Barbateskovic M (2020). Haloperidol for the treatment of delirium in critically ill patients: a systematic review with meta-analysis and trial sequential analysis. Acta Anaesthesiol Scand.

[11] Barbateskovic M (2018). Haloperidol for delirium in critically ill patients—protocol for a systematic review. Acta Anaesthesiol Scand.

[12] Higgins JPT, Chandler J, Cumpston M, Li T, Page MJ, Welch VA (editors). Cochrane handbook for systematic reviews of interventions version 6.3 (updated February 2022). Cochrane, 2022. Available from www.training.cochrane.org/handbook. 2022.

[13] Moher D (2015). Preferred reporting items for systematic review and meta-analysis protocols (PRISMA-P) 2015 statement. Syst Rev.

[14] Schünemann H, Guyatt G, Oxman A. Handbook for grading the quality of evidence and the strength of recommendations using the GRADE approach. The GRADE Working Group. 2013.

[15] (EMA), E.M.A. summary of product characteristics Haldol. 2017 [cited 2023 May 22].

[16] Andersen-Ranberg NC (2022). Haloperidol for the treatment of delirium in ICU patients. N Engl J Med.

[17] Sterne JAC (2019). RoB 2: a revised tool for assessing risk of bias in randomised trials. BMJ.

[18] Harbord RM, Egger M, Sterne JA (2006). A modified test for small-study effects in meta-analyses of controlled trials with binary endpoints. Stat Med.

[19] Jakobsen JC (2014). Thresholds for statistical and clinical significance in systematic reviews with meta-analytic methods. BMC Med Res Methodol.

[20] Luo D (2018). Optimally estimating the sample mean from the sample size, median, mid-range, and/or mid-quartile range. Stat Methods Med Res.

[21] Wan X (2014). Estimating the sample mean and standard deviation from the sample size, median, range and/or interquartile range. BMC Med Res Methodol.

[22] Thorlund K (2012). Evolution of heterogeneity (I2) estimates and their 95% confidence intervals in large meta-analyses. PLoS ONE.

[23] Atkins D (2004). Grading quality of evidence and strength of recommendations. BMJ.

[24] Santesso N (2020). GRADE guidelines 26: informative statements to communicate the findings of systematic reviews of interventions. J Clin Epidemiol.

[25] Tagarakis GI (2012). Ondasetron versus haloperidol for the treatment of postcardiotomy delirium: a prospective, randomized, double-blinded study. J Cardiothorac Surg.

[26] Skrobik YK (2004). Olanzapine vs haloperidol: treating delirium in a critical care setting. Intensive Care Med.

[27] Smit L (2020). Efficacy of halopeRIdol to decrease the burden of delirium in adult critically ill patients (EuRIDICE): study protocol for a prospective randomised multi-centre double-blind placebo-controlled clinical trial in the Netherlands. BMJ Open.

[28] NCT00300391, ORIC-I: optimizing recovery from intensive care: mechanical ventilation and delirium.

[29] Girard TD (2018). Haloperidol and ziprasidone for treatment of delirium in critical illness. N Engl J Med.

[30] Garg R (2022). Comparison of haloperidol and quetiapine for treatment of delirium in critical illness: a prospective randomised double-blind placebo-controlled trial. J Clin Diagn Res.

[31] Breitbart W (1996). A double-blind trial of haloperidol, chlorpromazine, and lorazepam in the treatment of delirium in hospitalized AIDS patients. Am J Psychiatry.

[32] Han CS, Kim YK (2004). A double-blind trial of risperidone and haloperidol for the treatment of delirium. Psychosomatics.

[33] Doluee MT (2021). Comparison of the effectiveness of haloperidol injection and oral quetiapine to control delirium in patients in the emergency department and intensive care unit—a randomized clinical trial. Iran Red Crescent Med J.

[34] Bakri MI, Ibrahim E (2015). A comparison of dexmedetomidine or ondansetron with haloperidol for treatment of postoperative delirium in trauma patients admitted to intensive care unit: randomized controlled trial. Anaesth Pain Intensive Care.

[35] Atalan N (2013). Morphine is a reasonable alternative to haloperidol in the treatment of postoperative hyperactive-type delirium after cardiac surgery. J Cardiothorac Vasc Anesth.

[36] Graham BB, Douglas IS (2006). A prospective randomized study of haloperidol in addition to standard sedation in delirious and intubated patients: preliminary safety analysis. Crit Care Med.

[37] Guideline IH (2015). Integrated addendum to ICH E6 (R1): guideline for good clinical practice E6 (R2). Curr Step.

[38] Santacruz CA (2019). Which multicenter randomized controlled trials in critical care medicine have shown reduced mortality? A systematic review. Crit Care Med.

[39] Ridgeon EE (2017). Effect sizes in ongoing randomized controlled critical care trials. Crit Care.

[40] Burry L (2019). Pharmacological interventions for the treatment of delirium in critically ill adults. Cochrane Database Syst Rev.

[41] Page VJ (2013). Effect of intravenous haloperidol on the duration of delirium and coma in critically ill patients (Hope-ICU): a randomised, double-blind, placebo-controlled trial. Lancet Respir Med.

[42] Girard TD (2010). Feasibility, efficacy, and safety of antipsychotics for intensive care unit delirium: the MIND randomized, placebo-controlled trial. Crit Care Med.

[43] Marra A (2021). Haloperidol for preventing delirium in ICU patients: a systematic review and meta-analysis. Eur Rev Med Pharmacol Sci.

[44] Chen Z (2020). Efficacy and safety of haloperidol for delirium prevention in adult patients: an updated meta-analysis with trial sequential analysis of randomized controlled trials. J Clin Anesth.

[45] Zayed Y (2019). Haloperidol for the management of delirium in adult intensive care unit patients: a systematic review and meta-analysis of randomized controlled trials. J Crit Care.

[46] Andersen-Ranberg NC (2023). Haloperidol vs placebo for the treatment of delirium in ICU patients: a pre-planned, secondary Bayesian analysis of the AID–ICU trial. Intensive Care Med.

[47] Rose L (2021). A core outcome set for research evaluating interventions to prevent and/or treat delirium in critically ill adults: an international consensus study (Del-COrS). Crit Care Med.

