# Supplementary material for: Haloperidol for the treatment of delirium in critically ill patients: an updated systematic review with meta-analysis and trial sequential analysis
Source: Crit Care. 2023 Aug 26;27:329. doi: 10.1186/s13054-023-04621-4 (PMC10463604; doi:10.1186/s13054-023-04621-4)
Supplement: Supplementary file 1 — Additional file 1. Haloperidol for the treatment of critically ill patients – updated SRMA and TSA. [file 13054_2023_4621_MOESM1_ESM.pdf]

Haloperidol for the Treatment of Delirium in Critically Ill Patients - an updated Systematic Review with Meta-analysis and Trial Sequential Analysis

Electronic Supplementary Material

Table of content

Haloperidol versus other antipsychotics 4

Haloperidol versus dexmedetomidine 5

Haloperidol versus benzodiazepines 5

Haloperidol versus opioids 5

Haloperidol versus antiemetics 5

Haloperidol versus no control 5

Details on sensitivity analyses: best/worst-case and worst/best-case scenarios 7

Search strategies 8

Details of included trials and risk of bias assessments 13

Selected excluded trials 29

Ongoing and terminated trials 31

Risk of bias assessments of outcomes 33

Table S1: GRADE summary of findings for haloperidol versus other antipsychotics 36

Figure S1: All-cause mortality; haloperidol versus all comparators 39

Figure S2: Subgroup analysis of mortality for haloperidol versus placebo; low-risk of bias versus high-risk of bias trials 40

Figure S3: Sensitivity analysis best-worst case scenario of all-cause mortality 41

Figure S4: Sensitivity analysis worst-best case scenario of all-cause mortality 42

Figure S5: TSA sensitivity analysis of all-cause mortality for haloperidol versus placebo (RR11%) 43

Figure S6: TSA of all-cause mortality for haloperidol versus other antipsychotics 44

Figure S7: SAE/SAR highest proportion; haloperidol versus all comparators 45

Figure S8: Subgroup analysis of SAE/SAR highest proportion for haloperidol versus placebo; low-risk of bias versus high-risk of bias trials 46

Figure S9: Sensitivity analysis best-worst case scenario of SAE/SAR highest proportion 47

Figure S10: Sensitivity analysis worst-best case scenario of SAE/SAR highest proportion 48

Figure S11: TSA of SAE/SAR highest proportion for haloperidol versus placebo (RR20%) 49

Figure S12: TSA sensitivity analysis of SAE/SAR highest proportion for haloperidol versus placebo (RR 6%) 50

Figure S13: TSA of SAE/SAR for haloperidol versus other antipsychotics 51

Figure S14: cumulated SAEs/SARs; haloperidol versus all comparators 52

Figure S15: Subgroup analysis of cumulated SAEs/SARs for haloperidol versus placebo; low-risk of bias versus high-risk of bias trials 53

Figure S16: Sensitivity analysis best-worst case scenario of cumulated SAEs/SARs 54

Figure S17: Sensitivity analysis worst-best case scenario of cumulated SAEs/SARs 55

Figure S18: TSA of cumulated SAEs/SARs for haloperidol versus placebo 56

Figure S19: TSA of cumulated SAEs/SARs for haloperidol versus other antipsychotics 57

Figure S20: Days alive without delirium or coma (14 days); haloperidol versus all comparators 58

Figure S21: Subgroup analysis of *Days alive without delirium or coma (14 days)* for haloperidol versus placebo; low-risk of bias versus high-risk of bias trials 59

Figure S22: Sensitivity analysis best-worst case scenario of *Days alive without delirium or coma*. 60

Figure S23: Sensitivity analysis worst-best case scenario of *Days alive without delirium or coma*. 61

Figure S24: TSA of Days alive without delirium or coma (14 days) for haloperidol versus placebo 62

Figure S25: TSA sensitivity analysis of Days alive without delirium or coma (14 days) for haloperidol versus placebo (MD=0.33) 63

Figure S26: TSA of Days alive without delirium or coma (14 days) for haloperidol versus other antipsychotics 64

Figure S27: Delirium Severity; haloperidol versus all comparators 65

Figure S28: Sensitivity analysis best-worst case scenario of Delirium Severity 66

Figure S29: Sensitivity analysis worst-best case scenario of Delirium Severity 67

Figure S30: Cognitive function; haloperidol versus all comparators 68

Figure S31: QTc prolongation; haloperidol versus all comparators 69

Figure S32: Subgroup analysis of QTc prolongation for haloperidol versus placebo; low-risk of bias versus high-risk of bias trials 70

Figure S33: Sensitivity analysis best-worst case scenario of QTc prolongation **71**

Figure S34: Sensitivity analysis worst-best case scenario of QTc prolongation **72**

Table S2. Individual Serious Adverse Events and Serous Adverse Reactions reported in included trials Fejl! Bogmærke er ikke defineret.

Haloperidol versus other antipsychotics

Mortality

Three trials with a total of 436 patients reported on mortality when comparing haloperidol to other antipsychotics. The control intervention was either chlorpromazine, quetiapine or ziprasidone. One trial was of low risk of bias. We found no statistically significant difference in mortality when meta-analysing data from the three trials (RR 1.07; 96.7%CI 0.82-1.41; I^2=0%; TSA-adjusted CI 0.38– 2.97) (Figure S1). TSA indicated that 16% of the required information size was accrued (Figure S6). The quality of evidence was judged to very low due to indirectness and imprecision (Table S1).

SAEs/SARs

Four trials with a total of 460 patients reported on SAEs/SARs. The control intervention was either chlorpromazine, quetiapine or ziprasidone and one trial was of low risk of bias. All trials reported on mortality but few specified this outcome as an SAE. We categorised mortality as an SAE in accordance with International Conference on Harmonisation good clinical practice. In the two analyses estimating the number of patients with one or more SAEs/SARs we found no statistically significant difference between haloperidol and other antipsychotics (highest proportion: RR 1.06; 96.7% CI 0.81-1.40; I^2 = 0%; TSA adjusted CI 0.38 – 3.00; P = 0.58, cumulated: RR 1.05; 96.7%CI 0.93-1.18; I^2=0%; TSA adjusted CI 0.85 – 1.33; P=0.32) (Figure S7 + S14). TSA for both SAE/SAR measures indicated that less than 50% of the required information size were accrued, and the cumulative z-curve did not cross any TSA monitoring boundaries. The quality of evidence was judged to very low for both SAE/SAR measures due to indirectness and imprecision (Table S1).

Days alive without delirium or coma (14 days)

Two trials with a total of 302 patients reported on days alive without delirium or coma. The comparators were either ziprasidone or quetiapine, 1 trial was of low risk of bias. We found no statistically significant difference in days alive without delirium or coma between haloperidol and other antipsychotics (mean difference (MD): -0.15; 98%CI -1.83 to 1.53; I^2=0%; TSA-adjusted CI: -2.49 to 2.10; P=0.75). TSA indicated that less than 50% of the required information size was accrued and no monitoring boundaries were crossed. The quality of evidence was judged to low due to indirectness and imprecision (Table S1).

Delirium Severity

Three trials with a total of 248 patients reported on delirium severity. All trials were of high risk of bias. The comparators were chlorpromazine, risperidone or quetiapine. We found no statistically significant difference in delirium severity between haloperidol and other antipsychotics (standardized mean difference (SMD): 0.01; 98%CI -0.29 to 0.31; I^2=0%; TSA-adjusted CI: too little information to conduct TSA) (Figure S27). The quality of evidence was judged to very low due to risk of bias, indirectness and imprecision (Table S1).

Health-related quality of life (HRQL), Cognitive function and QTc prolongation
No trials reported on HRQL. 1 trial reported on cognitive function, this trial included 24 patients and had chlorpromazine as comparator (Figure S30). 1 trial reported on QTc prolongation, this trial included 376 patients and had ziprasidone as comparator (Figure S31). No statistically significant differences were found for any of these outcomes and the quality of evidence were judged to very low for both outcomes (Table S1).

Haloperidol versus dexmedetomidine
Only 1 trial examined the effect of haloperidol versus dexmedetomidine. This trial included 64 patients and was judged high risk of bias for all reported outcomes; SAEs/SARs, delirium severity and QTc prolongation. No statistically significant differences were found for any of the reported outcomes for haloperidol versus dexmedetomidine (Figure S7+S14+S27+S30). Quality of evidence was judged very low for all outcomes due to risk of bias, imprecision and suspected publication bias (not shown).

Haloperidol versus benzodiazepines
Only 1 trial examined the effect of haloperidol versus benzodiazepines more specifically lorazepam. This trial included 17 patients and was judged high risk of bias for all reported outcomes; mortality, SAEs/SARs, delirium severity and cognitive function. No statistically significant differences were found for any of the reported outcomes for haloperidol versus lorazepam (Figure S1+S7+S14+S27+S30). Quality of evidence was judged very low for all outcomes due to risk of bias, imprecision and suspected publication bias (not shown).

Haloperidol versus opioids
Only 1 trial examined the effect of haloperidol versus opioids more specifically morphine. This trial included 53 patients and was judged high risk of bias for all reported outcomes; mortality and SAEs/SARs. No statistically significant differences were found for any of the reported outcomes for haloperidol versus lorazepam (Figure S1+S7+S14). Quality of evidence was judged very low for all outcomes due to risk of bias, imprecision and suspected publication bias (not shown).

Haloperidol versus antiemetics
Only 1 trial examined the effect of haloperidol versus antiemetics more specifically ondansetron. This trial included 64 patients and was judged high risk of bias for all reported outcomes; SAEs/SARs, delirium severity and QTc prolongation. No statistically significant differences were found for any of the reported outcomes for haloperidol versus antiemetics (Figure S7+S14+S27+S30). Quality of evidence was judged very low for all outcomes due to risk of bias, imprecision and suspected publication bias (not shown).

Haloperidol versus no control
Only 1 trial examined the effect of haloperidol versus no control. This trial included 10 patients and was judged high risk of bias for all reported outcomes; mortality, SAEs/SARs, delirium severity, cognitive function and QTc prolongation. No statistically significant differences were found for any

of the reported outcomes for haloperidol versus no control (Figure S1+S7+S14+S30+S31). Quality of evidence was judged very low for all outcomes due to risk of bias, imprecision and suspected publication bias (not shown).

Details on sensitivity analyses: best/worst-case and worst/best-case scenarios

To assess the potential impact of missing data for dichotomous outcomes, the two following analyses will be performed:

1. ’Best-worst-case’ scenario: It will be assumed that all participants lost to follow up in the experimental group survived, had no serious adverse event, and had no morbidity; and all those with missing outcomes in the control group did not survive, had a serious adverse event, and had morbidity.
2. ’Worst-best-case’ scenario: It will be assumed that all participants lost to follow-up in the experimental group did not survive, had a serious adverse event, and had morbidity; and all those with missing outcomes in the control group did survive, had no serious adverse event, and had no morbidity.

Results from both scenarios will be presented in the review.

To assess the potential impact of the missing data for continuous outcomes, the two following analyses will be performed:

1. ’Best-worst-case’ scenario: It will be assumed that all participants lost to follow up in the experimental group had mean (from patients with follow-up) + 2 x SD; and all those with missing outcomes in the control group had mean (from patients with follow-up) minus 2 x SD.
2. ’Worst-best-case’ scenario: It will be assumed that all participants lost to follow-up in the experimental group had mean (from patients with follow-up) minus 2 x SD; and all those with missing outcomes in the control group had mean (from patients with follow-up) + 2 x SD.

To assess the potential impact of missing SDs for continuous outcomes, the following sensitivity analyses will be performed: Where SDs are missing and not possible to calculate, SDs will be imputed from trials with similar populations and low risk of bias. If no such trials can be found, SDs will be imputed from trials with a similar population. As the final option SDs will be imputed from all trials.

Search strategies

Cochrane Central Register of Controlled Trials (CENTRAL)
(The Cochrane Library, from inception to April 18, 2023)

#1 MeSH descriptor: [Haloperidol]
#2 ((alased) or (aloperidin) or (aloperidine) or (apo-haloperidol) or (avant) or (binison) or (brotopon) or (celenase) or (cereen) or (cerenace) or (cizoren) or (depidol) or (dores) or (dozic) or (duraperidol) or (einalon s) or (fortunan) or (govotil) or (Haldol) or (haldol solutab) or (halidol) or (halo-p) or (halojust) or (halomed) or (haloneural) or (haloper) or (haloperidol) or (haloperidol hydrochoride) or (haloperidol intensol) or (haloperidol lactate) or (haloperil) or (haloperin) or (haloperitol) or (halopidol) or (halopol) or (halosten) or (haricon) or (haridol-d) or (keselan) or (linton) or (lodomer-2) or (mcn jr 1625) or (mcn jr1625) or (mixidol) or (novoperidol) or (nsc 170973) or (nsc170973) or (peluces) or (perida) or (peridol) or (peridor) or (r 1625) or (r1625) or (selezyme) or (seranace) or (serenace) or (serenase) or (serenelfi) or (siegoperidol) or (sigaperidol) or (trancodol-10) or (trancodol-5))
#3 (#1 or #2)
#4 MeSH descriptor: [Delirium] explode all trees
#5 deliri*
#6 (acute organic near (psychosyndrome* or brain syndrome*))
#7 (acute brain near (dysfunction* or failure* or syndrome*))
#8 ((postoperati* or post-operati* or postsurg* or post-surg*) near/1 (cognitive dysfunction or brain dysfunction or psychosis))
#9 ((psycho-organic syndrome* or psychoorganic syndrome* or organic psychosyndrome* or organic psycho-syndrome*) near/3 acute)
#10 (metabolic encephalopathy or exogenous psychosis)
#11 (acute confusion* or acute psycho-organic syndrome or obnubilat*)
#12 (clouded state or clouding of consciousness*)
#13 (cloud* near/3 consciousness*)
#14 (toxic near/1 (psychosis or confusion))
#15 (#4 or #5 or #6 or #7 or #8 or #9 or #10 or #11 or #12 or #13 or #14)
#16 (#3 and #15)

MEDLINE (Ovid),
From 1950 to April 18, 2023.
1. exp Haloperidol/
2. ((alased) or (aloperidin) or (aloperidine) or (apo-haloperidol) or (avant) or (binison) or (brotopon) or (celenase) or (cereen) or (cerenace) or (cizoren) or (depidol) or (dores) or (dozic) or (duraperidol) or (einalon s) or (fortunan) or (govotil) or (Haldol) or (haldol solutab) or (halidol) or (halo-p) or (halojust) or (halomed) or (haloneural) or (haloper) or (haloperidol) or (haloperidol hydrochoride) or (haloperidol intensol) or (haloperidol lactate) or (haloperil) or (haloperin) or (haloperitol) or (halopidol) or (halopol) or (halosten) or (haricon) or (haridol-d) or (keselan) or (linton) or (lodomer-2) or (mcn jr 1625) or (mcn jr1625) or (mixidol) or (novoperidol) or (nsc 170973) or (nsc170973) or (peluces) or (perida) or (peridol) or (peridor) or (r 1625) or (r1625) or (selezyme) or (seranace) or (serenace) or (serenase) or (serenelfi) or (siegoperidol) or (sigaperidol) or (trancodol-10) or (trancodol-5)).mp.
3. 1 or 2
4. exp Delirium/
5. deliri*.mp.
6. (acute organic adj (psychosyndrome* or brain syndrome*)).mp.
7. (acute brain adj (dysfunction* or failure* or syndrome*)).mp.

8. ((postoperati* or post-operati* or postsurg* or post-surg*) adj1 (cognitive dysfunction or brain dysfunction or psychosis)).mp.
9. ((psycho-organic syndrome* or psychoorganic syndrome* or organic psychosyndrome* or organic psycho-syndrome*) adj3 acute).mp.
10. (metabolic encephalopathy or exogenous psychosis).mp.
11. (acute confusion* or acute psycho-organic syndrome or obnubilat*).mp.
12. (clouded state or clouding of consciousness*).mp.
13. (cloud* adj3 consciousness*).mp.
14. (toxic adj1 (psychosis or confusion)).mp.
15. 4 or 5 or 6 or 7 or 8 or 9 or 10 or 11 or 12 or 13 or 14
16. 3 and 15

EMBASE (OvidSP)
From 1974 to April 18, 2023
1. *haloperidol/
2. *haloperidol decanoate/
3. *haloperidol-induced catalepsy/
4. *reduced haloperidol/
5. (alased or aloperidin or aloperidine or apo-haloperidol or avant or binison or brotopon or celenase or cereen or cerenace or cizoren or depidol or dores or dozic or duraperidol or einalon s or fortunan or govotil or Haldol or haldol solutab or halidol or halo-p or halojust or halomed or haloneural or haloper or haloperidol or haloperidol hydrochoride or haloperidol intensol or haloperidol lactate or haloperil or haloperin or haloperitol or halopidol or halopol or halosten or haricon or haridol-d or keselan or linton or lodomer-2 or mcn jr 1625 or mcn jr1625 or mixidol or novoperidol or nsc 170973 or nsc170973 or peluces or perida or peridol or peridor or r 1625 or r1625 or selezyme or seranace or serenace or serenase or serenelfi or siegoperidol or sigaperidol or trancodol-10 or trancodol-5).ti,ab,kw,tw.
6. 1 or 2 or 3 or 4 or 5
7. *delirium/
8. *postoperative delirium/
9. deliri*.ti,ab,kw.
10. (acute organic adj (psychosyndrome* or brain syndrome*)).ti,ab,kw.
11. (acute brain adj (dysfunction* or failure* or syndrome*)).ti,ab,kw.
12. ((postoperati* or post-operati* or postsurg* or post-surg*) adj1 (cognitive dysfunction or brain dysfunction or psychosis)).ti,ab,kw.
13. ((psycho-organic syndrome* or psychoorganic syndrome* or organic psychosyndrome* or organic psycho-syndrome*) adj3 acute).ti,ab,kw.
14. (metabolic encephalopathy or exogenous psychosis).ti,ab,kw.
15. (acute confusion* or acute psycho-organic syndrome or obnubilat*).ti,ab,kw.
16. (clouded state or clouding of consciousness*).ti,ab,kw.
17. (cloud* adj3 consciousness*).ti,ab,kw.
18. (toxic adj1 (psychosis or confusion)).ti,ab,kw.
19. 7 or 8 or 9 or 10 or 11 or 12 or 13 or 14 or 15 or 16 or 17 or 18
20. 6 and 19

Science Citation Index (web of science)
From 1900 to April 18, 2023.
#1 TOPIC: (((alased) or (aloperidin) or (aloperidine) or (apo-haloperidol) or (avant) or (binison) or (brotopon) or (celenase) or (cereen) or (cerenace) or (cizoren) or (depidol) or (dores) or (dozic) or (duraperidol) or (einalon s) or (fortunan) or (govotil) or (Haldol) or (haldol solutab) or (halidol) or (halo-p) or (halojust) or (halomed) or (haloneural) or (haloper) or (haloperidol) or (haloperidol hydrochoride) or

(haloperidol intensol) or (haloperidol lactate) or (haloperil) or (haloperin) or (haloperitol) or (halopidol) or (halopol) or (halosten) or (haricon) or (haridol-d) or (keselan) or (linton) or (lodomer-2) or (mcn jr 1625) or (mcn jr1625) or (mixidol) or (novoperidol) or (nsc 170973) or (nsc170973) or (peluces) or (perida) or (peridol) or (peridor) or (r 1625) or (r1625) or (selezyme) or (seranace) or (serenace) or (serenase) or (serenelfi) or (siegoperidol) or (sigaperidol) or (trancodol-10) or (trancodol-5)))
#2 TOPIC: (deliri*)
#3 TI=(acute organic and psychosyndrome* or brain syndrome*)
#4 TI=(acute brain and dysfunction* or failure* or syndrome*)
#5 TI=(postoperati* or post-operati* or postsurg* or post-surg* and cognitive dysfunction or brain dysfunction or psychosis)
#6 TS=(psycho-organic syndrome* or psychoorganic syndrome* or organic psychosyndrome* or organic psycho-syndrome* near/3 acute)
#7 TS=(metabolic encephalopathy or exogenous psychosis)
#8 TS=(acute confusion* or acute psycho-organic syndrome or obnubilat*)
#9 TI=(clouded state or clouding of consciousness*)
#10 TS=(cloud* near/3 consciousness*)
#11 TI=(toxic and psychosis or confusion)
#12 (#11 OR #10 OR #9 OR #8 OR #7 OR #6 OR #5 OR #4 OR #3 OR #2)
#13 (#12 AND #1)

Biosis Previews (web of science)
From 1969 to April 18, 2023.
#1 TOPIC: (((alased) or (aloperidin) or (aloperidine) or (apo-haloperidol) or (avant) or (binison) or (brotopon) or (celenase) or (cereen) or (cerenace) or (cizoren) or (depidol) or (dores) or (dozic) or (duraperidol) or (einalon s) or (fortunan) or (govotil) or (Haldol) or (haldol solutab) or (halidol) or (halo-p) or (halojust) or (halomed) or (haloneural) or (haloper) or (haloperidol) or (haloperidol hydrochoride) or (haloperidol intensol) or (haloperidol lactate) or (haloperil) or (haloperin) or (haloperitol) or (halopidol) or (halopol) or (halosten) or (haricon) or (haridol-d) or (keselan) or (linton) or (lodomer-2) or (mcn jr 1625) or (mcn jr1625) or (mixidol) or (novoperidol) or (nsc 170973) or (nsc170973) or (peluces) or (perida) or (peridol) or (peridor) or (r 1625) or (r1625) or (selezyme) or (seranace) or (serenace) or (serenase) or (serenelfi) or (siegoperidol) or (sigaperidol) or (trancodol-10) or (trancodol-5)))
#2 TOPIC: (deliri*)
#3 TI=(acute organic and psychosyndrome* or brain syndrome*)
#4 TI=(acute brain and dysfunction* or failure* or syndrome*)
#5 TI=(postoperati* or post-operati* or postsurg* or post-surg* and cognitive dysfunction or brain dysfunction or psychosis)
#6 TS=(psycho-organic syndrome* or psychoorganic syndrome* or organic psychosyndrome* or organic psycho-syndrome* near/3 acute)
#7 TS=(metabolic encephalopathy or exogenous psychosis)
#8 TS=(acute confusion* or acute psycho-organic syndrome or obnubilat*)
#9 TI=(clouded state or clouding of consciousness*)
#10 TS=(cloud* near/3 consciousness*)
#11 TI=(toxic and psychosis or confusion)
#12 (#11 OR #10 OR #9 OR #8 OR #7 OR #6 OR #5 OR #4 OR #3 OR #2)
#13 (#12 AND #1)

Cumulative Index to Nursing & Allied Health Literature (CINAHL)
From inception to April 18, 2023.
S1 MW haloperidol
S2 TX ((alased) or (aloperidin) or (aloperidine) or (apo-haloperidol) or (avant) or (binison) or (brotopon) or

(celenase) or (cereen) or (cerenace) or (cizoren) or (depidol) or (dores) or (dozic) or (duraperidol) or (einalon s) or (fortunan) or (govotil) or (Haldol) or (haldol solutab) or (halidol) or (halo-p) or (halojust) or (halomed) or (haloneural) or (haloper) or (haloperidol) or (haloperidol hydrochoride) or (haloperidol intensol) or (haloperidol lactate) or (haloperil) or (haloperin) or (haloperitol) or (halopidol) or (halopol) or (halosten) or (haricon) or (haridol-d) or (keselan) or (linton) or (lodomer-2) or (mcn jr 1625) or (mcn jr1625) or (mixidol) or (novoperidol) or (nsc 170973) or (nsc170973) or (peluces) or (perida) or (peridol) or (peridor) or (r 1625) or (r1625) or (selezyme) or (seranace) or (serenace) or (serenase) or (serenelfi) or (siegoperidol) or (sigaperidol) or (trancodol-10) or (trancodol-5))
S3 (S1 OR S2)
S4 MW delirium
S5 TX deliri*
S6 AB (acute organic and (psychosyndrome* or brain syndrome*))
S7 AB (acute brain and (dysfunction* or failure* or syndrome*))
S8 AB ((postoperati* or post-operati* or postsurg* or post-surg*) and (cognitive dysfunction or brain dysfunction or psychosis))
S9 AB ((psycho-organic syndrome* or psychoorganic syndrome* or organic psychosyndrome* or organic psycho-syndrome*) and acute)
S10 AB metabolic encephalopathy or exogenous psychosis
S11 AB acute confusion* or acute psycho-organic syndrome or obnubilat*
S12 AB clouded state or clouding of consciousness*
S13 AB cloud* and consciousness*
S14 AB (toxic and (psychosis or confusion))
S15 (S4 OR S5 OR S6 OR S7 OR S8 OR S9 OR S10 OR S11 OR S12 OR S13 OR S14)
S16 (S3 AND S15)

Latin American Caribbean Health Sciences Literature (LILACS),
From inception to April 18, 2023.
(alased) or (aloperidin) or (aloperidine) or (apo-haloperidol) or (avant) or (binison) or (brotopon) or (celenase) or (cereen) or (cerenace) or (cizoren) or (depidol) or (dores) or (dozic) or (duraperidol) or (einalon s) or (fortunan) or (govotil) or (Haldol) or (haldol solutab) or (halidol) or (halo-p) or (halojust) or (halomed) or (haloneural) or (haloper) or (haloperidol) or (haloperidol hydrochoride) or (haloperidol intensol) or (haloperidol lactate) or (haloperil) or (haloperin) or (haloperitol) or (halopidol) or (halopol) or (halosten) or (haricon) or (haridol-d) or (keselan) or (linton) or (lodomer-2) or (mcn jr 1625) or (mcn jr1625) or (mixidol) or (novoperidol) or (nsc 170973) or (nsc170973) or (peluces) or (perida) or (peridol) or (peridor) or (r 1625) or (r1625) or (selezyme) or (seranace) or (serenace) or (serenase) or (serenelfi) or (siegoperidol) or (sigaperidol) or (trancodol-10) or (trancodol-5) [Words] and (delirium) or (delirious) OR (acute organic psychosyndrome) or (acute organic brain syndrome) OR (acute brain dysfunction) or (acute brain failure) or (acute brain syndrome) OR (postoperative cognitive dysfunction) or (post-operative cognitive dysfunction) or (postsurgical cognitive dysfunction) or (post-surgical cognitive dysfunction) OR (postoperative brain dysfunction) or (post-operative brain dysfunction) or (postsurgical brain dysfunction) or (post-surgical brain dysfunction) OR (postoperative psychosis) or (post-operative psychosis) or (postsurgical psychosis) or (post-surgical psychosis) OR (acute psycho-organic syndrome) or (acute psychoorganic syndrome) or (acute organic psychosyndrome) or (acute organic psycho-syndrome) OR (metabolic encephalopathy) or (exogenous psychosis) OR (acute confusion) or (acute psycho-organic syndrome) or (obnubilate) OR (clouded state) or (clouding of consciousness) OR (toxic psychosis) or (toxic confusion) [Words]

We also screened the following registries for ongoing or terminated trials: ClinicalTrials.gov; WHO International Clinical Trials Registry Platform (ICTRP); EU clinical trial register (EUDRACT); and the Australian New Zealand Clinical Trials Registry (ANZCTR). We manually searched reference lists of

previous meta-analyses and included trials to identify any additional relevant trials not identified by the electronic searches. The US Food and Drug Administration (FDA); European Medicines Agency (EMA) and websites of medical companies were searched for unpublished trials.

Details of included trials and risk of bias assessments

AID-ICU 2022	
**Methods**	Randomised clinical trial<br><br>Study took place in 18 mixed ICUs. Patients were primarily recruited at Danish centres. Finland, Italy and United Kingdom contributed with a total of 36 patients.<br><br>Study duration: June 2018 – April 2022<br><br>Trial registration: NCT03392376
**Participants**	**Sample size:** 1000 patients<br><br>**Age (median):** 70.5 years<br><br>**Sex:** 34.2% females<br><br>**Baseline disease severity:** Predicted mortality risk at 90 days was 34.7%, calculated with the SMS-ICU score.<br><br>**Co-morbidities:**<br>- Tobacco use: haloperidol 31.2%; placebo 30.2%<br>- Alcohol overconsumption: haloperidol 17.0%; placebo 15.8%<br>- Patients on mechanical ventilation (invasive+noninvasive): haloperidol 63.9%; placebo 62.8%<br>- Patients on vasopressors or inotropes: haloperidol 54.3; placebo 49.2%<br>- Patients receiving renal replacement therapy: haloperidol 15.4%; placebo 14.4%<br>- Delirium motor subtype: hypoactive delirium 55.7%; hyperactive 44.3%<br><br>**Setting:** Adult critically ill patients admitted to mixed ICUs<br><br>**Inclusion criteria:** Adult patient, acutely admitted to the ICU with diagnosed delirium.<br><br>**Exclusion criteria:** Patients with known contraindications to haloperidol, habitual treatment with antipsychotics, received antipsychotics in the ICU prior to screening, permanently incompetent, delirium assessment non-applicable, withdrawn from active therapy or brain dead, pregnancy, consent unobtainable, admitted under coercive measures, delirium tremens.<br><br>**Delirium screening**: Delirium screening was performed by clinicians primarily ICU nurses with CAM-ICU or ICDSC. Screening was performed twice daily.
**Interventions**	**Experimental intervention:** intravenous haloperidol 2.5mg x 3 daily with as needed doses up to a maximum of 20mg daily as long as patients were delirious.<br><br>**Control intervention:** matching intravenous placebo (isotonic saline)

	**Duration:** 90 day intervention period. Patients received the intervention until they were delirium free (2 negative delirium screenings on the same day), discharged from the ICU or for a maximum of 90 days. In case of recurrence of delirium in the ICU intervention was resumed.<br><br>**Open-label antipsychotics:** Not allowed to be given to trial participants in the ICU during the 90-day intervention period. Participants that were withdrawn from the trial could receive open-label antipsychotics. Exposure to open-label antipsychotics (any antipsychotic) in the trial population was 13.1%.
**Outcomes**	**Outcomes:**<br><br>- Days alive and out of hospital to day 90<br>- 90-day mortality<br>- Hospital length of stay to day 90<br>- Days alive without delirium or coma<br>- Serious Adverse Reaction in ICU<br>- Number of patients using of rescue medication<br>- Days with rescue medication per patient<br>All outcomes were assessed at day 90
**Funding**	Innovation Funds Denmark, The Regional Medicines Fund, The Zealand region Research Fund, Intensiv Symposium Hindsgavl, Fogts Foundation.
**Notes**	Risk of bias assessment was performed by Mathieu Van der Jagt as the author (NCAR) was involved in the AID-ICU trial and thereby unfit to make such an assessment for the AID-ICU trial.<br>Data for days alive without coma or delirium to day 14 was extracted from the database.

**ATALAN 2013**	
**Methods**	Randomised clinical trial<br><br>The trial was conducted in a community hospital in Turkey (cardiac ICU)
**Participants**	**Sample size:** 53 (haloperidol 26; morphine 27)<br><br>**Age (mean):** 66 years<br><br>**Sex:** 26% females<br><br>**Baseline disease severity**: APACHEII score (preoperative) 6.01<br><br>**Co-morbidities:**<br>- cigarette use: haloperidol 46.2%; morphine 33.3%<br>- alcohol use: haloperidol 80.8%; morphine 66.7%<br>- chronic Obstructive Pulmonary Disease: haloperidol 7.7%; morphine 7.4%<br>- hypertension: haloperidol 61.5%; morphine 51.9%

	- noninsulin-dependent diabetes mellitus: haloperidol 34.6%; morphine 25.9%<br>- insulin-dependent diabetes mellitus: haloperidol 15.4%; morphine 14.8%<br>- previous stroke: haloperidol 7.7%; morphine 3.7%<br>- psychotropic medicine: haloperidol 11.5%; morphine 3.7%<br><br>**Setting:** Patients with hyperactive delirium after cardiac surgery admitted to ICU<br><br>**Inclusion criteria:** patients who had cardiac surgery with or without cardiopulmonary bypass<br><br>**Exclusion criteria:** patients who had a history of dementia and/or abnormal level of consciousness, Parkinson's disease, and recent seizures prior to surgery.<br><br>**Delirium screening:** CAM-ICU by research team. Motor subtype by RASS by clinicians (intensivist and psychiatrist). Assessed every 12h, until discharge from the hospital or for a maximum of 10 days. Patients were considered delirium free when they were free of symptoms for 24h.
**Interventions**	**Experimental:** 5mg haloperidol IM every hour until adequate RASS (-1 to +1), maximum 20mg.<br><br>**Control:** 5mg morphine sulfate IM every hour until adequate RASS (-1 to +1), maximum 20mg.<br><br>**Duration:** maximum 10 days<br><br>**Co-interventions:** If patients were still agitated when maximum dose was reached 2.5mg of lorazepam PO was added twice daily to the intervention.<br><br>During admission to the ICU, every patient was ventilated in assist-control mode to maintain pH between 7.35 and 7.45, PaCO2 between 35 and 45 mmHg, and PaO2 4 95%. Ventilation was weaned as per ICU protocol.<br><br>**Open-label antipsychotics:** Not reported
**Outcomes**	**Outcomes:**<br><br>- Duration time of delirious behaviour<br>- Daily total medication doses<br>- Need for additional sedative drug<br>- RASS scores<br>- The percentage of patients who maintained a RASS score within the target scores<br>- Reintubation<br>- Redo-surgery<br>- Length of ICU and hospital stay<br>- Readmission to the ICU<br>- Hospital mortality rate

	**Timing of outcomes:** until discharged from the hospital or for a maximum of 10 days following surgery.
**Funding**	Not reported
**Notes**	Email sent to Dr. Atalan 24 February 2023 and 13 March 2023. Reply received.

**BAKRI 2015**	
**Methods**	Randomised clinical trial<br><br>The trial was conducted in Saudi Arabia in a mixed ICU with trauma being the most common admission diagnosis<br><br>Study duration: 23 months
**Participants**	**Sample size:** 96 (haloperidol 32; dexmedetomidine 32; ondansetron 32)<br><br>**Age (mean):** 31 years<br><br>**Sex:** 9% females<br><br>**Baseline disease severity:** mean injury severity score (ISS): 24.5<br><br>**Co-morbidities:**<br>- Patients on mechanical ventilation: haloperidol 31.3%; dexmedetomidine 28.1%; ondansetron 21.8%<br><br>**Setting:** postoperative trauma patients admitted to the ICU<br><br>**Inclusion criteria:** adult postoperative trauma patients admitted to the ICU and screened positive for delirium with ICDSC.<br><br>**Exclusion criteria:** Patients were excluded if they had underlying neurological diseases, significant hearing loss, intracranial injury, ischemic/hemorrhagic strokes or language barrier that would confound the evaluation of delirium. Similarly, severely injured, deeply comatose, or moribund patients were excluded.<br><br>**Delirium screening:** Screening tool was ICDSC. Screening was performed by ICU nurses. Patients were screened twice daily for 3 days. The ICDSC was assessed 1h after study medication were given.
**Interventions**	**Experimental intervention**: 5mg haloperidol IV twice daily by infusion<br><br>**Control intervention**: dexmedetomidine 1μg/kg or 4mg ondansetron twice daily (infusion)<br><br>**Duration:** 3 days<br><br>**Open-label antipsychotics:** The treating physicians were free to prescribe additional haloperidol as rescue when clinically needed in all the three groups.

	Rescue haloperidol was used in 9.4% of the patients in the haloperidol group, 15.6% of the patients in the dexmedetomidine groups and in 34.4% of the patients in the ondansetron group. A total of 19.8% of the trial population was exposed to open-label antipsychotics.
**Outcomes**	**Outcomes:**<br><br>- Number of patients with delirium at day 3<br>- Number of patients requiring rescue haloperidol<br>- ICDSC scores<br>- Mean arterial blood pressure<br>- Mean Visual Analog Scale (VAS) of pain at the time of delirium assessment<br>- Serious adverse events<br>- Prolongation of QTc interval
**Founding sources**	No external funding
**Notes**	Email sent to Dr. Bakri asking for additional data 24 February and 13 March 2023. No reply was received.

**BREITBART-1996**	
**Methods**	Randomised clinical trial<br><br>The trial was conducted in the US<br><br>Study duration: not reported
**Participants**	**Sample size:** 32 (haloperidol 11, chlorpromazine 13, lorazepam 6)<br><br>**Age (mean):** 39.2<br><br>**Sex:** 23% female<br><br>**Co-morbidities:** not reported<br><br>**Setting:** AIDS patients admitted to high dependency AIDS unit<br><br>**Inclusion criteria:** Medically hospitalised patients with AIDS who was diagnosed with delirium according DSM-III-R criteria and had a score ≥ 13 on Delirium Rating Scale (DRS).<br><br>**Exclusion criteria:** known hypersensitivity to neuroleptics or benzodiazepines, presence of neuroleptic malignant syndrome, concurrent treatment with neuroleptic drugs, seizure disorder, current systemic chemotherapy for Karposi's sarcoma, withdrawal syndrome or anticholinergic delirium for which a more specific treatment was indicated, current or past diagnosis of schizophrenia, schizoaffective disorder, or bipolar disorder and the study compromising medical treatment for the underlying aetiology. Efforts were made to also exclude patients in whom delirium appeared to be part of a terminal event (i.e., the patient was expected to die within 24 hours).

	**Delirium screening:** enrolled patients were treated to the study protocol if they met DSM-III-R criteria for delirium and scored above the threshold score diagnostic for delirium (score of 13 or greater) on the delirium rating scale. The delirium rating scale is a 10 item scale specifically integrating DSM-III criteria. The maximum possible score is 32.
**Interventions**	**Experimental intervention:** a treatment protocol for study drug administration was followed. Each delirious patient was evaluated hourly with the Delirium Rating Scale - if the patient's score was 13 or greater, the next level dose of study drug was administered. After stabilization (when the patient was asleep, calm, and not hallucinating or had scored 12 or below on the Delirium Rating Scale) a maintenance dose was started on day 2 and continued for up to 6 days of treatment protocol. Mean haloperidol dose the first 24 hours was 2.8 mg. Average maintenance dose was 1.4 mg.<br><br>**Control intervention:** mean chlorpromazine dose the first 24 hours was 50 mg. Average maintenance dose was 36 mg. Mean lorazepam dose the first 24 hours was 3 mg. Average maintenance dose was 4.6 mg.<br><br>**Duration:** maximum 6 days<br><br>**Co-interventions:** not reported<br><br>**Open-label antipsychotics:** Not reported
**Outcomes**	- Mortality<br>- Scores on Delirium Rating Scale<br>- Scores on Mini-Mental State (cognitive function)<br>Timing of outcome measurement: day 2 end of treatment (delirium and cognitive scores) and 8 days from initiation of protocol (mortality)
**Funding sources**	Not reported
**Notes**	No information about use of rescue medication.<br><br>Midway through the study, lorazepam was removed from the study due to treatment limiting adverse side effects in this group.<br><br>28 May 2019: E-mail sent to Dr Breitbart asking for additional information on risk of bias and outcomes. Reply was received. Additional data on delirium resolution was not received.

**EuRIDICE-2022**	
**Methods**	Randomised clinical trial<br><br>The trial was conducted in the Netherlands, in 8 ICUs<br><br>Study duration: February 2018 to January 2020
**Participants**	**Sample size:** 142 (65 haloperidol, 67 placebo)

	**Age (mean):** 64 years<br><br>**Sex:** 32% females<br><br>**Baseline disease severity:** not available<br><br>**Co-morbidities:**<br>- Admission diagnosis: Respiratory (40%), Gastrointestinal (17%), Cardiovascular (18%), Transplant (8%), Trauma (4%), Other (9%)<br>- Mechanically ventilated 77%<br><br>**Setting:** Adult patients admitted to ICU<br><br>**Inclusion criteria for eligibility:** Adult patients admitted to the ICU<br><br>**Exclusion criteria for eligibility:** Admission to the ICU because of a primary acute neurological condition; pregnancy or breast-feeding; known allergy to haloperidol; history of ventricular arrhythmia (including torsade de pointes); neuroleptic malignant syndrome; parkinsonism; schizophrenia or other psychotic disorder; dementia or an Informant Questionnaire on Cognitive Decline in the Elderly (IQCODE) score ≥ 4; expected duration of ICU admission < 24 hours; inability to speak the Dutch language or to undergo a valid delirium assessment (e.g. deafness or blindness); or participation in another interventional trial. Additional exclusion criteria after development of delirium: QTc prolongation (QTc>500ms), acute alcohol (or substance) withdrawal syndrome, an expected ICU stay <24 hours, or torsade de pointes, neuroleptic malignant syndrome, or parkinsonism since ICU admission.<br><br>**Inclusion criteria for randomisation:** Delirium assessed with the Intensive Care Delirium Screening Checklist (ICDSC ≥4) or the Confusion Assessment Method for the ICU (positive CAM-ICU assessment), at the time of ICU admission or any ICU day after ICU admission. Written informed consent obtained from the patient or their legal representative. 3. All eligibility inclusion criteria (from above) are still met.<br><br>**Exclusion criteria for randomisation:** Prolonged QT-interval (QTc >500ms). (recent) Torsade de Pointes. (recent) Neuroleptic malignant syndrome or parkinsonism. Evidence of acute alcohol (or substance) withdrawal requiring pharmacological intervention (eg, benzodiazepines or alpha-2 agonist) to treat. Or if the patient is expected to die within 24hours or expected to leave the ICU within 24hours. No (previously) signed informed consent by patient or representative.<br><br>**Delirium screening:** CAM-ICU or ICDSC. Screening was performed by trained ICU nurses. Patients were evaluated 3 times daily. Patients were considered delirium free if delirium screenings were negative for 24 hours.
**Interventions**	**Experimental intervention:** 2.5mg haloperidol three times daily (patients aged ≥ 80 1mg), and increased up to 5mg three times daily (patients aged ≥ 80 2.5mg). Increased dose if delirium persisted in the next 8-hour shift. Decrease dose if

	negative 24 hours and stopped when another 24hours had elapsed. Trial drugs were re-initiated if delirium reoccurred.<br><br>**Control intervention:** Same as experimental intervention.<br><br>**Duration:** 14 days<br><br>**Open-label antipsychotics:** Use of open-label haloperidol was strongly discouraged, but could be used for acute breakthrough delirium symptoms that could not be managed in the proposed protocol. Atypical antipsychotics could be administered to patients with hallucinations. A total 13 patients (9.8%) received open-label haloperidol and 55 (41.6%) received other antipsychotics (quetiapine or olanzapine) open-label.
**Outcomes**	**Outcomes:**<br><br>Primary outcome<br>- number of delirium- and coma-free days (DCFDs) to day 14<br><br>Secondary outcomes:<br>- 28-day mortality<br>- duration of first delirium episode<br>- number of days with coma, agitation or mechanical ventilation<br>- mean daily RASS score<br>- use of “escape medication” for agitation or psychotic symptoms<br>- maximum mobility level<br>- sleep quality<br>- ICU length of stay<br>- adverse events related to delirium<br>- duration of the first delirium episode<br>- days with agitation<br>- serious adverse drug associated events<br>- Time to ‘readiness for discharge from the ICU’.<br>- Hospital discharge
**Notes**	The trial was pre-maturely stopped.
**Funding**	A grant has been provided by ZonMw – The Netherlands Organisation for Health Research and Organisation. ZonMw project number: 848041001
**Notes**	Unpublished data of the EuRIDICE trial on mortality and SAEs/SARs were included in this review. Data on Days alive without delirium or coma and QTc prolongation were still privileged until publication of the trial results and thereby not included in this review. Email sent to Dr. van der Jagt and L. Smith on 24 February asking for additional data, reply was received.

**GARG-2022**	
**Methods**	Randomised clinical trial<br><br>The trial was conducted in India

	Study duration: February 2021 to February 2022
**Participants**	**Sample size:** 45 (15 haloperidol, 15 Quetiapin, 15 Placebo)<br><br>**Age (mean):** 57.5<br><br>**Sex:** 44.4% females<br><br>**Baseline disease severity:** APACHEII score 29.5<br><br>**Co-morbidities:** Not reported.<br><br>**Setting:** Adult patients in the ICU<br><br>**Inclusion:** Adult patients admitted to the ICU with delirium and accepting enteral medication.<br><br>**Exclusion criteria:** Patients who at baseline had severe cognitive impairment, one who were at high risk for medication side-effects because of pregnancy and breast feeding. Patients with history of torsades de pointes, neuroleptic malignant syndrome, or allergy to haloperidol or quetiapine were not considered. Also those who had ongoing treatment with antipsychotics, having rapidly resolving organ failure, moribund patients, and those who were blind or unable to speak or understand were excluded<br><br>**Delirium screening:** CAM-ICU. No information about who performed screening
**Interventions**	**Experimental intervention:** Oral haloperidol max 30mg daily, halved or doubled every 12 h titrated according to CAM-ICU and evaluation of side-effects of the intervention.<br><br>**Control intervention:** Matching placebo or quetiapine (max 300mg)<br><br>**Open-label antipsychotics:** not reported<br><br>**Duration:** Unclear, but all outcomes are reported at 14 days. It is anticipated that this is the intervention period and patients received the intervention as long as they were delirious.<br><br>**Co-intervention:** not reported
**Outcomes**	**Outcomes:**<br>- Number of days alive without delirium during the 14 day intervention period<br>- Time to freedom of mechanical ventilation<br>- Time to ICU discharge<br>- Time to Hospital discharge<br>- Mortality (30 day and 90 day)
**Funding**	Financial or Other Competing Interests: None

**Notes**	Email sent to Dr. Kumar Singh on 24 February and March 2023 asking for additional data. No reply was received.

**GIRARD-2018**	
**Methods**	Randomised clinical trial<br><br>The trial was conducted in the USA,<br><br>Study duration: December 2011 to August 2017
**Participants**	**Sample size:** 566 (haloperidol 192, ziprasidone 190, placebo 184)<br><br>**Age (median):** 60 years<br><br>**Sex:** 57% males<br><br>**Baseline disease severity:** median APACHE II score at ICU admission: 28.8; Median SOFA score at randomisation: 11; Informant Questionnaire on Cognitive Decline in the Elderly (Short IQCODE): 3.1<br><br>**Co-morbidities:**<br>Median Charlson Comorbidity Index score: 2<br>Received assisted ventilation before randomisation: invasive 93%; non-invasive 3%<br>Shock before randomisation: 33%<br><br>**Diagnosis at admission:**<br>- Adult respiratory distress syndrome: placebo 21%; haloperidol 23%; ziprazidone 18%<br>- Sepsis: placebo 19%; haloperidol 22%; ziprazidone 17%<br>- Airway protection: placebo 29%; haloperidol 24%; ziprazidone 23%<br>- Chronic obstructive pulmonary disease, asthma, or other pulmonary disorder: placebo 12%; haloperidol 10%; ziprazidone 15%<br>- Surgery: placebo 7%; haloperidol 7%; ziprazidone 12%<br>- Chronic heart failure, myocardial infarction, or arrhythmia: placebo 3%; haloperidol 3%; ziprazidone 3%<br>- Cirrhosis or liver failure: placebo 3%; haloperidol 2%; ziprazidone 2%<br>- Seizures or neurologic disease: placebo 1%; haloperidol 2; ziprazidone 1%<br><br>**Setting:** Patients admitted to the ICU (28% admitted to surgical ICU).<br><br>**Inclusion criteria:** adults (≥18 years old) with delirium admitted to medical and/or surgical ICUs in participating hospitals who were treated with mechanical ventilation, non-invasive positive pressure ventilation, vasopressor(s), or intraaortic balloon pump.<br><br>**Exclusion criteria:** patients who, at baseline, had severe cognitive impairment; were at high risk for medication side effects because of pregnancy, breast-feeding, a history of torsades de pointes, QT prolongation, a history of neuroleptic malignant syndrome, or allergy to haloperidol or ziprasidone; were receiving

	ongoing treatment with an antipsychotic medication; were in a moribund state; had rapidly resolving organ failure; were blind, deaf, or unable to speak or understand English; were incarcerated; or were enrolled in another study or trial that prohibited co-enrolment.<br><br>**Delirium:** Delirium was detected with the use of the Confusion Assessment Method for the ICU (CAM-ICU). Trained research personnel evaluated patients twice daily until delirium was present or until death, discharge from the ICU, development of an exclusion criterion, or a maximum of 5 days.<br><br>Delirium characteristics at randomisation: 11% hyperactive and 89% hypoactive.
**Interventions**	**Experimental intervention:** patients younger than 70 years of age received 2.5 mg haloperidol per 0.5 ml and 1.25 mg of haloperidol per 0.25 ml when older than 70 years of age. Patients in the haloperidol group received a dose of up to 10 mg per administration and up to 20 mg per day. Mean (±SD) daily doses of haloperidol administered were 11.0±4.8 mg.<br><br>**Control intervention:** patients younger than 70 years of age received 5 mg of ziprasidone per 0.5 ml or 2.5 mg of ziprasidone per 0.25 ml when older than 70 years of age. Patients in the ziprasidone group received a dose of up to 20 mg per administration and up to 40 mg per day. Mean (±SD) daily doses of ziprasidone administered were 20.0±9.4 mg.<br><br>Patients younger than 70 years of age received 0.5 ml placebo (0.9% saline) and 0.25 ml of placebo when older than 70 years of age.<br><br>Volume and dose of a trial drug or placebo were halved if a patient did not have delirium (i.e., had a negative CAM-ICU assessment) for two consecutive assessments and was not yet receiving the minimum dose. Trial drug or placebo were temporarily withheld if a patient did not have delirium for four consecutive assessments or for safety reasons.<br><br>Trial drug or placebo were permanently discontinued when any of the following occurred: torsades de pointes, neuroleptic malignant syndrome, drug reaction with eosinophilia and systemic symptoms syndrome, new onset coma due to structural brain disease, or any life-threatening, serious adverse event that was related to the intervention, as determined by an independent data and safety monitoring board.<br><br>**Timing:** patients were randomised when delirium was present at the time of informed consent or during the 5 days after informed consent was obtained and the corrected QT interval was less than 550 msec on a 12-lead electrocardiogram. Immediately after the trial-group assignment, the first dose of trial drug or placebo was administered.<br><br>**Duration:** 14 days or at ICU discharge. The median duration of exposure to a trial drug or placebo was 4 days (interquartile range, 3 to 7).

	**Co-intervention:** Approximately 90% of the patients received analgesics or sedatives. Daily rate of adherence to each of the five components of the ABCDE bundle was greater than 88%<br><br>**Use of open-label antipsychotics:** Administration of open-label haloperidol, ziprasidone, or any other antipsychotic (except those prescribed specifically for nausea, such as compazine) was restricted during the 14-day study drug period. After the 14-day study drug period, open-label antipsychotics could be administered for refractory delirium according to the ICU team's preference. 118 patients (21 %) of the trial population received open-label antipsychotics during the trial.
**Outcomes**	**Outcomes:**<br>- days alive without delirium or coma (defined as the number of days that a patient was alive and free from both delirium and coma during the 14-day intervention period)<br>- duration of delirium<br>- time to freedom from mechanical ventilation (defined as extubation that was followed by at least a 48- hour period during which the patient was alive and free from mechanical ventilation)<br>- time to final successful ICU discharge (defined as the last ICU discharge during the index hospitalization that was followed by at least a 48-hour period during which the patient was alive and outside the ICU)<br>- time to ICU readmission<br>- time to successful hospital discharge (defined as discharge that was followed by at least a 48-hour period during which the patient was alive and outside the hospital)<br>- 30-day and 90-day survival<br>- incidence of torsades de pointes<br>- incidence of neuroleptic malignant syndrome<br>- severity of extrapyramidal symptoms as measured on the modified Simpson–Angus Scale<br><br>**Timing of outcome measurement:** outcome dependent
**Notes**	29 May 2019: E-mail sent to Dr Ely and Dr Girard asking for additional information on the outcomes. Reply was received. Link to complete dataset was received and number of events of prolonged QTc was extracted. Cognitive function was only measured as pre-existing cognitive impairment.
**Funding**	Supported by grants (AG035117 and TR000445) from the National Institutes of Health and by the Department of Veterans Affairs Geriatric Research Education and Clinical Center.

**GRAHAM-2006**	
**Methods**	Pilot randomised, non-blinded clinical trial<br>The trial was conducted in US (Denver Health Medical Center)<br><br>Study duration: not reported
**Participants**	**Sample size:** 10 (4 haloperidol, 6 control)

	**Age (mean):** 58 years<br><br>**Sex:** 20% female<br><br>**Co-morbidities:** Not reported<br><br>**Setting:** Adult patients admitted to an ICU<br><br>**Inclusion:** Intubated adult patients admitted to an ICU<br><br>**Exclusion criteria:** Patients were excluded if they had a psychiatric diagnosis, liver failure, and inability to consent<br><br>**Delirium screening:** CAM-ICU, no information about who performed screening nor how often they were assessed for delirium
**Interventions**	**Experimental intervention:** Haloperidol titrated to max 80 mg/d<br><br>**Control intervention:** No control<br><br>**Co-intervention:** Patients were sedated with a standard of care sedation protocol based on fentanyl and midazolam with or without titratable haloperidol dependent on assigned group.
**Outcomes**	**Outcomes:**<br>- Duration of intubation<br>- Duration of ICU stay<br>- Mini-mental scores<br>- Mortality<br>- Number of SAEs (self-extubation, qtc prolongation)
**Notes**	Only abstract. Dr. Graham was contacted by email, reply was received no additional data available.
**Funding**	No information

**HAN-2004**	
**Methods**	Randomised, clinical trial<br>The trial was conducted in Korea<br><br>Study duration: not reported
**Participants**	**Sample size:** 28 (12 haloperidol, 12 risperidone)<br><br>**Age (mean):** 66 years<br><br>**Sex:** 47% female<br><br>**Co-morbidities:**<br>- Fractures 29%<br>- Cerbrovascular accident 21%<br>- Chronic renal failure 1%<br>- Cancer 8%

	- Cardiovascular disease 13%<br><br>**Baseline disease severity:** mean Memorial Delirium Assessment Scale score 24.5<br><br>**Setting:** Adult patients admitted to 2 ICUs, 4 medical wards and 2 oncology wards<br><br>**Inclusion:** Adult patients with delirium according to DSM-III-R criteria.<br><br>**Exclusion criteria:** Patients with any type of dementia or other psychiatric diagnosis determined with the SCID were excluded. Patients who had already been injected with antipsychotics or benzodiazepines in the emergency room or intensive care unit for their disturbing behavioral problems before the arrival of the consulting psychiatrist were excluded. Patients with inability to speak were excluded.<br><br>**Delirium screening:** Delirium Rating Scale with cut-off of 13.
**Interventions**	**Experimental intervention:** Haloperidol 0.75mg twice daily, increasing dose according to status of delirium during 7 days. Mean dose during 7 days was 1.71mg (0.84).<br><br>**Control intervention:** Risperidone 0.5mg twice daily, increasing dose according to status of delirium during 7 days. Mean dose during 7 was 1.02mg (0.41).<br><br>**Duration:** 7 days
**Outcomes**	**Outcomes:**<br>- mean delirium Rating Scale scores<br>- delirium severity assessed with Memorial Delirium Assessment Scale<br>- delirium resolution<br>- duration of delirium
**Notes**	The study included patients admitted to four medical wards, two ICUs and two oncology wards. As more than 90% of the included patients came from ICU, we have included this study in our review.<br><br>Memorial Delirium Assessment Scale scores (mean and standard deviation) were extracted from figure 1.<br><br>29 May 2019: E-mail sent to Dr Kim asking for additional information on risk of bias and outcomes. Reply was received. No additional data or clarifications were received due to loss of data
**Funding**	Dr. Kim is supported by the Brain Korea 21 Project of the Ministry of Education and Human Resources Development, Republic of Korea.

**ORIC-I**	
**Methods**	Randomised, clinical trial<br>The trial was conducted in USA<br><br>Study duration: March 2006 to October 2011

**Participants**	**Sample size:** 30 (16 haloperidol, 14 risperidone)<br><br>**Age (mean):** not reported<br><br>**Sex:** 37% female<br><br>**Co-morbidities:** Not reported<br><br>**Baseline disease severity:** Not reported<br><br>**Setting:** Adult, mechanically ventilated patients admitted to ICU<br><br>**Inclusion:** all adult (>=18 years of age) mechanically ventilated patients admitted to the medical, surgical, trauma or cardiothoracic ICUs who are expected by the ICU clinical team to require > 24 hours of mechanical ventilation<br><br>**Exclusion criteria:** Baseline QTc >480 milliseconds (ms); history of Parkinsons's disease; pregnancy; history of schizophrenia or neurologic disease that could confound the delirium assessment; deafness or inability to understand English or Spanish; extubation prior to enrolment; previously enrolled in the same study; patient, family, or attending physician refusal; death before enrolment; treatment with haloperidol within 2 days prior to ICU admission; and prisoners.<br><br>**Delirium screening:** Not reported
**Interventions**	**Experimental intervention:** haloperidol 5 mg IV q 12h, which was continued until liberation from mechanical ventilation or 28 days<br><br>**Control intervention:** Placebo, identical to experimental<br><br>**Duration:** to liberation of mechanical ventilation or max 28 days
**Outcomes**	**Outcomes:**<br>- 28 day mortality<br>- 90 day mortality<br>- duration of mechanical ventilation<br>- ICU length of stay<br>- Total delirium days<br>- Adverse events
**Notes**	The study was identified when searching clinical.trials.gov. The study was terminated early due to insufficient recruitment to meet the aims. The study has not been published, but some results have been posted at clinical.trials.gov.<br>3 June 2019: E-mail sent to Barbara Early asking for additional information on risk of bias and outcomes. Reply was received. It was clarified that mortality was measured at day 30 and that the 4 measurements of QTc prolongation corresponds to 4 individual patients. Rescue drug was not used. No additional results were available and no clarifications for the risk of bias assessment was received.
**Funding**	NIH Grant, project number 1K23HL078760-01A1

TALEBI-2021	
**Methods**	Randomised, clinical trial<br>The trial was conducted in Iran<br><br>Study duration: March 2017 to 2018
**Participants**	**Sample size:** 200 (100 haloperidol, 100 quetiapin)<br><br>**Age (mean):** 60<br><br>**Sex:** 37,5% female<br><br>**Co-morbidities:**<br>- Stroke (haloperidol 4%, quetiapine 2.5%)<br>- Metabolic (haloperidol 14.5%, quetiapine 19%)<br>- Infectious (haloperidol 25.5%, quetiapine 26%)<br>- Cardiovascular (haloperidol 6%, quetiapine 5.5%)<br><br>**Baseline disease severity:** Not reported<br><br>**Setting:** Adult patients admitted to emergency department or ICU<br><br>**Inclusion:** Patients aged 18 and over whose delirium condition was confirmed based on the Diagnostic and Statistical Manual of Mental Disorders, 5th edition, and psychiatric consulting.<br><br>**Exclusion criteria:** patients with dementia, poisoning, alcohol and benzodiazepines deprivation, visual, and hearing impairment, lack of response to verbal or physical stimuli, history of long QTc (>500 ms), history of medicine allergy, neuroleptic malignant syndrome, Parkinson's, mental diseases, and mood disorders were excluded from the study.<br><br>**Delirium screening:** DSM-5
**Interventions**	**Experimental intervention:** haloperidol 5 mg intramuscular injection q 12h, adjusted according to clinical evaluation.<br><br>**Control intervention:** quetiapine 25 mg orally daily, adjusted according to clinical evaluation.<br><br>**Duration:** Not reported<br><br>**Open-label antipsychotics:** Not reported
**Outcomes**	**Outcomes:**<br>- Delirium Severity with Delirium Rating Scale-Revised-98
**Funding**	This study was funded by Mashhad University of Medical Sciences.
**Notes**	Email sent to Dr. Talebi asking for additional data on the 24. February 2023, reply was received.

Selected excluded trials

Wrong intervention

1. Adams F, Fernandez F, Andersson BS, Emergency pharmacotherapy of delirium in the critically ill cancer patient, Psychosomatics 1986;27(1 Suppl):33-8
2. Davies MP, Effect of Lorazepam with Haloperidol vs. Haloperidol Alone on Agitated Delirium in Patients, Journalof Pain and Symptom Management 2018;55(2):546-546
3. Goldstein BJ, Clyde DJ, Haloperidol in controlling symptoms of acute psychoses. A double-blind evaluation of haloperidol and trifluoperazine, Current Therapeutic Research-Clinical and Experimental 1966;8(5):236.
4. Hui D, Frisbee-Hume S, Wilson A, Dibaj SS, Nguyen TT, De La Cruz MG, et al, Lorazepam as an adjuvant to haloperidol for agitated delirium at the end-of-life: A double-blind randomized controlled trial, Supportive care in cancer 2017;25 (2 Supplement 1)():S192-S193
5. Jain R, Arun P, Sidana A, Sachdev A, Comparison of efficacy of haloperidol and olanzapine in the treatment of delirium, Indian journal of psychiatry 2017;59(4):451-456
6. Jin KJ, Kook LH, Un PC, Uk LC, Ho PI, Chul L, Comparison of intramuscular olanzapine and haloperidol for thetreatment of delirium, European Psychiatry 2009;24:1
7. Jung HY, Lee SI, Kim SG, Hong JH, Double blind comparison on the efficacy of haloperidol versus quetiapine in the treatment of delirium, International Journal of Neuropsychopharmacology 2008;11():299-299
8. Jung W, Jung HY, Lee SI, Kim SG, Shin EY, Park J, Park M, Comparison on the efficacy of risperidone versus haloperidol in the treatment of delirium: prospective, randomized, double blind trial, 163rd annual meeting of the american psychiatric association 2010
9. Kim JJ, Lim HK, Pae CU, Lee CU, Lee C, Paik IH, Comparison of intramuscular olanzapine and haloperidol for the treatment of delirium, European neuropsychopharmacology 2008;18 (S4):S424-S425
10. Kim YR, Jung HY, Lee ISY, Kwon YJ, Cho SH, Hong JH, Double blind trial of amantadine and haloperidol for treatment of delirium, European neuropsychopharmacology 2006;16:S548-S548
11. Kumar V, Chakrabarti S, Grover S, Comparative efficacy of typical and atypical antipsychotic in delirium Indian journal of psychiatry 2010;1:S22
12. Lee CS, Rim GM, Hahn KH, Kim BI, Comparison of Efficacy between Aripiprazole and Haloperidol in the Treatment of Patients with Delirium, Korean journal of psychopharmacology 2007;18(4):240-245
13. Lee KJ, Kim H, Lee SH, Chung YC, Effects of risperidone in delirium: A comparison with haloperidol International Journal of Neuropsychopharmacology 2004;7:S384-S385
14. Lee SH, Park SJ, Comparison of efficacy and side effects between aripiprazole and haloperidol in the treatment of delirium, International psychogeriatrics 2013;1:S132-S133
15. Lee Y, Lee J, Rim HD, Kim SH, Chung US, Cho GA, Won SH, A comparative study of haloperidol and quetiapine in the treatment of delirium: a preliminary randomized open label, flexible dose trial, European neuropsychopharmacology 2006;16:S488-S489
16. Lee YI, Jung HY, Lee SI, Kim SG, Park JH, Comparison on the Efficacy of Quetiapine Versus Haloperidol in the Treatment of Delirium: prospective, Randomized Trial, Korean journal of biological psychiatry 2009;16(1):15-24
17. Lim HK, Paik IH; Oh K, Lee CU, Lee C, Comparison of the Clinical Efficacy and Safety between Intramuscular Olanzapine and Intramuscular Haloperidol Injection in the Treatment of Delirium, Korean journal of psychopharmacology 2007;18(6):423-428

18. Maneeton B, Maneeton N, Srisurapanont M, A double-blind, randomised, controlled trial of quetiapine versushaloperidol for the treatment of delirium: A preliminary report, European neuropsychopharmacology September 2011;3:S557
19. Maneeton B, Maneeton N, Srisurapanont M, Chittawatanarat K, Quetiapine versus haloperidol in the treatment of delirium: a double-blind, randomized, controlled trial, Drug design, development and therapy 2013;7():657-667
20. Pasechnik IN, Makhlai AV, Tepliakova AN, Gubaidullin RR, Sal'nikov PS, Borisov A, Berezenko MN, New approach to postoperative delirium treatment. [Russian], Khirurgiia 2015;(3):71-75
21. Peterson LG, Bongar B, Navane versus Haldol, Treatment of acute organic mental syndromes in the general hospital, General hospital psychiatry 1989;11(6):412-417
22. Ropert R, Payan C, Allard S, Sultopride versus haloperidol for the treatment of acute psychosis. Results of a multicenter double-blind controlled trial, Annales de psychiatrie 1989;4(1):92-98
23. Terhaar HW, Comparison of chlormethiazole and haloperidol in treatment of elderly patients with confusion or organic and psychogenic origin – a double-blind crossover study, Pharmatherapeutica 1977;1(9):563-569
24. Tune L, Jewart R, Egeli S, Greene Y, Pharmacologic management of acute delirium: A naturalistic, prospective comparison of atypical antipsychotics and haloperidol, Journal of the american geriatrics society 2001;49(4):S93-S93

Wrong indication

1. Eremenko AA; Chernova EV, Dexmedetomidine use for intravenous sedation and delirium treatment during early postoperative period in cardio-surgical patients, Anesteziologiia i reanimatologiia 2013;(5):4-8
2. Girard TD, Pandharipande PP, Carson SS, Schmidt GA, Wright PE, Canonico AE, Pun BT, Thompson JL, Shintani AK, Meltzer HY, et al, Feasibility, efficacy, and safety of antipsychotics for intensive care unit delirium: the MIND randomized, placebo-controlled trial, Critical care medicine 2010;38(2):428-437
3. Page VJ, Ely EW, Gates S, Zhao XB, Alce T, Shintani A, Jackson J, Perkins GD, McAuley DF, Effect of intravenous haloperidol on the duration of delirium and coma in critically ill patients (Hope-ICU): a randomised, double-blind, placebo-controlled trial, The Lancet Respiratory medicine 2013;1(7):515-523
4. Page VJ, Marti J, McAuley DF, Casarin A, Alce, T, Zhao XB, Health evaluation and cost-effectiveness analysis from a randomized trial of haloperidol in the management of delirium in the critically ill (Hope-ICU Trial), American journal of respiratory and critical care medicine 2015;191:A4022
5. Litvineneko IV, Odinak MM, Khlystov LUV, Perstnev SV, Fedorov BB, Efficacy and safety of rivastigmine (exelon) in the confusion syndrome in the acute phase of ischemic stroke, Zhurnal nevrologii i psikhiatrii imeni S.S. Korsakova 2010;110(11 Pt 2):36-41

Wrong intervention

1. Eremenko, Aa; Chernova, Ev, Treatment of delirium in the early postoperative period after cardiac surgery, Anesteziologiia i reanimatologiia 2014;(3):30-34
2. Khan BA, Perkins AJ, Campbell NL, Gao S, Farber MO, Wang S, Khan SH, Zarzaur BL, Boustani MA, Pharmacological Management of Delirium in the Intensive Care Unit: A Randomized Pragmatic Clinical Trial, Journal of the american geriatrics society 2019; 67(5):1057–65.
3. van Eijk MM, Roes KC, Honing ML, Kuiper MA, Karakus A, van der Jagt M, Spronk PE, van Gool WA, van der Mast RC, Kesecioglu J, Slooter AJ, Effect of rivastigmine as an adjunct to usual care with haloperidol on duration of delirium and mortality in critically ill patients: a multicentre, double-blind, placebo-controlled randomised trial, Lancet 2010;376:1829-37

Ongoing and terminated trials

Trial ID	Trial name	Country	Status	Registration year	Target sample size	Experimental intervention	Control
IRCT20141209020258N114	Comparison the effect of Olanzapine and Haloperidol on the treatment of delirium in ICU in Valiasr hospital	Iran	Recruiting	2019	50	Haloperidol	Olanzapine
IRCT20180911040998N1	Comparison the Effect of Quetiapine and Haloperidol on the Treatment of delirium in ICU	Iran	Recruiting	2019	60	Haloperidol	Quetiapin
IRCT20200927048852N1	Comparison of the effect of Olanzapine and Haloperidol and quetiapine on the Treatment of delirium in Intensive care unit (ICU) patients	Iran	Recruiting	2020	90	Haloperidol	Quetiapin and Olanzapine
JPRN-jRCT1051220015	Dexmedetomidine versus Haloperidol for Sedation of Non-intubated Patients with Hyperactive Delirium during the Night in the High Dependency Unit: a single-center, prospective, randomized, open-label controlled trial - DEX-HD trial	Japan	Recruiting	2022	100	Haloperidol	Dexmedetomidine

NCT00599287	Methylphenidate, rivastigmine or haloperidol in hypoactive delirious intensive care patients: a single centre, randomized, mono-blind pilot trial - Methylphenidate, rivastigmine or haloperidol in hypoactive delirious ICU-patients	Netherlands	Terminated. No data. Incl rate too low due to a lack of eligible patients and difficulties obtaining informed consent	2008	80	Haloperidol	Methylphenidate and Rivastigmin
NCT00833300	Haloperidol vs Olanzapine for the Management of ICU Delirium	Canada	Terminated	2009	200	Haloperidol	Olanzapine
NCT01140529	Dexmedetomidine for the Treatment of Delirium After Heart Surgery (DexinDelir)	Sweden	Terminated, slow recruitment	2010	3 enrolled (terminated)	Haloperidol	Dexmedetomidine or placebo
NCT02343575	Valproic Acid for Treatment of Hyperactive or Mixed Delirium in ICU	US	Terminated	2015	3 enrolled (none to haloperidol)	Haloperidol	Placebo or Valproic acid
NCT02345902	Treatment of Hypoactive Delirium and Outcome Measures (THDOM)	Mexico	Unknown, was recruiting	2015	60	Haloperidol	Placebo or non-pharm

Risk of bias assessments of outcomes

**Study ID**	**Experimental**	**Comparator**	**Outcome**	**D1**	**D2**	**D3**	**D4**	**D5**	**Overall**
AID-ICU	Haloperidol	Placebo	Mortality	+	+	+	+	+	+
AID-ICU	Haloperidol	Placebo	SAE/SARs	+	+	+	+	+	+
AID-ICU	Haloperidol	Placebo	Days alive without delirium or coma	+	+	+	+	+	+
AID-ICU	Haloperidol	Placebo	QTc prolongation	+	+	+	+	+	+
ATALAN-2013	haloperidol	morphine	Mortality	-	!	+	+	!	-
ATALAN-2013	haloperidol	morphine	SAR/SAES	-	!	+	-	!	-
BAKRI 2015	haloperidol	Dexmed Ondansetrone	SAR/SAEs	!	+	+	-	!	-
BAKRI 2015	haloperidol	Dexmed Ondansetrone	Delirium severity	!	+	+	+	!	!
BAKRI 2015	haloperidol	Dexmed Ondansetrone	QTc prolongation	!	!	+	+	!	-
BREITBART-1996	haloperidol	chlorpromazine	mortality	!	!	+	+	!	-
BREITBART-1996	haloperidol	chlorpromazine	SAE/SARs	!	!	+	+	!	-
BREITBART-1996	haloperidol	chlorpromazine	Delirium severity	!	!	+	-	!	-
BREITBART-1996	haloperidol	chlorpromazine	Cognitive function	!	!	+	-	!	-
BREITBART-1996	haloperidol	chlorpromazine	QTc prolongation	!	!	+	-	!	-

+	Low risk
!	Some concerns
-	High risk
D1	Randomisation process
D2	Deviations from the intended interventions
D3	Missing outcome data
D4	Measurement of the outcome
D5	Selection of the reported result

EuRIDICE	haloperidol	placebo	Mortality	+	+	+	+	+	+		
EuRIDICE	haloperidol	placebo	SAE/SARs	+	+	+	+	!	!		
EuRIDICE	haloperidol	placebo	Days alive without delirium or coma	+	+	+	+	+	+		
EuRIDICE	haloperidol	placebo	QTc prolongation	+	+	+	+	+	+		
CARG-2022	haloperidol	quetiapin placebo	mortality	!	!	+	+	!	-		
CARG-2022	haloperidol	Quetiapin placebo	SAE/SARs	!	!	+	-	!	-		
CARG-2022	haloperidol	placebo quetiapine	Days alive without delirium	!	!	+	-	!	-		
Girard-2018	Haloperidol	Placebo ziprasidone	Mortality	+	+	+	+	+	+		
Girard-2018	Haloperidol	Placebo ziprasidone	SAE/SARs	+	+	+	+	+	+		
Girard-2018	Haloperidol	Placebo ziprasidone	Delirium duration	+	+	+	+	+	+		
Girard-2018	Haloperidol	Placebo ziprasidone	QTc prolongation	+	+	+	+	+	+		
GRAHAM-2006	Haloperidol	No control	Mortality	!	!	+	+	!	-		
GRAHAM-2006	haloperidol	No control	SAE/SARs	!	!	+	-	!	-		
GRAHAM-2006	haloperidol	No control	Cognitive function	!	!	+	-	!	-		
GRAHAM-2006	haloperidol	No control	QTc prolongation	!	!	+	-	!	-		
HAN-2004	Haloperidol	Risperidone	SAE/SARs	!	!	+	-	!	-		
HAN-2004	Haloperidol	Risperidone	Delirium duration	!	!	+	+	!	-		

ORIC-2017	haloperidol	placebo	Mortality	!	!	+	+	!	-		
ORIC-2017	haloperidol	placebo	SAE/SARs	!	!	+	-	!	-		
ORIC-2017	haloperidol	placebo	QTc prolongation	!	!	+	-	!	-		
TALEBI-2022	haloperidol	quetiapine	delirium severity	+	!	+	-	!	-		

Table S1: GRADE summary of findings for haloperidol versus other antipsychotics

Certainty assessment							№ of patients		Effect		Certainty	Importance
**№ of studies**	**Study design**	**Risk of bias**	**Inconsistency**	**Indirectness**	**Imprecision**	**Other considerations**	**Haloperidol**	**Other Antipsychotics**	**Relative (96.7%/98% CI)**	**Absolute (96.7%/98% CI)**		
**Mortality (follow-up: range 15 days to 90 days)**												
3	randomised trials	not serious[a]	not serious	serious[b]	very serious[c]	none[d]	81/218 (37.2%)	76/218 (34.9%)	**RR 1.07** (0.82 to 1.41)	**24 more per 1.000** (from 63 fewer to 143 more)	⊕◯◯◯ Very low	
**SAE/SAR (highest proportion) (follow-up: range 7 days to 90 days)**												
4	randomised trials	not serious[a]	not serious	serious[e]	very serious[c]	none[d]	81/230 (35.2%)	76/230 (33.0%)	**RR 1.06** (0.81 to 1.40)	**20 more per 1.000** (from 63 fewer to 132 more)	⊕◯◯◯ Very low	
**SAEs/SARs (cummulated) (follow-up: range 7 days to 90 days)**												
4	randomised trials	not serious[a]	not serious	serious[e]	very serious[c]	none[d]	154/230 (67.0%)	146/230 (63.5%)	**RR 1.05** (0.93 to 1.18)	**32 more per 1.000** (from 44 fewer to 114 more)	⊕◯◯◯ Very low	
**Days Alive without Delirium or Coma (14 days)**												

Certainty assessment							№ of patients		Effect		Certainty	Importance
**№ of studies**	**Study design**	**Risk of bias**	**Inconsistency**	**Indirectness**	**Imprecision**	**Other considerations**	**Haloperidol**	**Other Antipsychotics**	**Relative (96.7%/98% CI)**	**Absolute (96.7%/98% CI)**		
2	randomised trials	not serious[a]	not serious	serious[f]	serious[c]	none[d]			-	MD **0.15 days lower** (1.83 lower to 1.53 higher)	⊕⊕◯◯ Low	
**Severity (follow-up: range 3 days to 7 days; assessed with: DRS/MDAS/DRS-98)**												
3	randomised trials	serious[g]	not serious	serious[h]	serious[i]	none[d]			-	SMD **0.04 SD higher** (0.26 lower to 0.33 higher)	⊕◯◯◯ Very low	
**Cognitive Function (follow-up: mean 6 days; assessed with: MMSE; Scale from: 0 to 30)**												
1	randomised trials	serious[j]	not serious	not serious	very serious[k]	none[d]			-	MD **2.1 points higher** (8.72 lower to 12.92 higher)	⊕◯◯◯ Very low	
**Health-related quality of life - not reported**												
-	-	-	-	-	-	-	-	-	-	-	-	
**QTc-prolongation (follow-up: mean 14 days)**												

Certainty assessment							№ of patients		Effect		Certainty	Importance
**№ of studies**	**Study design**	**Risk of bias**	**Inconsistency**	**Indirectness**	**Imprecision**	**Other considerations**	**Haloperidol**	**Other Antipsychotics**	**Relative (96.7%/98% CI)**	**Absolute (96.7%/98% CI)**		
1	randomised trials	not serious	not serious	not serious	very serious[l]	none[d]	13/192 (6.8%)	20/190 (10.5%)	**RR 0.64** (0.31 to 1.33)	**38 fewer per 1.000** (from 73 fewer to 35 more)	⊕⊕◯◯ Low	

CI: confidence interval; **MD:** mean difference; **RR:** risk ratio; **SMD:** standardised mean difference

* TSA-adjusted 96.7% CI for primary outcomes and QTc prolongation and TSA-adjusted 98% CI for secondary outcomes.

Explanations

a. 1 trials was of low risk of bias. This trial contributed with the majority of patients

b. All trials had different antipsyhotics as comparator these may differ in intervention effect

c. Less than 50% of the required information sized accrued as estimated with TSA. The cumulative z-curve did not cross any monitoring boundaries. Therefore, downgraded 2 levels.

d. 2 trials identified in trial registers which were either terminated, completed or status unknown and trial results were not available. These unpublished trials were not judged as serious publication bias.

e. Different comparators (chlorpormazine, quetipaine, ziprasidone, risperidone)

f. Different comparators (quetiapin, ziprasidone)

g. All trials had high risk of bias

h. Different comparators (chlorpromazine, risperidone, quetiapine) and different tools used for measurement of severity

i. wide 98% CI: -0.26 to 0.33 indicating high uncertainty. Could not perform TSA on SMD

j. 1 trial which was adjundicated high risk of bias

k. Not possible to conduct TSA due to too little information. Downgraded by 2.

l. Could not perform TSA due to too little information, downgraded by 2.

Figure S1: All-cause mortality; haloperidol versus all comparators

Study or Subgroup	Year	Risk of Bias	Haloperidol Events	Haloperidol Total	Control Events	Control Total	RR	96.7% CI	Weight (fixed)	Weight (random)
**Placebo**										
Andersen–Ranberg et al. (placebo)	2022	Low	182	501	210	485	0.84	[0.71; 0.99]	71.2%	70.9%
EuRIDICE (placebo)	unpubl.	Low	10	65	13	67	0.79	[0.35; 1.79]	4.3%	3.0%
Garg et al. (placebo)	2022	High	6	15	7	15	0.86	[0.35; 2.10]	2.3%	2.5%
Girard et al. (placebo)	2018	Low	73	192	63	184	1.11	[0.83; 1.49]	21.5%	23.2%
ORIC–1 (placebo)	2017	High	1	16	2	13	0.41	[0.03; 4.88]	0.7%	0.3%
**Total (fixed effect, 96.7% CI)**			**272**	**789**	**295**	**764**	**0.89**	**[0.77; 1.03]**	**100.0%**	**––**
**Total (random effect, 96.7% CI)**							**0.89**	**[0.77; 1.03]**	**––**	**100.0%**
Heterogeneity: $Tau^2 = 0$; $Chi^2 = 3.7$, df = 4 (P = 0.45); $I^2 = 0\%$										
**Other antipsychotics**										
Breitbart et al. (chlorpromazine)	1996	High	2	11	5	13	0.47	[0.10; 2.24]	6.0%	3.1%
Garg et al. (quetiapin)	2022	High	6	15	6	15	1.00	[0.39; 2.59]	7.9%	8.2%
Girard et al. (ziprasidone)	2018	Low	73	192	65	190	1.11	[0.83; 1.49]	86.1%	88.6%
**Total (fixed effect, 96.7% CI)**			**81**	**218**	**76**	**218**	**1.06**	**[0.81; 1.40]**	**100.0%**	**––**
**Total (random effect, 96.7% CI)**							**1.07**	**[0.82; 1.41]**	**––**	**100.0%**
Heterogeneity: $Tau^2 = 0$; $Chi^2 = 1.36$, df = 2 (P = 0.51); $I^2 = 0\%$										
**Benzodiazepines**										
Breitbart et al. (lorazepam)	1996	High	2	11	2	6	0.55	[0.09; 3.42]	100.0%	100.0%
**Opioids**										
Atalan et al. (morphine)	2013	High	2	26	1	27	2.08	[0.16; 26.46]	100.0%	100.0%
**No control**										
Graham et al. (no control)	2006	High	0	4	2	6	0.29	[0.01; 6.00]	100.0%	100.0%

Heterogeneity: $Tau^2 = 0$; $Chi^2 = 8.19$, df = 10 (P = 0.61); $I^2 = 0\%$
Test for subgroup differences (common effect): $Chi^2 = 3.01$, df = 4 (P = 0.56)
Test for subgroup differences (random effects): $Chi^2 = 3.13$, df = 4 (P = 0.54)

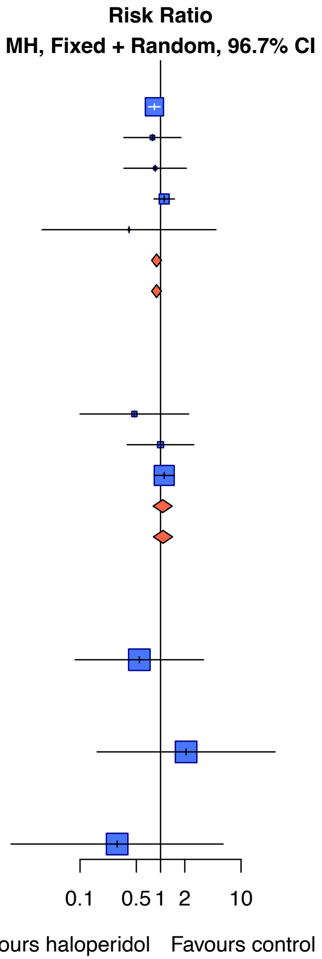

All–cause mortality divided by control intervention

Figure S2: Subgroup analysis of mortality for haloperidol versus placebo; low-risk of bias versus high-risk of bias trials

Study or Subgroup	Year	Haloperidol Events	Haloperidol Total	Placebo Events	Placebo Total	RR	96.7% CI	Weight (fixed)	Weight (random)
**Risk of Bias = Low**									
Andersen–Ranberg et al. (placebo)	2022	182	501	210	485	0.84	[0.71; 0.99]	73.4%	73.0%
EuRIDICE (placebo)	unpubl.	10	65	13	67	0.79	[0.35; 1.79]	4.4%	3.1%
Girard et al. (placebo)	2018	73	192	63	184	1.11	[0.83; 1.49]	22.1%	23.9%
**Total (fixed effect, 96.7% CI)**		**265**	**758**	**286**	**736**	**0.90**	**[0.78; 1.04]**	**100.0%**	**--**
**Total (random effect, 96.7% CI)**						**0.92**	**[0.73; 1.16]**	**--**	**100.0%**
Heterogeneity: $Tau^2 = 0.0136$; $Chi^2 = 3.23$, df = 2 (P = 0.20); $I^2 = 38\%$									
**Risk of Bias = High**									
Garg et al. (placebo)	2022	6	15	7	15	0.86	[0.35; 2.10]	76.0%	88.5%
ORIC–1 (placebo)	2017	1	16	2	13	0.41	[0.03; 4.88]	24.0%	11.5%
**Total (fixed effect, 96.7% CI)**		**7**	**31**	**9**	**28**	**0.75**	**[0.32; 1.75]**	**100.0%**	**--**
**Total (random effect, 96.7% CI)**						**0.79**	**[0.34; 1.83]**	**--**	**100.0%**
Heterogeneity: $Tau^2 = 0$; $Chi^2 = 0.38$, df = 1 (P = 0.54); $I^2 = 0\%$									
Heterogeneity: $Tau^2 = 0$; $Chi^2 = 3.70$, df = 4 (P = 0.45); $I^2 = 0\%$									
Test for subgroup differences (common effect): $Chi^2 = 0.20$, df = 1 (P = 0.66)									
Test for subgroup differences (random effects): $Chi^2 = 0.15$, df = 1 (P = 0.70)									

Risk Ratio
MH, Fixed + Random, 96.7% CI

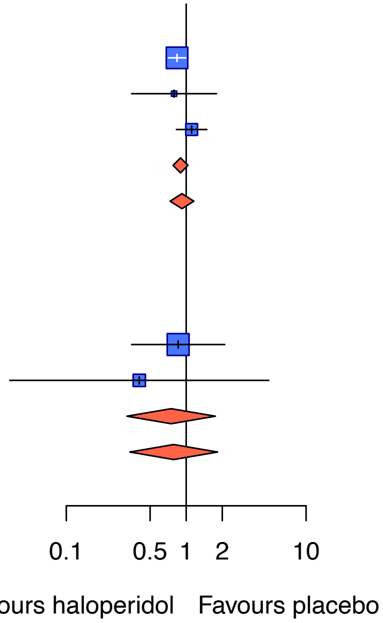

All–cause mortality: haloperidol vs. placebo, grouped by risk risk of bias

Figure S3: Sensitivity analysis best-worst case scenario of all-cause mortality

**Study or Subgroup**	**Haloperidol Events**	**Haloperidol Total**	**Control Events**	**Control Total**	**RR**	**96.7% CI**	**Weight (fixed)**	**Weight (random)**
**Placebo**								
Andersen–Ranberg et al.	182	510	215	490	0.81	[0.69; 0.96]	70.1%	56.1%
EuRIDICE	10	68	20	74	0.54	[0.26; 1.15]	6.1%	6.9%
Garg–placebo	6	15	7	15	0.86	[0.35; 2.10]	2.2%	4.9%
Girard–placebo	73	192	63	184	1.11	[0.83; 1.49]	20.6%	31.4%
ORIC–1	1	16	3	14	0.29	[0.03; 3.01]	1.0%	0.8%
**Total (fixed effect, 96.7% CI)**	**272**	**801**	**308**	**777**	**0.85**	**[0.74; 0.98]**	**100.0%**	**--**
**Total (random effect, 96.7% CI)**					**0.86**	**[0.66; 1.12]**	**--**	**100.0%**
Heterogeneity: $Tau^2 = 0.0259$; $Chi^2 = 6.66$, df = 4 (P = 0.15); $I^2 = 40\%$								
**Other antipsychotics**								
Breitbart – chlorpromazine	2	11	5	13	0.47	[0.10; 2.24]	6.0%	4.5%
Garg–quetiapin	6	15	6	15	1.00	[0.39; 2.59]	7.9%	11.5%
Girard–ziprasidone	73	192	65	190	1.11	[0.83; 1.49]	86.1%	84.1%
**Total (fixed effect, 96.7% CI)**	**81**	**218**	**76**	**218**	**1.06**	**[0.81; 1.40]**	**100.0%**	**--**
**Total (random effect, 96.7% CI)**					**1.07**	**[0.82; 1.41]**	**--**	**100.0%**
Heterogeneity: $Tau^2 = 0$; $Chi^2 = 1.36$, df = 2 (P = 0.51); $I^2 = 0\%$								
**Benzodiazepines**								
Breitbart – lorazepam	2	11	2	6	0.55	[0.09; 3.42]	100.0%	100.0%
**Opioids**								
Atalan	2	26	1	27	2.08	[0.16; 26.46]	100.0%	100.0%
**No control**								
Graham	0	4	2	6	0.29	[0.01; 6.00]	100.0%	100.0%

Heterogeneity: $Tau^2 = 0.0101$; $Chi^2 = 11.86$, df = 10 (P = 0.29); $I^2 = 16\%$
Test for subgroup differences (common effect): $Chi^2 = 3.82$, df = 4 (P = 0.43)
Test for subgroup differences (random effects): $Chi^2 = 3.10$, df = 4 (P = 0.54)

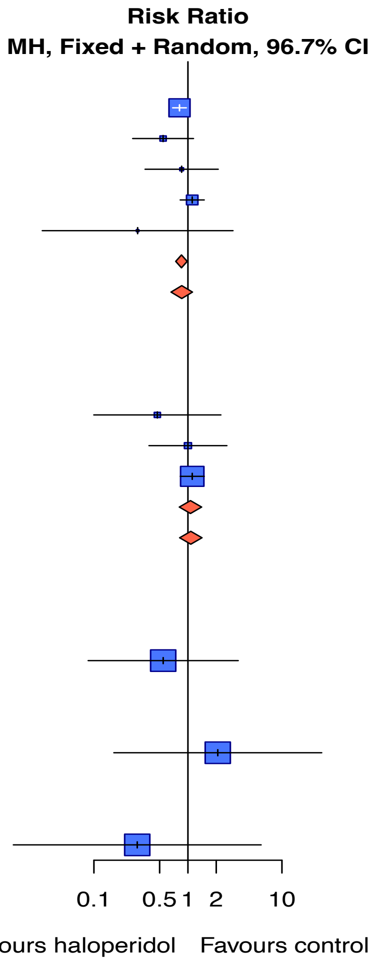

All–cause mortality divided by control intervention: Best–worst scenario.

Figure S4: Sensitivity analysis worst-best case scenario of all-cause mortality

Study or Subgroup	Haloperidol Events	Haloperidol Total	Control Events	Control Total	RR	96.7% CI	Weight (fixed)	Weight (random)
**Placebo**								
Andersen–Ranberg et al.	191	510	210	490	0.87	[0.74; 1.03]	71.4%	71.3%
EuRIDICE	13	68	13	74	1.09	[0.51; 2.32]	4.1%	3.4%
Garg–placebo	6	15	7	15	0.86	[0.35; 2.10]	2.3%	2.4%
Girard–placebo	73	192	63	184	1.11	[0.83; 1.49]	21.4%	22.6%
ORIC–1	1	16	2	14	0.44	[0.04; 5.29]	0.7%	0.3%
**Total (fixed effect, 96.7% CI)**	**284**	**801**	**295**	**777**	**0.93**	**[0.81; 1.07]**	**100.0%**	**--**
**Total (random effect, 96.7% CI)**					**0.93**	**[0.81; 1.07]**	**--**	**100.0%**
Heterogeneity: $Tau^2 = 0$; $Chi^2 = 2.96$, df = 4 (P = 0.56); $I^2 = 0\%$								
**Other antipsychotics**								
Breitbart – chlorpromazine	2	11	5	13	0.47	[0.10; 2.24]	6.0%	3.1%
Garg–quetiapin	6	15	6	15	1.00	[0.39; 2.59]	7.9%	8.2%
Girard–ziprasidone	73	192	65	190	1.11	[0.83; 1.49]	86.1%	88.6%
**Total (fixed effect, 96.7% CI)**	**81**	**218**	**76**	**218**	**1.06**	**[0.81; 1.40]**	**100.0%**	**--**
**Total (random effect, 96.7% CI)**					**1.07**	**[0.82; 1.41]**	**--**	**100.0%**
Heterogeneity: $Tau^2 = 0$; $Chi^2 = 1.36$, df = 2 (P = 0.51); $I^2 = 0\%$								
**Benzodiazepines**								
Breitbart – lorazepam	2	11	2	6	0.55	[0.09; 3.42]	100.0%	100.0%
**Opioids**								
Atalan	2	26	1	27	2.08	[0.16; 26.46]	100.0%	100.0%
**No control**								
Graham	0	4	2	6	0.29	[0.01; 6.00]	100.0%	100.0%

Heterogeneity: $Tau^2 = 0$; $Chi^2 = 6.89$, df = 10 (P = 0.74); $I^2 = 0\%$
Test for subgroup differences (common effect): $Chi^2 = 2.45$, df = 4 (P = 0.65)
Test for subgroup differences (random effects): $Chi^2 = 2.58$, df = 4 (P = 0.63)

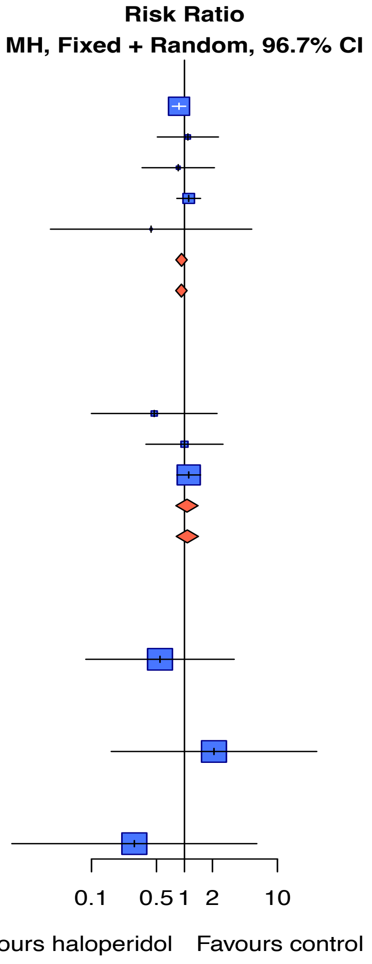

All–cause mortality divided by control intervention: worst–best scenario

Figure S5: TSA sensitivity analysis of all-cause mortality for haloperidol versus placebo (RR11%)

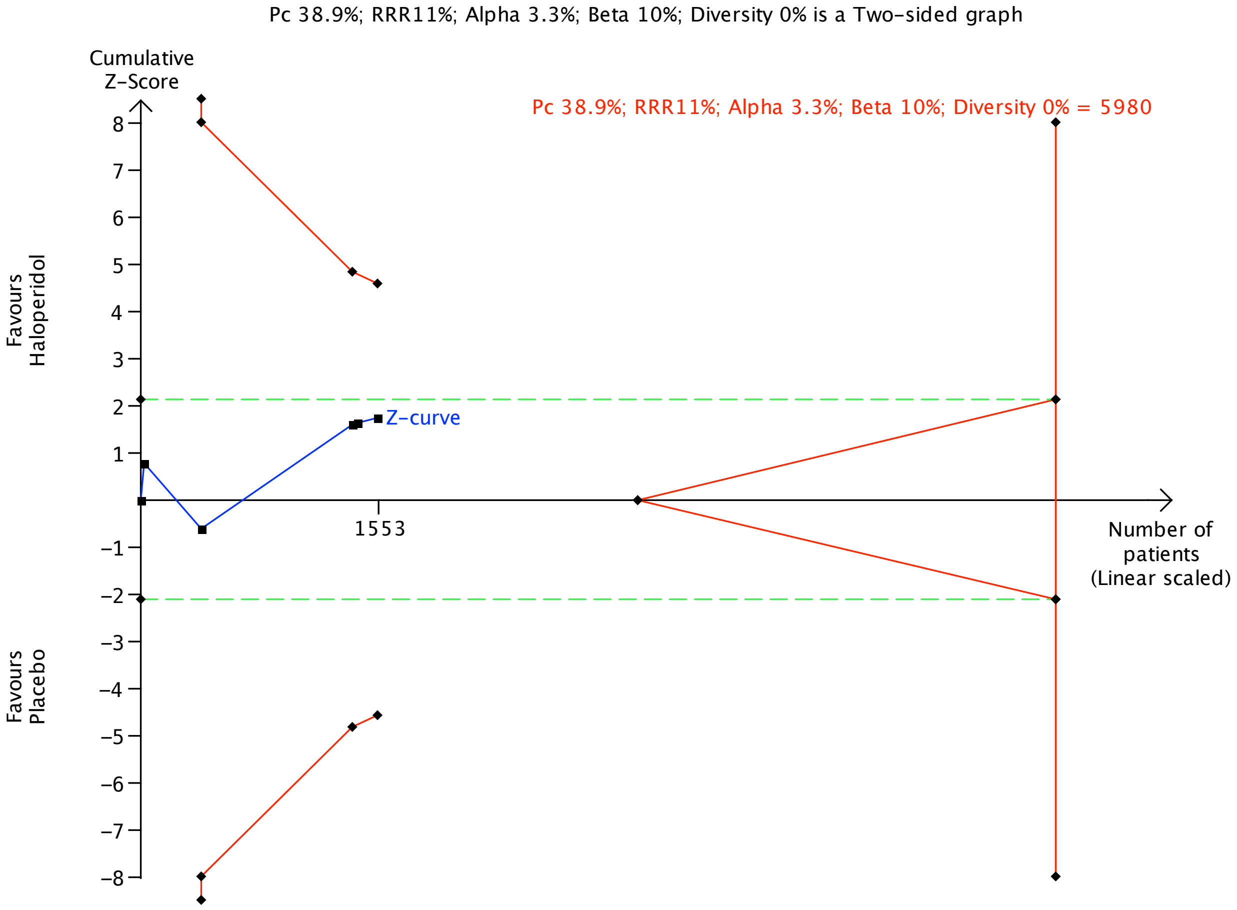

Trial Sequential Analysis of all-cause mortality for trials investigating haloperidol versus placebo. In this sensitivity analysis we used a control event rate of 38.9%, alfa-level of 3.3%, beta 10% (90% power) and as indicated by the meta-analysis (Figure 1 manuscript) a relative risk reduction of 11% and diversity of 0%. TSA showed that 26% of the required information size was accrued and we are therefore unable to reject or accept a RR of 11%.

Figure S6: TSA of all-cause mortality for haloperidol versus other antipsychotics

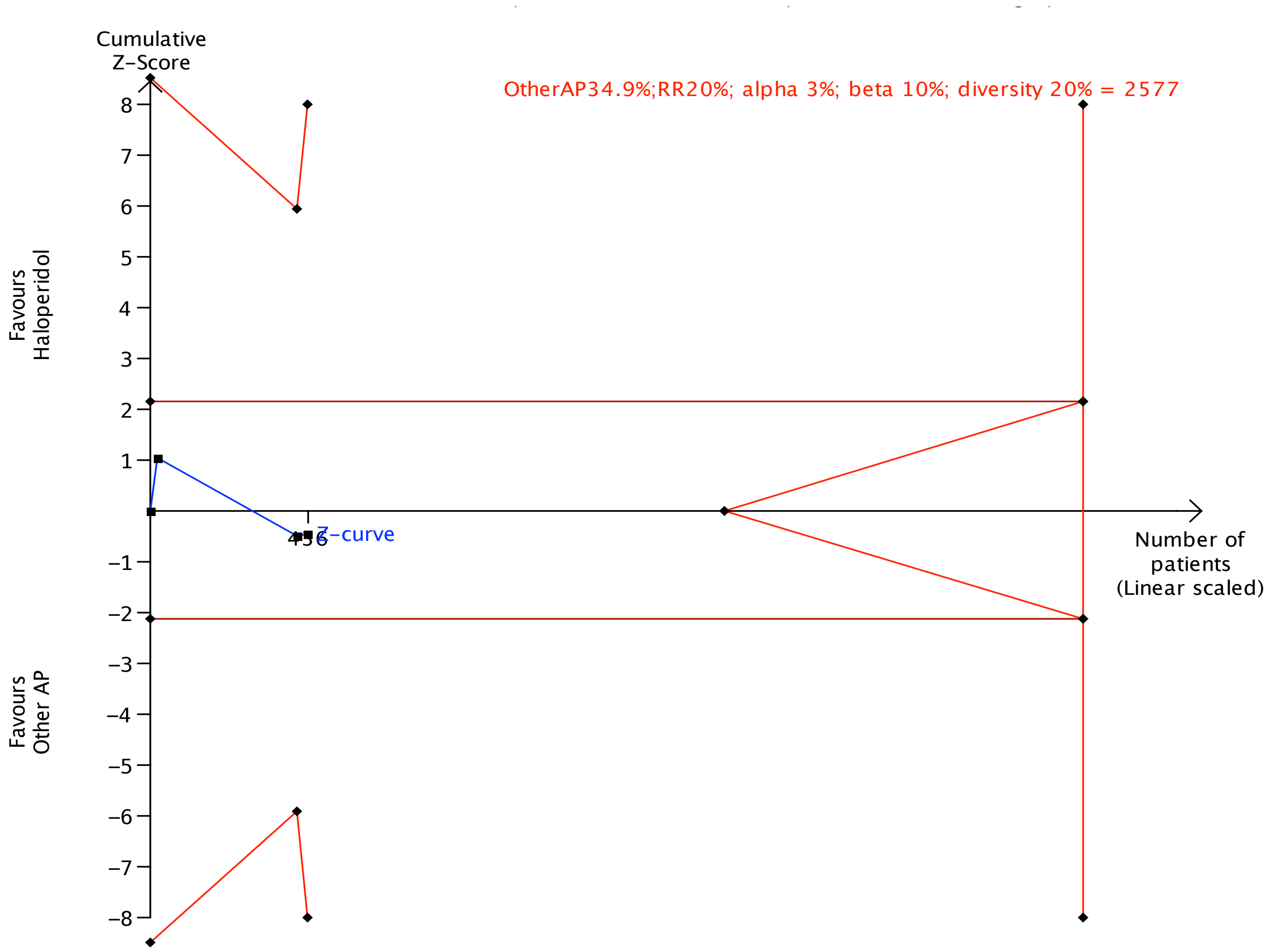

TSA of all-cause mortality for trials investigating haloperidol versus other Antipsychotics (Chlorpromazine, quetiapine and ziprasidone). We used a control event rate of 35% as indicated by the meta-analysis, a RRR/RRI of 20%, alfa-level of 3.3%, beta 10% (90% power) and diversity of 20%. TSA showed that 17% of the required information size was accrued and we are therefore unable to reject or accept a RRR/RRI of 20%.

Figure S7: SAE/SAR highest proportion; haloperidol versus all comparators

Study or Subgroup	Year	Risk of Bias	Haloperidol Events	Haloperidol Total	Control Events	Control Total	RR	96.7% CI	Weight (fixed)	Weight (random)
**Placebo**										
Andersen–Ranberg et al. (placebo)	2022	Low	182	501	210	485	0.84	[0.71; 0.99]	63.6%	52.2%
EuRIDICE (placebo)	unpubl.	High	48	65	48	67	1.03	[0.82; 1.29]	14.1%	28.5%
Garg et al. (placebo)	2022	High	6	15	7	15	0.86	[0.35; 2.10]	2.1%	1.8%
Girard et al. (placebo)	2018	Low	73	192	63	184	1.11	[0.83; 1.49]	19.2%	17.1%
ORIC–1 (placebo)	2017	High	2	16	3	13	0.54	[0.09; 3.20]	1.0%	0.5%
**Total (fixed effect, 96.7% CI)**			**311**	**789**	**331**	**764**	**0.92**	**[0.81; 1.04]**	**100.0%**	**––**
**Total (random effect, 96.7% CI)**							**0.94**	**[0.81; 1.10]**	**––**	**100.0%**
Heterogeneity: $Tau^2 = 0.0045$; $Chi^2 = 4.86$, df = 4 (P = 0.30); $I^2 = 18\%$										
**Other antipsychotics**										
Breitbart et al. (chlorpromazine)	1996	High	2	11	5	13	0.47	[0.10; 2.24]	6.0%	3.1%
Garg et al. (quetiapin)	2022	High	6	15	6	15	1.00	[0.39; 2.59]	7.9%	8.2%
Girard et al. (ziprasidone)	2018	Low	73	192	65	190	1.11	[0.83; 1.49]	86.1%	88.6%
Han et al. (risperidone)	2004	High	0	12	0	12			0.0%	0.0%
**Total (fixed effect, 96.7% CI)**			**81**	**230**	**76**	**230**	**1.06**	**[0.81; 1.40]**	**100.0%**	**––**
**Total (random effect, 96.7% CI)**							**1.07**	**[0.82; 1.41]**	**––**	**100.0%**
Heterogeneity: $Tau^2 = 0$; $Chi^2 = 1.36$, df = 2 (P = 0.51); $I^2 = 0\%$										
**Benzodiazepines**										
Breitbart et al. (lorazepam)	1996	High	2	11	2	6	0.55	[0.09; 3.42]	100.0%	100.0%
**Dexmedetomidine**										
Bakri et al. (dexmedetomidine)	2015	High	0	16	0	32			––	––
**Opioids**										
Atalan et al. (morphine)	2013	High	8	26	7	27	1.19	[0.47; 3.02]	100.0%	100.0%
**Antiemetics**										
Bakri et al. (ondansetrone)	2015	High	0	16	0	32			––	––
**No control**										
Graham et al. (no control)	2006	High	0	4	2	6	0.29	[0.01; 6.00]	100.0%	100.0%
Heterogeneity: $Tau^2 = 0$; $Chi^2 = 8.56$, df = 10 (P = 0.57); $I^2 = 0\%$										
Test for subgroup differences (common effect): $Chi^2 = 2.53$, df = 4 (P = 0.64)										
Test for subgroup differences (random effects): $Chi^2 = 2.14$, df = 4 (P = 0.71)										

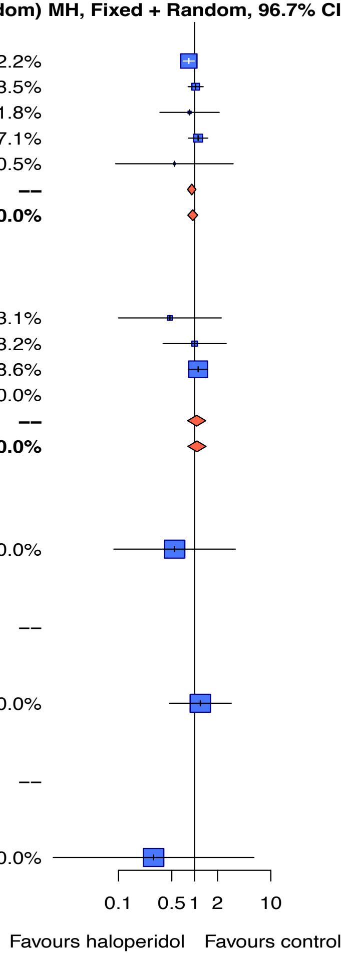

Serious Adverse Events (highest proportion) subgrouped by control intervention

Figure S8: Subgroup analysis of SAE/SAR highest proportion for haloperidol versus placebo; low-risk of bias versus high-risk of bias trials

Study or Subgroup	Year	Haloperidol Events	Haloperidol Total	Placebo Events	Placebo Total	RR	96.7% CI	Weight (fixed)	Weight (random)
**Risk of Bias = Low**									
Andersen–Ranberg et al. (placebo)	2022	182	501	210	485	0.84	[0.71; 0.99]	63.6%	45.3%
Girard et al. (placebo)	2018	73	192	63	184	1.11	[0.83; 1.49]	19.2%	20.7%
**Total (fixed effect, 96.7% CI)**		**255**	**693**	**273**	**669**	**0.90**	**[0.78; 1.04]**	**82.8%**	**––**
**Total (random effect, 96.7% CI)**						**0.94**	**[0.70; 1.27]**	**––**	**66.0%**
Heterogeneity: $Tau^2 = 0.0268$; $Chi^2 = 3.13$, df = 1 (P = 0.08); $I^2 = 68\%$									
**Risk of Bias = High**									
EuRIDICE (placebo)	unpubl.	48	65	48	67	1.03	[0.82; 1.29]	14.1%	30.6%
Garg et al. (placebo)	2022	6	15	7	15	0.86	[0.35; 2.10]	2.1%	2.7%
ORIC–1 (placebo)	2017	2	16	3	13	0.54	[0.09; 3.20]	1.0%	0.7%
**Total (fixed effect, 96.7% CI)**		**56**	**96**	**58**	**95**	**0.98**	**[0.78; 1.23]**	**17.2%**	**––**
**Total (random effect, 96.7% CI)**						**1.01**	**[0.81; 1.26]**	**––**	**34.0%**
Heterogeneity: $Tau^2 = 0$; $Chi^2 = 0.82$, df = 2 (P = 0.66); $I^2 = 0\%$									
**Total (fixed effect, 96.7% CI)**		**311**	**789**	**331**	**764**	**0.92**	**[0.81; 1.04]**	**100.0%**	**––**
**Total (random effect, 96.7% CI)**						**0.94**	**[0.81; 1.10]**	**––**	**100.0%**

Heterogeneity: $Tau^2 = 0.0045$; $Chi^2 = 4.86$, df = 4 (P = 0.30); $I^2 = 18\%$
Test for overall effect (common effect): Z = −1.49 (P = 0.14)
Test for overall effect (random effects): Z = −0.82 (P = 0.41)
Test for subgroup differences (common effect): $Chi^2 = 0.44$, df = 1 (P = 0.51)
Test for subgroup differences (random effects): $Chi^2 = 0.15$, df = 1 (P = 0.69)

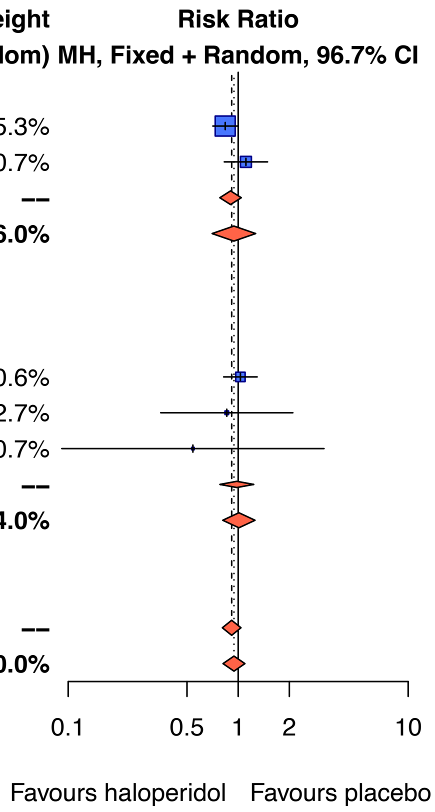

Highest proportion SAE: High risk of bias versus low risk of bias

Figure S9: Sensitivity analysis best-worst case scenario of SAE/SAR highest proportion

Study or Subgroup	Year	Risk of Bias	Haloperidol Events	Haloperidol Total	Control Events	Control Total	RR	96.7% CI	Weight (fixed)	Weight (random)
**Placebo**										
Andersen–Ranberg et al.	2022	Low	182	510	191	490	0.92	[0.77; 1.09]	59.7%	38.0%
EuRIDICE	unpubl.	High	48	68	51	74	1.02	[0.81; 1.30]	15.0%	30.6%
Garg–placebo	2022	High	6	15	6	15	1.00	[0.39; 2.59]	1.8%	4.0%
Girard–placebo	2018	Low	73	192	73	184	0.96	[0.73; 1.26]	22.8%	26.4%
ORIC–1	2017	High	2	16	2	14	0.88	[0.12; 6.36]	0.7%	1.0%
**Total (fixed effect, 96.7% CI)**			**311**	**801**	**323**	**777**	**0.94**	**[0.83; 1.07]**	**100.0%**	**––**
**Total (random effect, 96.7% CI)**							**0.95**	**[0.84; 1.08]**	**––**	**100.0%**
Heterogeneity: $Tau^2 = 0$; $Chi^2 = 0.73$, df = 4 (P = 0.95); $I^2 = 0\%$										
**Other antipsychotics**										
Breitbart – chlorpromazine	1996	High	2	11	2	13	1.18	[0.17; 8.26]	2.2%	3.2%
Garg–quetiapin	2022	High	6	15	6	15	1.00	[0.39; 2.59]	7.2%	12.6%
Girard–ziprasidone	2018	Low	73	192	73	190	0.99	[0.75; 1.31]	87.7%	83.0%
Han	2004	High	0	14	2	14	0.20	[0.01; 4.94]	3.0%	1.2%
**Total (fixed effect, 96.7% CI)**			**81**	**232**	**83**	**232**	**0.97**	**[0.75; 1.26]**	**100.0%**	**––**
**Total (random effect, 96.7% CI)**							**0.98**	**[0.76; 1.28]**	**––**	**100.0%**
Heterogeneity: $Tau^2 = 0$; $Chi^2 = 1.18$, df = 3 (P = 0.76); $I^2 = 0\%$										
**Benzodiazepines**										
Breitbart – lorazepam	1996	High	2	11	2	6	0.55	[0.09; 3.42]	100.0%	100.0%
**Dexmedetomidine**										
Bakri–dexmed	2015	High	0	32	16	32	0.03	[0.00; 0.62]	100.0%	100.0%
**Opioids**										
Atalan	2013	High	8	26	8	27	1.04	[0.43; 2.53]	100.0%	100.0%
**Antiemetics**										
Bakri–ondan	2015	High	0	32	16	32	0.03	[0.00; 0.62]	100.0%	100.0%
**No control**										
Graham	2006	High	0	4	0	6			––	––

Heterogeneity: $Tau^2 = 0.0160$; $Chi^2 = 16.36$, df = 12 (P = 0.18); $I^2 = 27\%$
Test for subgroup differences (common effect): $Chi^2 = 12.32$, df = 5 (P = 0.03)
Test for subgroup differences (random effects): $Chi^2 = 12.42$, df = 5 (P = 0.03)

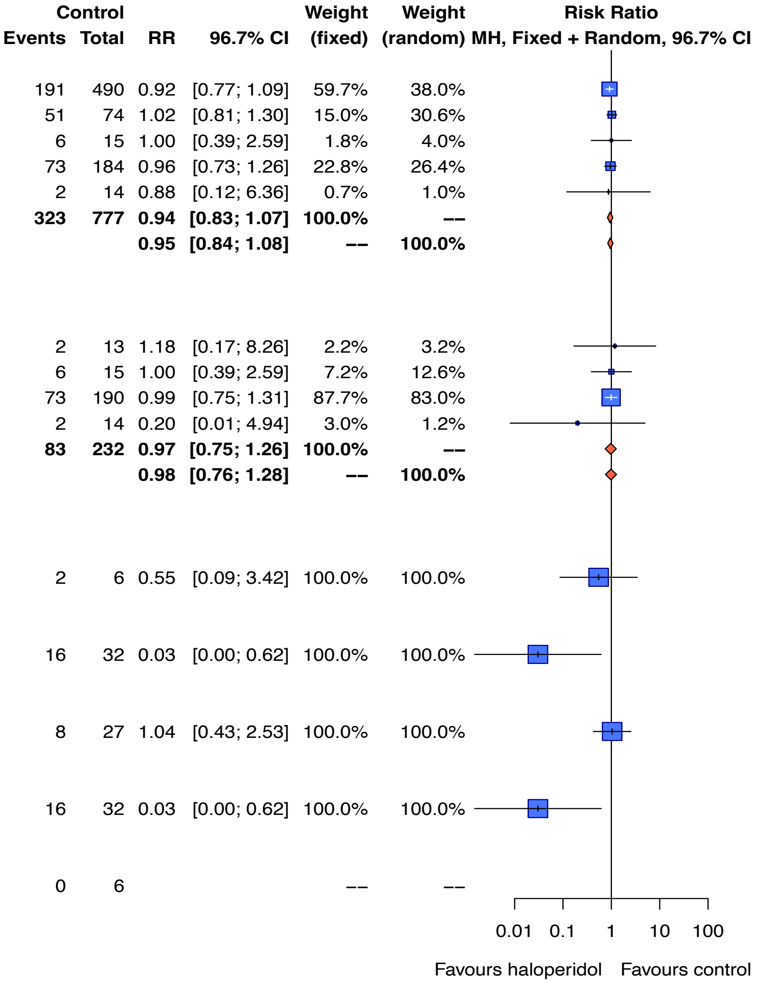

Serious Adverse Events (highest proportion) subgrouped by control intervention: best–worst scenario

Figure S10: Sensitivity analysis worst-best case scenario of SAE/SAR highest proportion

Study or Subgroup	Year	Risk of Bias	Haloperidol Events	Haloperidol Total	Control Events	Control Total	RR	96.7% CI	Weight (fixed)	Weight (random)
**Placebo**										
Andersen–Ranberg et al.	2022	Low	191	510	210	490	0.87	[0.74; 1.03]	64.0%	34.4%
EuRIDICE	unpubl.	High	51	68	48	74	1.16	[0.91; 1.46]	13.7%	30.1%
Garg–placebo	2022	High	6	15	7	15	0.86	[0.35; 2.10]	2.1%	7.0%
Girard–placebo	2018	Low	73	192	63	184	1.11	[0.83; 1.49]	19.2%	26.5%
ORIC–1	2017	High	2	16	3	14	0.58	[0.10; 3.47]	1.0%	2.0%
**Total (fixed effect, 96.7% CI)**			**323**	**801**	**331**	**777**	**0.95**	**[0.84; 1.08]**	**100.0%**	**--**
**Total (random effect, 96.7% CI)**							**1.00**	**[0.84; 1.19]**	**--**	**100.0%**
Heterogeneity: $Tau^2 = 0.0103$; $Chi^2 = 5.92$, df = 4 (P = 0.21); $I^2 = 32\%$										
**Other antipsychotics**										
Breitbart – chlorpromazine	1996	High	2	11	5	13	0.47	[0.10; 2.24]	6.0%	7.3%
Garg–quetiapin	2022	High	6	15	6	15	1.00	[0.39; 2.59]	7.9%	17.4%
Girard–ziprasidone	2018	Low	73	192	65	190	1.11	[0.83; 1.49]	85.5%	73.5%
Han	2004	High	2	14	0	14	5.00	[0.20; 123.49]	0.7%	1.8%
**Total (fixed effect, 96.7% CI)**			**83**	**232**	**76**	**232**	**1.09**	**[0.83; 1.43]**	**100.0%**	**--**
**Total (random effect, 96.7% CI)**							**1.08**	**[0.83; 1.43]**	**--**	**100.0%**
Heterogeneity: $Tau^2 = 0$; $Chi^2 = 2.39$, df = 3 (P = 0.49); $I^2 = 0\%$										
**Benzodiazepines**										
Breitbart – lorazepam	1996	High	2	11	2	6	0.55	[0.09; 3.42]	100.0%	100.0%
**Dexmedetomidine**										
Bakri–dexmed	2015	High	16	32	0	32	33.00	[1.62; 672.52]	100.0%	100.0%
**Opioids**										
Atalan	2013	High	8	26	7	27	1.19	[0.47; 3.02]	100.0%	100.0%
**Antiemetics**										
Bakri–ondan	2015	High	16	32	0	32	33.00	[1.62; 672.52]	100.0%	100.0%
**No control**										
Graham	2006	High	0	4	2	6	0.29	[0.01; 6.00]	100.0%	100.0%

Heterogeneity: $Tau^2 = 0.0377$; $Chi^2 = 23.20$, df = 13 (P = 0.04); $I^2 = 44\%$
Test for subgroup differences (common effect): $Chi^2 = 14.66$, df = 6 (P = 0.02)
Test for subgroup differences (random effects): $Chi^2 = 13.74$, df = 6 (P = 0.03)

Risk Ratio MH, Fixed + Random, 96.7% CI

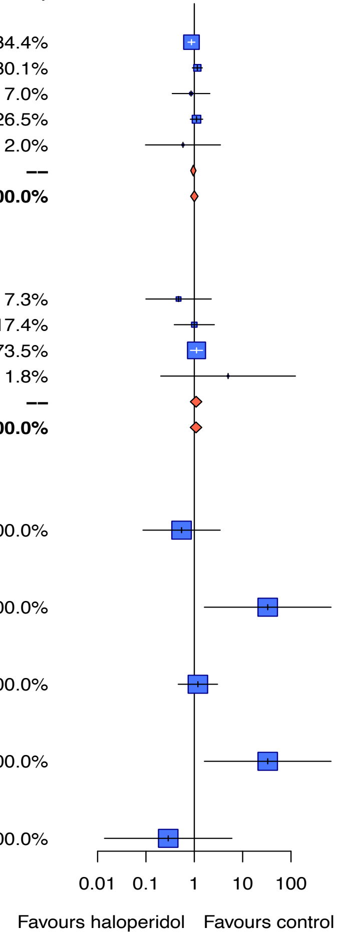

Serious Adverse Events (highest proportion) subgrouped by control intervention: best–worst scenario

Figure S11: TSA of SAE/SAR highest proportion for haloperidol versus placebo (RR20%)

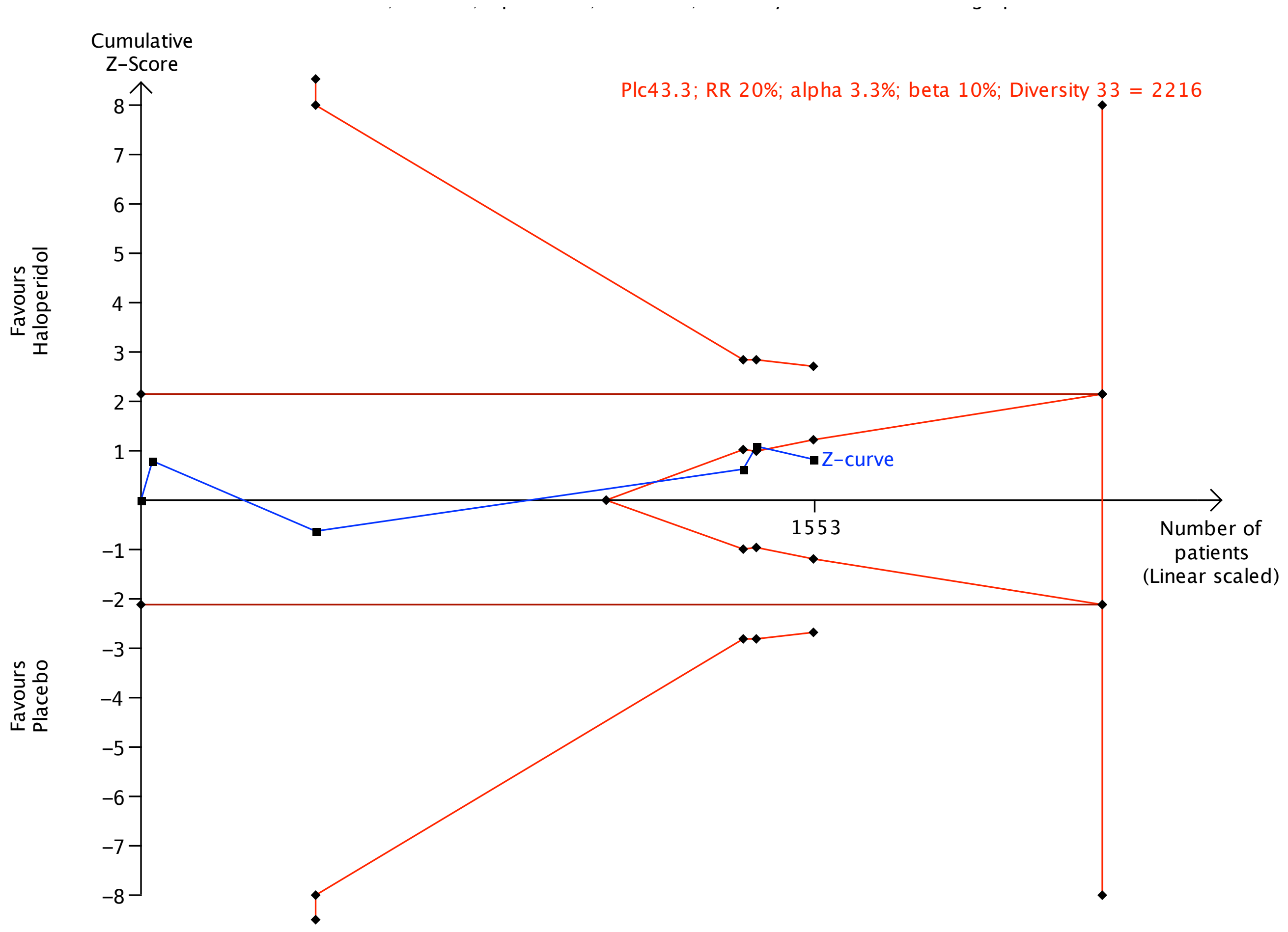

TSA of SAE/SAR highest proportion for trials investigating haloperidol versus placebo. We used a control event rate of 43%, a RR of 20%, alfa-level of 3.3%, beta 10% (90% power) and diversity of 33% as indicated by the meta-analysis. TSA showed that the z-curve reached the futility area meaning that haloperidol does not cause a 20% relative change in experiencing SAE/SAR (highest proportion), randomising more patients will not change this result.

Figure S12: TSA sensitivity analysis of SAE/SAR highest proportion for haloperidol versus placebo (RR 6%)

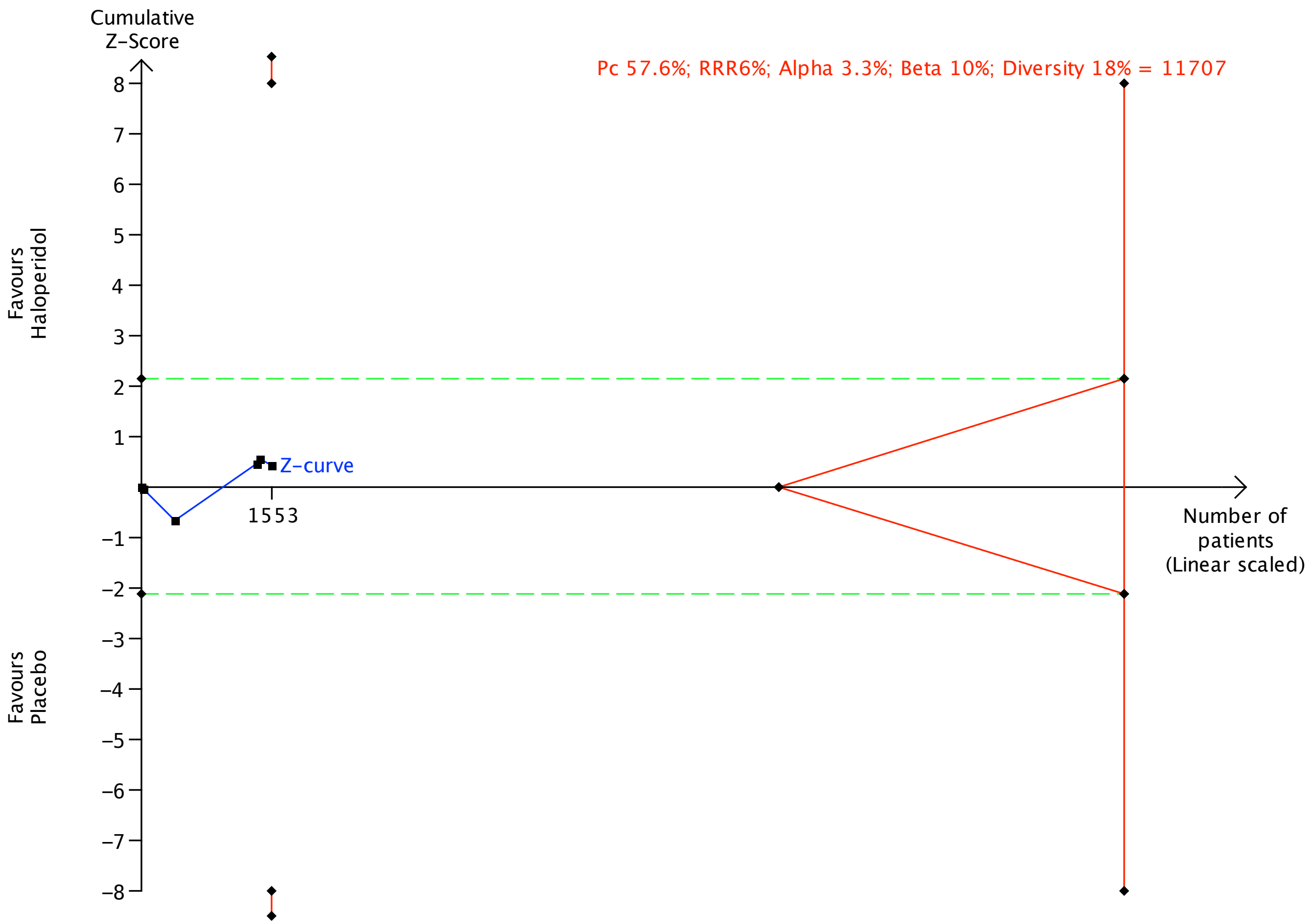

Trial Sequential Analysis of SAE highest proportion for trials investigating haloperidol versus placebo. In this sensitivity analysis we used a control event rate of 43.3%, alfa-level of 3.3%, beta 10% (90% power) and as indicated by the meta-analysis (Figure S7) a relative risk reduction of 6% and diversity of 0%. TSA showed that 6% of the required information size was accrued and we are therefore unable to reject or accept a RR of 6%.

Figure S13: TSA of SAE/SAR for haloperidol versus other antipsychotics

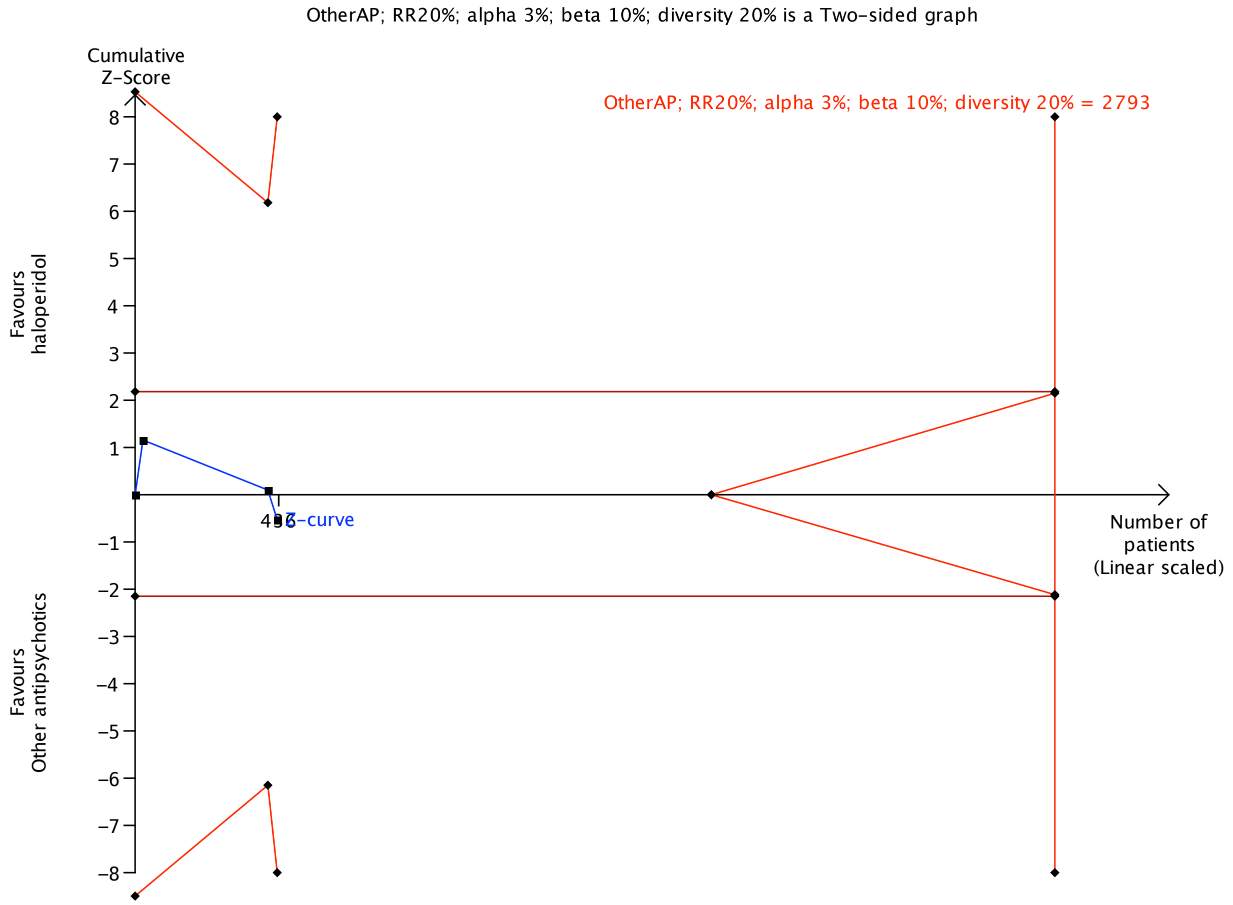

TSA of SAE/SAR highest proportion for trials investigating haloperidol versus other Antipsychotics (Chlorpromazine, quetiapine, ziprasidone, risperidone). We used a control event rate of 33% as indicated by the meta-analysis, a RRR/RRI of 20%, alfa-level of 3.3%, beta 10% (90% power) and diversity of 20%. TSA showed that 16% of the required information size was accrued and we are therefore unable to reject or confirm a RRR/RRI of 20%.

Figure S14: cumulated SAEs/SARs; haloperidol versus all comparators

Study or Subgroup	Year	Risk of Bias	Haloperidol Events	Haloperidol Total	Control Events	Control Total	RR	95% CI	Weight (fixed)	Weight (random)
**Placebo**										
Andersen–Ranberg et al. (placebo)	2022	Low	193	501	219	485	0.85	[0.74; 0.99]	49.7%	17.2%
EuRIDICE (placebo)	unpubl.	High	65	65	67	67	1.00	[0.97; 1.03]	14.9%	40.9%
Garg et al. (placebo)	2022	High	6	15	7	15	0.86	[0.38; 1.95]	1.6%	0.9%
Girard et al. (placebo)	2018	Low	146	192	134	184	1.04	[0.93; 1.18]	30.6%	21.9%
ORIC–1 (placebo)	2017	High	16	16	13	13	1.00	[0.87; 1.14]	3.3%	19.1%
**Total (fixed effect, 96.7% CI)**			**426**	**789**	**440**	**764**	**0.94**	**[0.87; 1.02]**	**100.0%**	**--**
**Total (random effect, 96.7% CI)**							**0.97**	**[0.85; 1.11]**	**--**	**100.0%**
Heterogeneity: $Tau^2 = 0.0159$; $Chi^2 = 23.31$, df = 4 ($P < 0.01$); $I^2 = 83\%$										
**Other antipsychotics**										
Breitbart et al. (chlorpromazine)	1996	High	2	11	5	13	0.47	[0.11; 1.97]	3.1%	1.3%
Garg et al. (quetiapin)	2022	High	6	15	6	15	1.00	[0.42; 2.40]	4.1%	3.5%
Girard et al. (ziprasidone)	2018	Low	146	192	135	190	1.07	[0.95; 1.21]	92.8%	95.2%
Han et al. (risperidone)	2004	High	0	12	0	12			0.0%	0.0%
**Total (fixed effect, 96.7% CI)**			**154**	**230**	**146**	**230**	**1.05**	**[0.93; 1.18]**	**100.0%**	**--**
**Total (random effect, 96.7% CI)**							**1.06**	**[0.94; 1.20]**	**--**	**100.0%**
Heterogeneity: $Tau^2 = 0$; $Chi^2 = 1.31$, df = 2 ($P = 0.52$); $I^2 = 0\%$										
**Benzodiazepines**										
Breitbart et al. (lorazepam)	1996	High	2	11	2	6	0.55	[0.10; 2.95]	100.0%	100.0%
**Dexmedetomidine**										
Bakri et al. (dexmedetomidine)	2015	High	0	32	0	32			--	--
**Opioids**										
Atalan et al. (morphine)	2013	High	16	26	9	27	1.85	[1.00; 3.41]	100.0%	100.0%
**Antiemetics**										
Bakri et al. (ondansetrone)	2015	High	0	32	0	32			--	--
**No control**										
Graham et al. (no control)	2006	High	0	4	2	6	0.29	[0.02; 4.70]	100.0%	100.0%

Heterogeneity: $Tau^2 = 0.0037$; $Chi^2 = 16.30$, df = 10 ($P = 0.09$); $I^2 = 39\%$
Test for subgroup differences (common effect): $Chi^2 = 7.58$, df = 4 ($P = 0.11$)
Test for subgroup differences (random effects): $Chi^2 = 5.78$, df = 4 ($P = 0.22$)

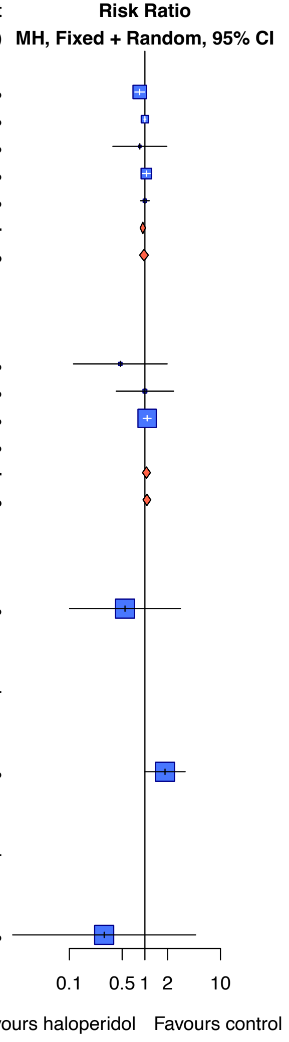

Serious Adverse Events (cummulated) subgrouped by control intervention

Figure S15: Subgroup analysis of cumulated SAEs/SARs for haloperidol versus placebo; low-risk of bias versus high-risk of bias trials

Study or Subgroup	Year	Haloperidol Events	Haloperidol Total	Placebo Events	Placebo Total	RR	96.7% CI	Weight (fixed)	Weight (random)
**Risk of Bias = Low**									
Andersen–Ranberg et al. (placebo)	2022	182	501	210	485	0.84	[0.71; 0.99]	63.6%	45.3%
Girard et al. (placebo)	2018	73	192	63	184	1.11	[0.83; 1.49]	19.2%	20.7%
**Total (fixed effect, 96.7% CI)**		**255**	**693**	**273**	**669**	**0.90**	**[0.78; 1.04]**	**82.8%**	**--**
**Total (random effect, 96.7% CI)**						**0.94**	**[0.70; 1.27]**	**--**	**66.0%**
Heterogeneity: $Tau^2 = 0.0268$; $Chi^2 = 3.13$, df = 1 (P = 0.08); $I^2 = 68\%$									
**Risk of Bias = High**									
EuRIDICE (placebo)	unpubl.	48	65	48	67	1.03	[0.82; 1.29]	14.1%	30.6%
Garg et al. (placebo)	2022	6	15	7	15	0.86	[0.35; 2.10]	2.1%	2.7%
ORIC–1 (placebo)	2017	2	16	3	13	0.54	[0.09; 3.20]	1.0%	0.7%
**Total (fixed effect, 96.7% CI)**		**56**	**96**	**58**	**95**	**0.98**	**[0.78; 1.23]**	**17.2%**	**--**
**Total (random effect, 96.7% CI)**						**1.01**	**[0.81; 1.26]**	**--**	**34.0%**
Heterogeneity: $Tau^2 = 0$; $Chi^2 = 0.82$, df = 2 (P = 0.66); $I^2 = 0\%$									
**Total (fixed effect, 96.7% CI)**		**311**	**789**	**331**	**764**	**0.92**	**[0.81; 1.04]**	**100.0%**	**--**
**Total (random effect, 96.7% CI)**						**0.94**	**[0.81; 1.10]**	**--**	**100.0%**

Heterogeneity: $Tau^2 = 0.0045$; $Chi^2 = 4.86$, df = 4 (P = 0.30); $I^2 = 18\%$
Test for overall effect (common effect): Z = –1.49 (P = 0.14)
Test for overall effect (random effects): Z = –0.82 (P = 0.41)
Test for subgroup differences (common effect): $Chi^2 = 0.44$, df = 1 (P = 0.51)
Test for subgroup differences (random effects): $Chi^2 = 0.15$, df = 1 (P = 0.69)

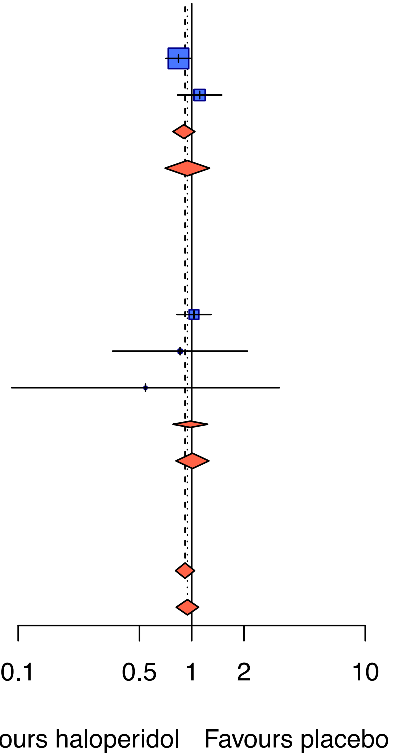

Highest proportion SAE: High risk of bias versus low risk of bias

Figure S16: Sensitivity analysis best-worst case scenario of cumulated SAEs/SARs

Study or Subgroup	Year	Risk of Bias	Haloperidol Events	Haloperidol Total	Control Events	Control Total	RR	96.7% CI	Weight (fixed)	Weight (random)
**Placebo**										
Andersen–Ranberg et al.	2022	Low	193	510	224	490	0.83	[0.71; 0.97]	49.8%	18.3%
EuRIDICE	unpubl.	High	65	68	74	74	0.96	[0.90; 1.01]	15.6%	36.7%
Garg–placebo	2022	High	6	15	7	15	0.86	[0.35; 2.10]	1.5%	1.0%
Girard–placebo	2018	Low	146	192	134	184	1.04	[0.92; 1.19]	29.8%	22.9%
ORIC–1	2017	High	16	16	14	14	1.00	[0.87; 1.15]	3.4%	21.1%
**Total (fixed effect, 96.7% CI)**			**426**	**801**	**453**	**777**	**0.92**	**[0.84; 1.00]**	**100.0%**	**--**
**Total (random effect, 96.7% CI)**							**0.96**	**[0.86; 1.06]**	**--**	**100.0%**
Heterogeneity: $Tau^2 = 0.0060$; $Chi^2 = 10.49$, df = 4 (P = 0.03); $I^2 = 62\%$										
**Other antipsychotics**										
Breitbart – chlorpromazine	1996	High	2	11	5	13	0.47	[0.10; 2.24]	3.1%	1.4%
Garg–quetiapin	2022	High	6	15	6	15	1.00	[0.39; 2.59]	4.0%	3.7%
Girard–ziprasidone	2018	Low	146	192	135	190	1.07	[0.94; 1.22]	91.2%	94.5%
Han	2004	High	0	14	2	14	0.20	[0.01; 4.94]	1.7%	0.3%
**Total (fixed effect, 96.7% CI)**			**154**	**232**	**148**	**232**	**1.03**	**[0.90; 1.18]**	**100.0%**	**--**
**Total (random effect, 96.7% CI)**							**1.06**	**[0.93; 1.21]**	**--**	**100.0%**
Heterogeneity: $Tau^2 = 0$; $Chi^2 = 2.66$, df = 3 (P = 0.45); $I^2 = 0\%$										
**Benzodiazepines**										
Breitbart – lorazepam	1996	High	2	11	2	6	0.55	[0.09; 3.42]	100.0%	100.0%
**Dexmedetomidine**										
Bakri–dexmed	2015	High	0	32	0	32			--	--
**Opioids**										
Atalan	2013	High	16	26	9	27	1.85	[0.95; 3.60]	100.0%	100.0%
**Antiemetics**										
Bakri–ondan	2015	High	0	32	0	32			--	--
**No control**										
Graham	2006	High	0	4	2	6	0.29	[0.01; 6.00]	100.0%	100.0%

Heterogeneity: $Tau^2 = 0.0043$; $Chi^2 = 17.28$, df = 11 (P = 0.10); $I^2 = 36\%$
Test for subgroup differences (common effect): $Chi^2 = 8.09$, df = 4 (P = 0.09)
Test for subgroup differences (random effects): $Chi^2 = 6.85$, df = 4 (P = 0.14)

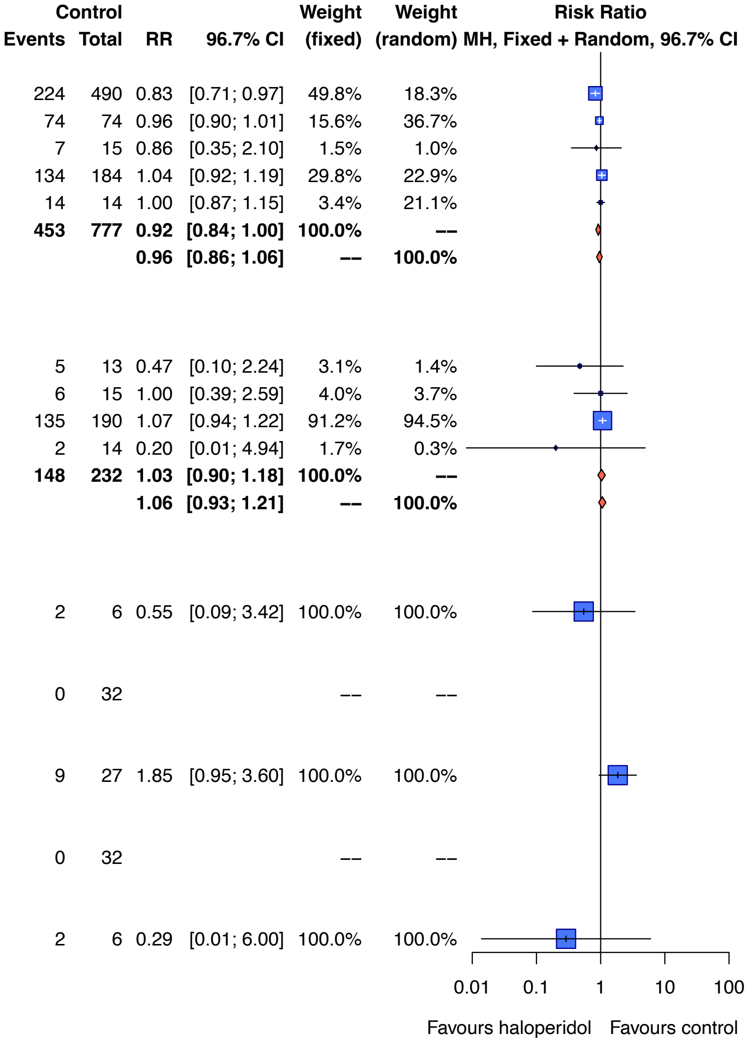

Serious Adverse Events (cummulated) subgrouped by control intervention: best–worst scenario

Figure S17: Sensitivity analysis worst-best case scenario of cumulated SAEs/SARs

Study or Subgroup	Year	Risk of Bias	Haloperidol Events	Haloperidol Total	Control Events	Control Total	RR	96.7% CI	Weight (fixed)	Weight (random)
**Placebo**										
Andersen–Ranberg et al.	2022	Low	202	510	219	490	0.89	[0.76; 1.04]	50.1%	20.9%
EuRIDICE	unpubl.	High	68	68	67	74	1.10	[1.02; 1.20]	14.5%	31.4%
Garg–placebo	2022	High	6	15	7	15	0.86	[0.35; 2.10]	1.6%	1.4%
Girard–placebo	2018	Low	146	192	134	184	1.04	[0.92; 1.19]	30.7%	24.6%
ORIC–1	2017	High	16	16	13	14	1.07	[0.92; 1.25]	3.2%	21.7%
**Total (fixed effect, 96.7% CI)**			**438**	**801**	**440**	**777**	**0.97**	**[0.89; 1.06]**	**100.0%**	**--**
**Total (random effect, 96.7% CI)**							**1.02**	**[0.89; 1.18]**	**--**	**100.0%**
Heterogeneity: $Tau^2 = 0.0142$; $Chi^2 = 16.63$, df = 4 ($P < 0.01$); $I^2 = 76\%$										
**Other antipsychotics**										
Breitbart – chlorpromazine	1996	High	2	11	5	13	0.47	[0.10; 2.24]	3.1%	1.8%
Garg–quetiapin	2022	High	6	15	6	15	1.00	[0.39; 2.59]	4.1%	4.8%
Girard–ziprasidone	2018	Low	146	192	135	190	1.07	[0.94; 1.22]	92.4%	92.9%
Han	2004	High	2	14	0	14	5.00	[0.20; 123.49]	0.3%	0.4%
**Total (fixed effect, 96.7% CI)**			**156**	**232**	**146**	**232**	**1.06**	**[0.93; 1.21]**	**100.0%**	**--**
**Total (random effect, 96.7% CI)**							**1.07**	**[0.94; 1.21]**	**--**	**100.0%**
Heterogeneity: $Tau^2 = 0$; $Chi^2 = 2.33$, df = 3 ($P = 0.51$); $I^2 = 0\%$										
**Benzodiazepines**										
Breitbart – lorazepam	1996	High	2	11	2	6	0.55	[0.09; 3.42]	100.0%	100.0%
**Dexmedetomidine**										
Bakri–dexmed	2015	High	0	32	0	32			--	--
**Opioids**										
Atalan	2013	High	16	26	9	27	1.85	[0.95; 3.60]	100.0%	100.0%
**Antiemetics**										
Bakri–ondan	2015	High	0	32	0	32			--	--
**No control**										
Graham	2006	High	0	4	2	6	0.29	[0.01; 6.00]	100.0%	100.0%

Heterogeneity: $Tau^2 = 0.0069$; $Chi^2 = 19.38$, df = 11 ($P = 0.05$); $I^2 = 43\%$
Test for subgroup differences (common effect): $Chi^2 = 6.50$, df = 4 ($P = 0.16$)
Test for subgroup differences (random effects): $Chi^2 = 4.83$, df = 4 ($P = 0.31$)

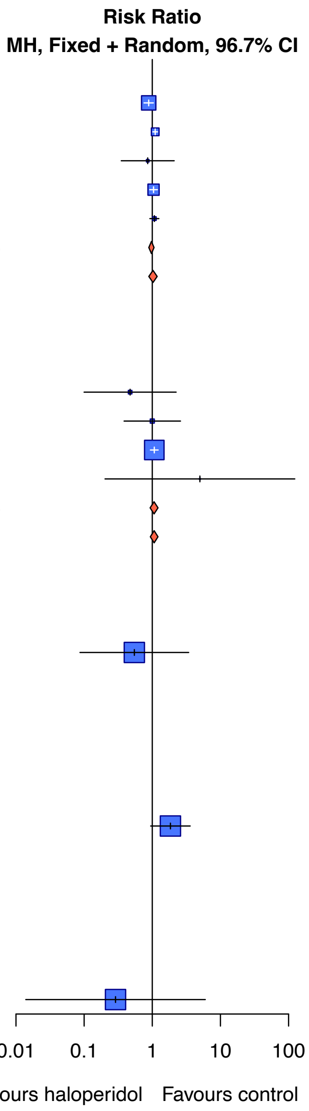

Serious Adverse Events (cummulated) subgrouped by control intervention: worst–best scenario

Figure S18: TSA of cumulated SAEs/SARs for haloperidol versus placebo

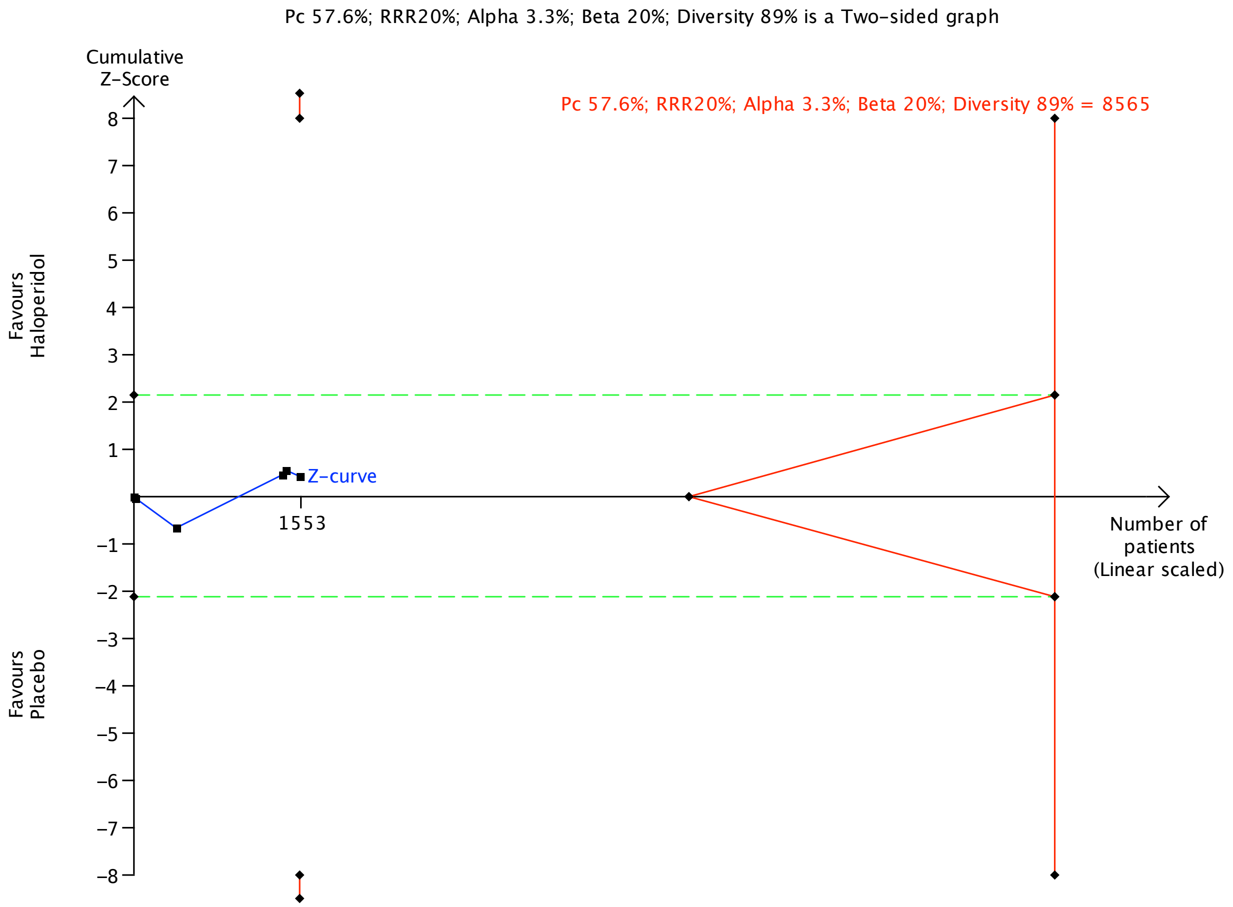

TSA of cumulated SAEs/SARs for trials investigating haloperidol versus placebo. We used a control event rate of 57.6%, a RRR/RRI of 20%, alfa-level of 3.3%, beta 10% (90% power) and diversity of 89% as indicated by the meta-analysis (Figure S14). TSA showed that 18% of the required information size was accrued and we are therefore unable to reject or confirm a RRR/RRI of 20%.

Figure S19: TSA of cumulated SAEs/SARs for haloperidol versus other antipsychotics

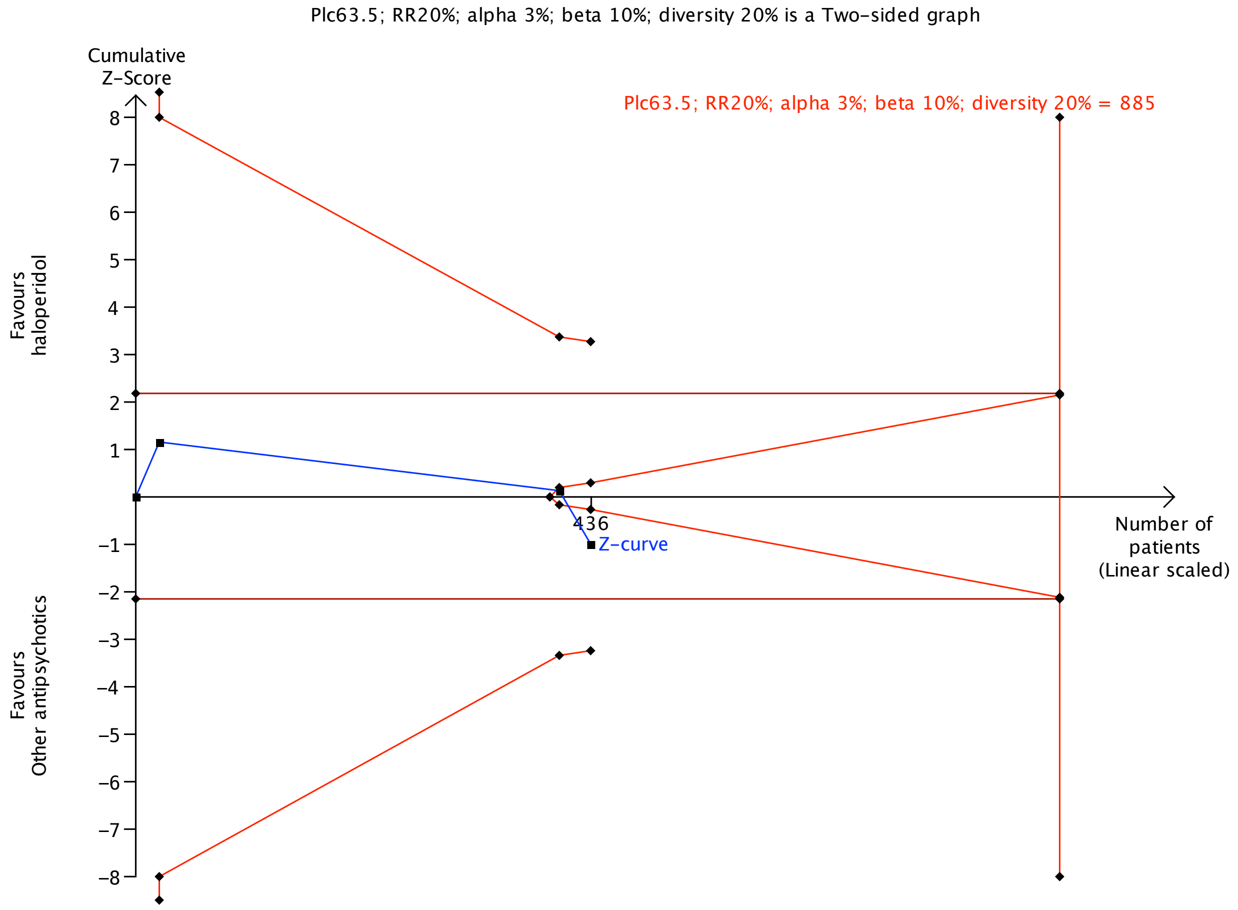

TSA of cumulated SAEs/SARs for trials investigating haloperidol versus other antipsychotics (chlorpromazine, quetiapine, ziprasidone, risperidone). We used a control event rate of 63.5%, a RR of 20%, alfa-level of 3.3%, beta 10% (90% power) and diversity of 20%. TSA showed that 49% of the required information size was accrued and we are therefore unable to reject or confirm a RRR/RRI of 20%.

Figure S20: Days alive without delirium or coma (14 days); haloperidol versus all comparators

Study or Subgroup	Year	RoB	Haloperidol Mean	Haloperidol SD	Haloperidol Total	Control Mean	Control SD	Control Total	MD	98% CI	Weight (fixed)	Weight (random)
Placebo												
Andersen–Ranberg et al. (placebo)	2022	Low	7.10	4.60	481	6.80	4.70	462	0.30	[−0.40; 1.00]	82.8%	82.8%
Garg et al. (placebo)	2022	High	9.40	3.40	15	8.60	2.40	15	0.80	[−1.70; 3.30]	6.6%	6.6%
Girard et al. (placebo)	2018	Low	6.20	8.20	192	5.90	8.20	184	0.30	[−1.67; 2.27]	10.6%	10.6%
**Total (fixed effect, 98% CI)**					**688**			**661**	**0.33**	**[−0.31; 0.97]**	**100.0%**	**--**
**Total (random effect, 98% CI)**									**0.33**	**[−0.31; 0.97]**	**--**	**100.0%**
Heterogeneity: $Tau^2 = 0$; $Chi^2 = 0.2$, df = 2 (P = 0.90); $I^2 = 0\%$												
Other antipsychotics												
Garg et al. (quetiapin)	2022	High	9.40	3.40	15	8.60	2.50	15	0.80	[−1.73; 3.33]	33.1%	33.1%
Girard et al. (ziprasidone)	2018	Low	6.20	8.20	192	6.90	6.70	190	−0.70	[−2.48; 1.08]	66.9%	66.9%
**Total (fixed effect, 98% CI)**					**207**			**205**	**−0.20**	**[−1.66; 1.25]**	**100.0%**	**--**
**Total (random effect, 98% CI)**									**−0.15**	**[−1.83; 1.53]**	**--**	**100.0%**
Heterogeneity: $Tau^2 = 0.2381$; $Chi^2 = 1.27$, df = 1 (P = 0.26); $I^2 = 21\%$												

Heterogeneity: $Tau^2 = 0$; $Chi^2 = 2.09$, df = 4 (P = 0.72); $I^2 = 0\%$
Test for subgroup differences (common effect): $Chi^2 = 0.62$, df = 1 (P = 0.43)
Test for subgroup differences (random effects): $Chi^2 = 0.39$, df = 1 (P = 0.53)

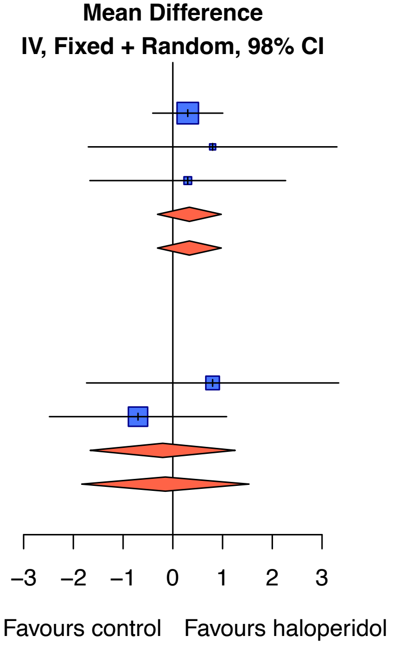

Days Alive without Delirium or Coma (14 days) subgrouped by control intervention

Figure S21: Subgroup analysis of *Days alive without delirium or coma (14 days)* for haloperidol versus placebo; low-risk of bias versus high-risk of bias trials

Study or Subgroup	Year	Haloperidol Mean	SD	Total	Placebo Mean	SD	Total	MD	98% CI	Weight (fixed)	Weight (random)
Low											
Andersen–Ranberg et al. (placebo)	2022	7.10	4.60	481	6.80	4.70	462	0.30	[–0.40; 1.00]	88.6%	88.6%
Girard et al. (placebo)	2018	6.20	8.20	192	5.90	8.20	184	0.30	[–1.67; 2.27]	11.4%	11.4%
**Total (fixed effect, 98% CI)**				**673**			**646**	**0.30**	**[–0.36; 0.96]**	**100.0%**	**––**
**Total (random effect, 98% CI)**								**0.30**	**[–0.36; 0.96]**	**––**	**100.0%**
Heterogeneity: $Tau^2 = 0$; $Chi^2 = 0$, df = 1 (P = 1.00); $I^2 = 0\%$											
High											
Garg et al. (placebo)	2022	9.40	3.40	15	8.60	2.40	15	0.80	[–1.70; 3.30]	100.0%	100.0%

Heterogeneity: $Tau^2 = 0$; $Chi^2 = 0.20$, df = 2 (P = 0.90); $I^2 = 0\%$

Test for subgroup differences (common effect): $Chi^2 = 0.20$, df = 1 (P = 0.65)

Test for subgroup differences (random effects): $Chi^2 = 0.20$, df = 1 (P = 0.65)

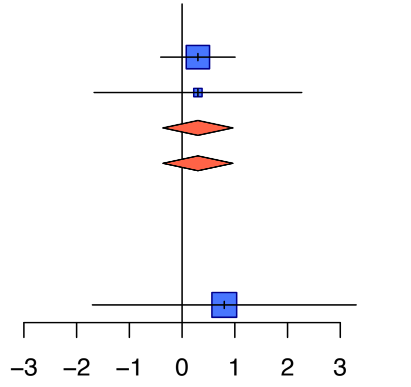

Days Alive without Delirium or Coma (14 days): high versus low risk of bias

Figure S22: Sensitivity analysis best-worst case scenario of *Days alive without delirium or coma*.

Study or Subgroup	Year	RoB	Haloperidol Mean	Haloperidol SD	Control Mean	Control SD	MD	98% CI	Weight (fixed)	Weight (random)
**Placebo**										
Andersen–Ranberg et al.	2022	Low	7.60	5.00	6.30	5.10	1.30	[0.56; 2.04]	81.2%	59.0%
Garg–placebo	2022	High	9.40	3.40	8.60	2.40	0.80	[−1.70; 3.30]	7.2%	16.9%
Girard–placebo	2018	Low	6.20	8.20	5.90	8.20	0.30	[−1.67; 2.27]	11.6%	24.1%
**Total (fixed effect, 98% CI)**							**1.15**	**[0.48; 1.82]**	**100.0%**	**−−**
**Total (random effect, 98% CI)**							**1.15**	**[0.48; 1.82]**	**−−**	**100.0%**
Heterogeneity: $Tau^2 = 0$; $Chi^2 = 1.34$, df = 2 (P = 0.51); $I^2 = 0\%$										
**Other antipsychotics**										
Garg–quetiapin	2022	High	9.40	3.40	8.60	2.50	0.80	[−1.73; 3.33]	33.1%	37.6%
Girard–ziprasidone	2018	Low	6.20	8.20	6.90	6.70	−0.70	[−2.48; 1.08]	66.9%	62.4%
**Total (fixed effect, 98% CI)**							**−0.20**	**[−1.66; 1.25]**	**100.0%**	**−−**
**Total (random effect, 98% CI)**							**−0.15**	**[−1.83; 1.53]**	**−−**	**100.0%**
Heterogeneity: $Tau^2 = 0.2381$; $Chi^2 = 1.27$, df = 1 (P = 0.26); $I^2 = 21\%$										

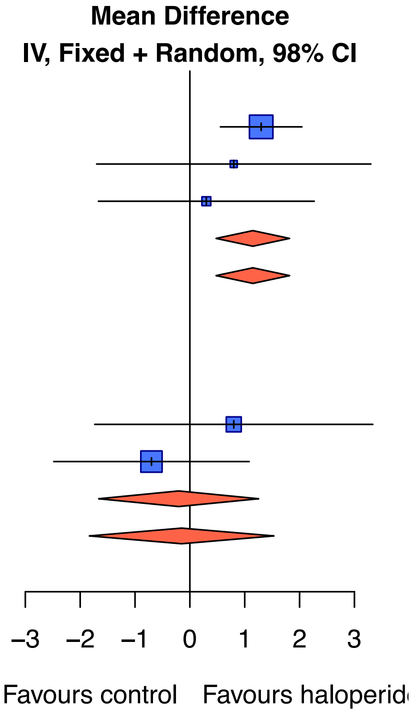

Heterogeneity: $Tau^2 = 0.3222$; $Chi^2 = 6.45$, df = 4 (P = 0.17); $I^2 = 38\%$
Test for subgroup differences (common effect): $Chi^2 = 3.85$, df = 1 (P = 0.05)
Test for subgroup differences (random effects): $Chi^2 = 2.79$, df = 1 (P = 0.10)

Days Alive without Delirium or Coma subgrouped by control intervention: best–worst scenario

Figure S23: Sensitivity analysis worst-best case scenario of *Days alive without delirium or coma*.

Study or Subgroup	Year	RoB	Haloperidol Mean	Haloperidol SD	Control Mean	Control SD	MD	98% CI	Weight (fixed)	Weight (random)
**Placebo**										
Andersen–Ranberg et al.	2022	Low	6.60	5.00	7.30	5.10	−0.70	[−1.44; 0.04]	81.2%	78.5%
Garg–placebo	2022	High	9.40	3.40	8.60	2.40	0.80	[−1.70; 3.30]	7.2%	8.3%
Girard–placebo	2018	Low	6.20	8.20	5.90	8.20	0.30	[−1.67; 2.27]	11.6%	13.2%
**Total (fixed effect, 98% CI)**							**−0.48**	**[−1.15; 0.19]**	**100.0%**	**--**
**Total (random effect, 98% CI)**							**−0.28**	**[−1.29; 0.74]**	**--**	**100.0%**
Heterogeneity: $Tau^2 = 0.1916$; $Chi^2 = 2.74$, df = 2 (P = 0.25); $I^2 = 27\%$										
**Other antipsychotics**										
Garg–quetiapin	2022	High	9.40	3.40	8.60	2.50	0.80	[−1.73; 3.33]	33.1%	33.5%
Girard–ziprasidone	2018	Low	6.20	8.20	6.90	6.70	−0.70	[−2.48; 1.08]	66.9%	66.5%
**Total (fixed effect, 98% CI)**							**−0.20**	**[−1.66; 1.25]**	**100.0%**	**--**
**Total (random effect, 98% CI)**							**−0.15**	**[−1.83; 1.53]**	**--**	**100.0%**
Heterogeneity: $Tau^2 = 0.2381$; $Chi^2 = 1.27$, df = 1 (P = 0.26); $I^2 = 21\%$										

Heterogeneity: $Tau^2 = 0.0220$; $Chi^2 = 4.17$, df = 4 (P = 0.38); $I^2 = 4\%$
Test for subgroup differences (common effect): $Chi^2 = 0.16$, df = 1 (P = 0.69)
Test for subgroup differences (random effects): $Chi^2 = 0.02$, df = 1 (P = 0.88)

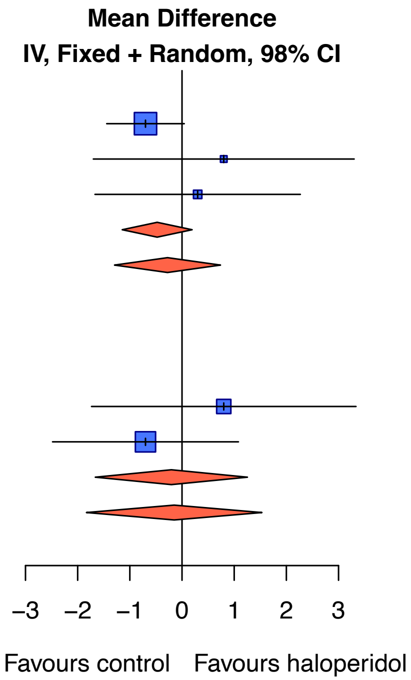

Days Alive without Delirium or Coma subgrouped by control intervention: best–worst scenario

Figure S24: TSA of Days alive without delirium or coma (14 days) for haloperidol versus placebo

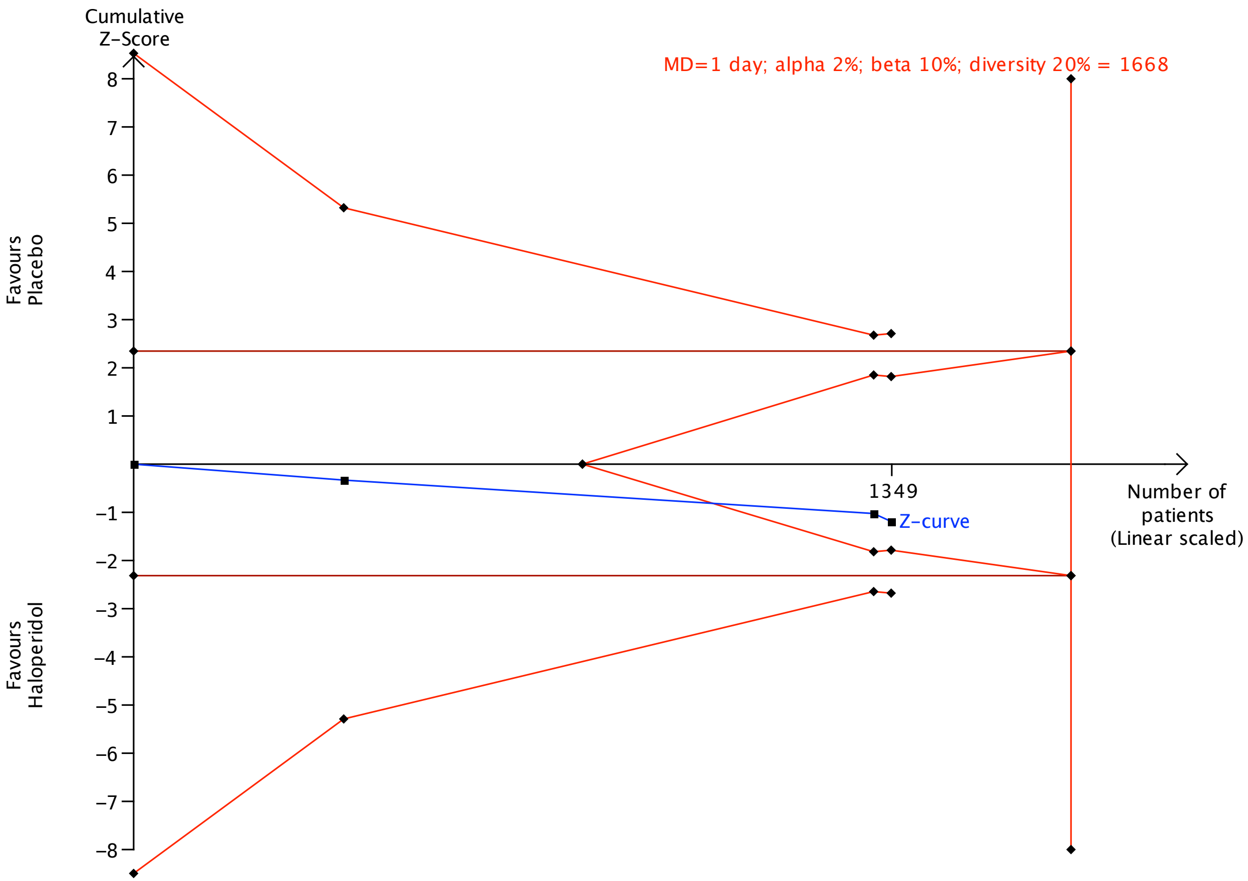

TSA of Days alive without delirium or coma (14 days) for trials investigating haloperidol versus placebo. We used a control MD 1 day, a RR of 20%, alfa-level of 2%, beta 10% (90% power) and diversity of 20%. TSA found that with 80% of the required information size, the cumulated z-curve crossed into the futility area, hence haloperidol does not cause a 20% relative change in days alive without delirium or coma compared with placebo.

Figure S25: TSA sensitivity analysis of Days alive without delirium or coma (14 days) for haloperidol versus placebo (MD=0.33)

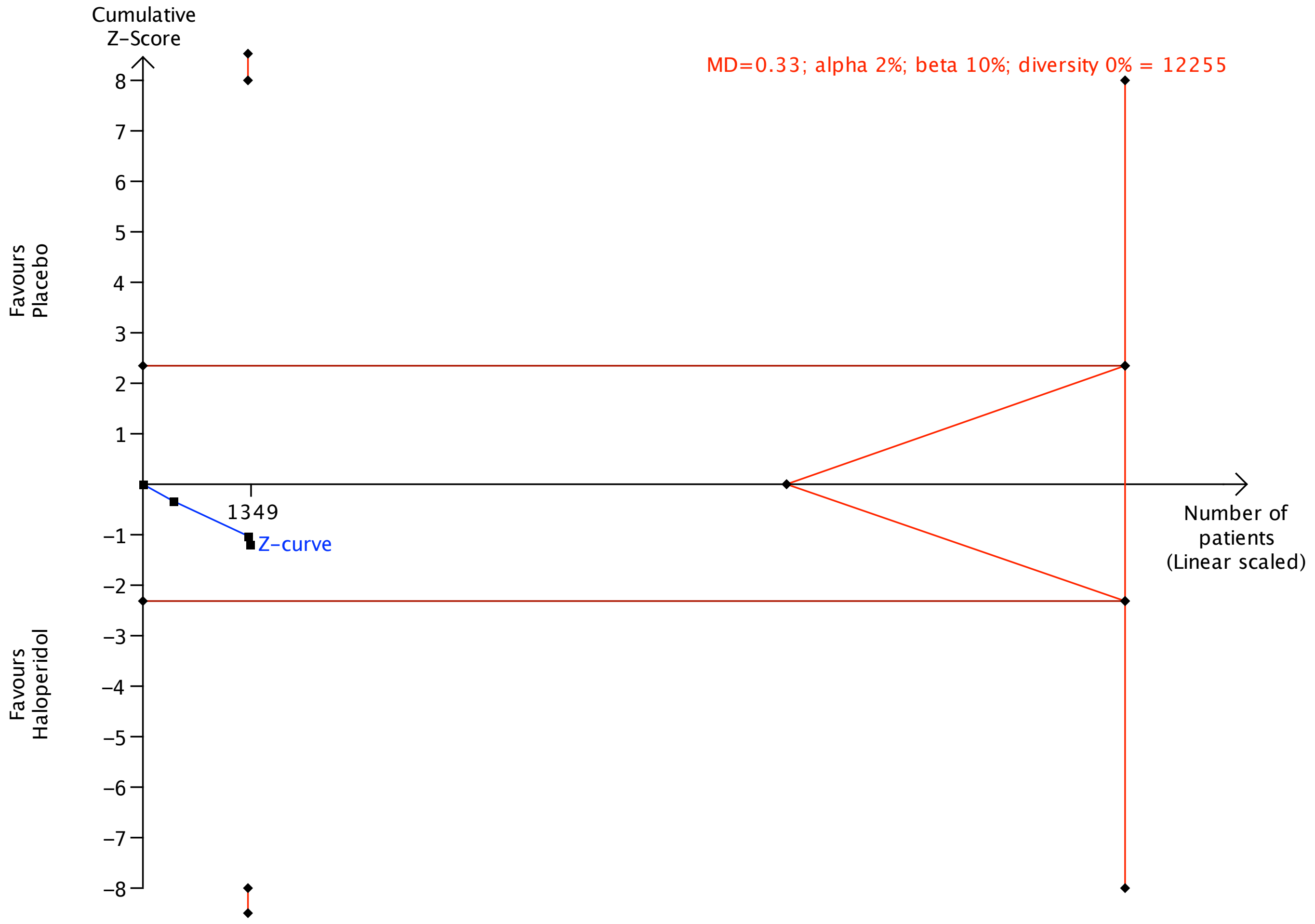

TSA sensitivity analysis of Days alive without delirium or coma (14 days) for trials investigating haloperidol versus placebo. We used an alfa-level of 2%, beta 10% (90% power), control MD 0.33 days and diversity of 0% as indicated by the meta-analysis. TSA showed that 11% of the required information size was accrued and we are therefore unable to reject or accept an absolute mean difference of 0.33 days in days alive without delirium or coma.

Figure S26: TSA of Days alive without delirium or coma (14 days) for haloperidol versus other antipsychotics

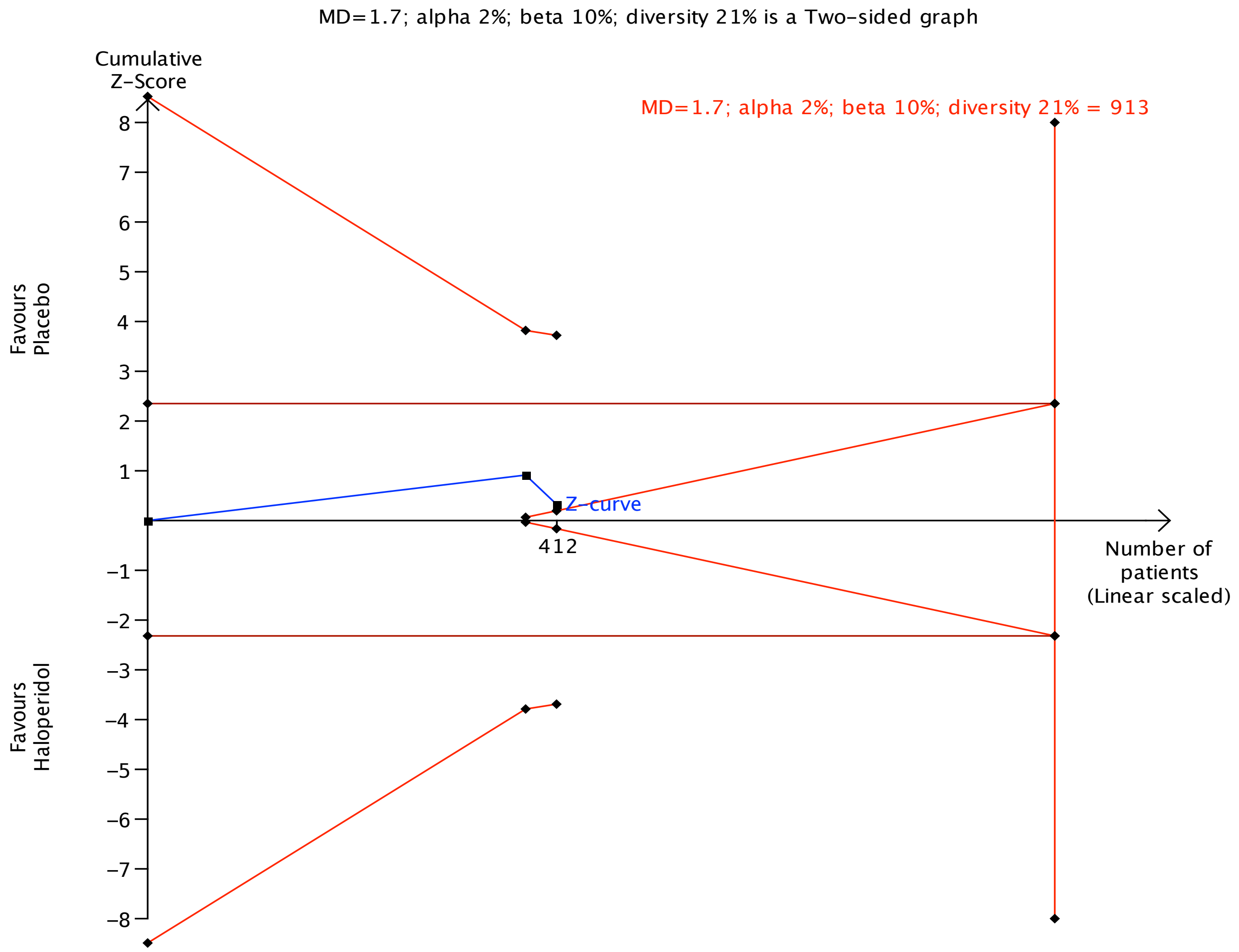

TSA of Days alive without delirium or coma (14 days) for trials investigating haloperidol versus other antipsychotics (ziprasidone and quetiapine). We used a control MD 1.7 days, a RR of 20%, alfa-level of 2%, beta 10% (90% power) and diversity of 21%. TSA showed that 45% of the required information size was accrued and we are therefore unable to reject or accept a RRR/RRI of 20% in days alive without delirium or coma.

Figure S27: Delirium Severity; haloperidol versus all comparators

Study or Subgroup	Year	Severity Scale	Haloperidol Mean	Haloperidol SD	Haloperidol Total	Control Mean	Control SD	Control Total	SMD	98% CI	Weight (fixed)	Weight (random)
**Other antipsychotics**												
Breitbart et al. (chlorpromazine)	1996	DRS	11.60	6.10	11	11.80	6.70	13	−0.03	[−0.98; 0.92]	9.7%	18.5%
Han et al. (risperidone)	2004	MDAS	11.80	9.30	12	16.50	7.30	12	−0.54	[−1.51; 0.43]	9.3%	18.0%
Talebi et al. (quetiapine)	2021	DRS−98	8.50	1.30	100	8.40	1.20	100	0.08	[−0.25; 0.41]	81.0%	63.4%
**Total (fixed effect, 98% CI)**					**123**			**125**	**0.01**	**[−0.29; 0.31]**	**100.0%**	**--**
**Total (random effect, 98% CI)**									**0.01**	**[−0.29; 0.31]**	**--**	**100.0%**
Heterogeneity: $Tau^2 = 0.0004$; $Chi^2 = 2.01$, df = 2 (P = 0.37); $I^2 = 0\%$												
**Benzodiazepines**												
Breitbart et al. (lorazepam)	1996	DRS	11.60	6.10	11	17.00	5.00	6	−0.89	[−2.14; 0.36]	100.0%	100.0%
**Dexmedetomidine**												
Bakri et al. (dexmedetomidine)	2015	ICDSC	3.40	1.10	32	2.90	1.20	32	0.43	[−0.16; 1.02]	100.0%	100.0%
**Antiemetics**												
Bakri et al. (ondansetrone)	2015	ICDSC	3.40	1.10	32	3.50	1.30	32	−0.08	[−0.66; 0.50]	100.0%	100.0%

Heterogeneity: $Tau^2 = 0.0410$; $Chi^2 = 7.66$, df = 5 (P = 0.18); $I^2 = 35\%$
Test for subgroup differences (common effect): $Chi^2 = 5.66$, df = 3 (P = 0.13)
Test for subgroup differences (random effects): $Chi^2 = 5.66$, df = 3 (P = 0.13)

Std. Mean Difference IV, Fixed + Random, 98% CI

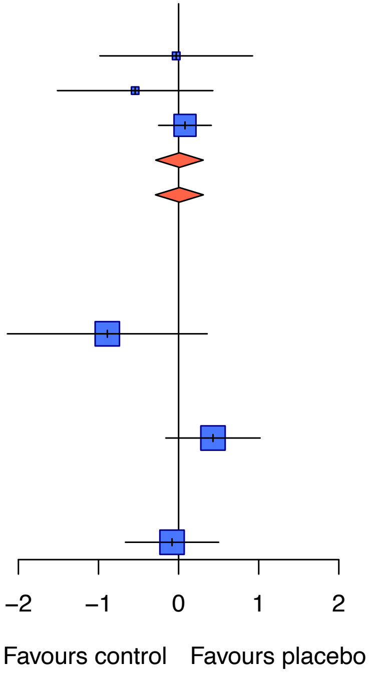

Delirium Severity

Figure S28: Sensitivity analysis best-worst case scenario of Delirium Severity

Study or Subgroup	Year	Severity Scale	Haloperidol Mean	Haloperidol SD	Control Mean	Control SD	SMD	98% CI	Weight (fixed)	Weight (random)
**Other antipsychotics**										
Breitbart – chlorpromazine	1996	DRS	11.60	6.10	11.80	6.70	−0.03	[−0.98; 0.92]	9.5%	14.1%
Han	2004	MDAS	14.50	10.90	16.50	7.30	−0.21	[−1.09; 0.67]	11.1%	16.2%
Talebi	2021	DRS−98	8.50	1.30	8.40	1.20	0.08	[−0.25; 0.41]	79.5%	69.8%
**Total (fixed effect, 98% CI)**							**0.04**	**[−0.26; 0.33]**	**100.0%**	**--**
**Total (random effect, 98% CI)**							**0.04**	**[−0.26; 0.33]**	**--**	**100.0%**
Heterogeneity: $Tau^2 = 0$; $Chi^2 = 0.54$, df = 2 (P = 0.76); $I^2 = 0\%$										
**Benzodiazepines**										
Breitbart – lorazepam	1996	DRS	11.60	6.10	17.00	5.00	−0.89	[−2.14; 0.36]	100.0%	100.0%
**Dexmedetomidine**										
Bakri−dexmed	2015	ICDSC	3.40	1.10	2.90	1.20	0.43	[−0.16; 1.02]	100.0%	100.0%
**Antiemetics**										
Bakri−ondan	2015	ICDSC	3.40	1.10	3.50	1.30	−0.08	[−0.66; 0.50]	100.0%	100.0%

Heterogeneity: $Tau^2 = 0.0173$; $Chi^2 = 6.15$, df = 5 (P = 0.29); $I^2 = 19\%$
Test for subgroup differences (common effect): $Chi^2 = 5.61$, df = 3 (P = 0.13)
Test for subgroup differences (random effects): $Chi^2 = 5.61$, df = 3 (P = 0.13)

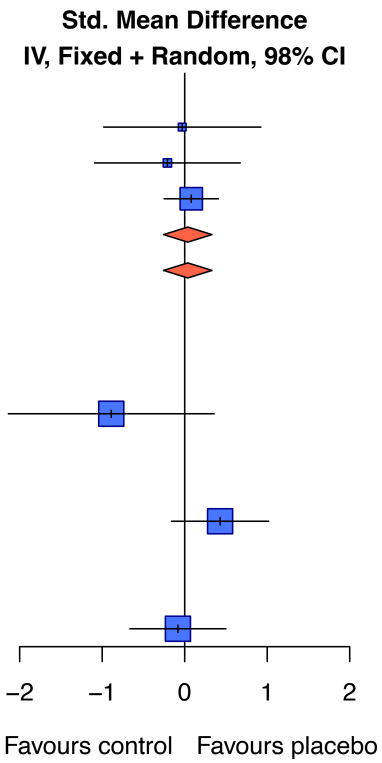

Delirium Severity: best−worst scenario

Figure S29: Sensitivity analysis worst-best case scenario of Delirium Severity

Study or Subgroup	Year	Severity Scale	Haloperidol Mean	Haloperidol SD	Control Mean	Control SD	SMD	98% CI	Weight (fixed)	Weight (random)
**Other antipsychotics**										
Breitbart – chlorpromazine	1996	DRS	11.60	6.10	11.80	6.70	−0.03	[−0.98; 0.92]	9.6%	24.0%
Han	2004	MDAS	9.10	10.90	18.60	7.30	−0.99	[−1.93; −0.05]	9.9%	24.4%
Talebi	2021	DRS−98	8.50	1.30	8.40	1.20	0.08	[−0.25; 0.41]	80.5%	51.6%
**Total (fixed effect, 98% CI)**							**−0.04**	**[−0.33; 0.26]**	**100.0%**	**--**
**Total (random effect, 98% CI)**							**−0.25**	**[−0.99; 0.50]**	**--**	**100.0%**
Heterogeneity: $Tau^2 = 0.2080$; $Chi^2 = 6.29$, df = 2 (P = 0.04); $I^2 = 68\%$										
**Benzodiazepines**										
Breitbart – lorazepam	1996	DRS	11.60	6.10	17.00	5.00	−0.89	[−2.14; 0.36]	100.0%	100.0%
**Dexmedetomidine**										
Bakri−dexmed	2015	ICDSC	3.40	1.10	2.90	1.20	0.43	[−0.16; 1.02]	100.0%	100.0%
**Antiemetics**										
Bakri−ondan	2015	ICDSC	3.40	1.10	3.50	1.30	−0.08	[−0.66; 0.50]	100.0%	100.0%

Heterogeneity: $Tau^2 = 0.1088$; $Chi^2 = 12.12$, df = 5 (P = 0.03); $I^2 = 59\%$
Test for subgroup differences (common effect): $Chi^2 = 5.82$, df = 3 (P = 0.12)
Test for subgroup differences (random effects): $Chi^2 = 6.34$, df = 3 (P = 0.10)

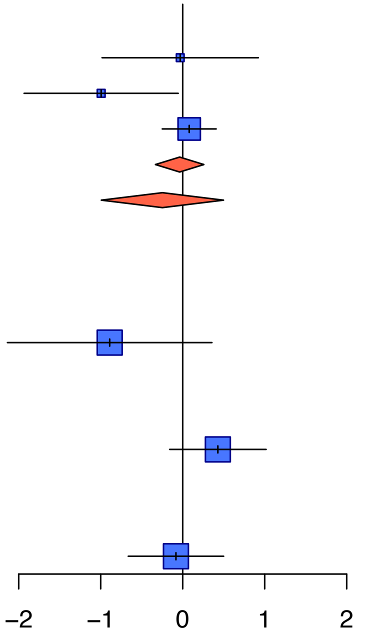

Delirium Severity: worst−best scenario

Figure S30: Cognitive function; haloperidol versus all comparators

Study or Subgroup	Year	Risk of Bias	Haloperidol Mean	Haloperidol SD	Haloperidol Total	Control Mean	Control SD	Control Total	MD	98% CI	Weight (fixed)	Weight (random)
**Other antipsychotics**												
Breitbart et al. (chlorpromazine)	1996	High	17.20	12.10	11	15.10	10.40	13	2.10	[−8.72; 12.92]	100.0%	100.0%
**Benzodiazepines**												
Breitbart et al. (lorazepam)	1996	High	17.20	12.10	11	11.50	8.70	6	5.70	[−6.14; 17.54]	100.0%	100.0%
**No control**												
Graham et al. (no control)	2006	High	22.50	12.10	4	18.50	10.40	6	4.00	[−13.19; 21.19]	100.0%	100.0%

Heterogeneity: $Tau^2 = 0$; $Chi^2 = 0.27$, df = 2 (P = 0.87); $I^2 = 0\%$
Test for subgroup differences (common effect): $Chi^2 = 0.27$, df = 2 (P = 0.87)
Test for subgroup differences (random effects): $Chi^2 = 0.27$, df = 2 (P = 0.87)

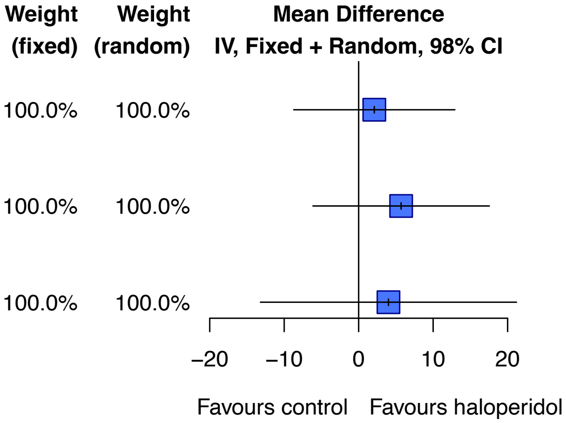

Cognitive function. All studies used MMSE end–scores. Follow–up varied from 6–9 days

Figure S31: QTc prolongation; haloperidol versus all comparators

Study or Subgroup	Year	Risk of Bias	Haloperidol Events	Haloperidol Total	Control Events	Control Total	RR	95% CI	Weight (fixed)	Weight (random)
**Placebo**										
Andersen–Ranberg et al. (placebo)	2022	Low	12	501	7	486	1.66	[0.66; 4.19]	38.6%	40.3%
Girard et al. (placebo)	2018	Low	13	192	10	184	1.25	[0.56; 2.77]	55.4%	50.9%
ORIC–1 (placebo)	2017	High	3	16	1	13	2.44	[0.29; 20.75]	6.0%	8.8%
**Total (fixed effect, 95% CI)**			**28**	**709**	**18**	**683**	**1.48**	**[0.83; 2.64]**	**100.0%**	**--**
**Total (random effect, 95% CI)**							**1.47**	**[0.82; 2.63]**	**--**	**100.0%**
Heterogeneity: $Tau^2 = 0$; $Chi^2 = 0.45$, df = 2 (P = 0.80); $I^2 = 0\%$										
**Dexmedetomidine**										
Bakri et al. (dexmedetomidine)	2015	High	0	32	0	32			--	--
**Antiemetics**										
Bakri et al. (ondansetrone)	2015	High	0	32	0	32			--	--
**Other antipsychotics**										
Girard et al. (ziprasidone)	2018	Low	13	192	20	190	0.64	[0.33; 1.26]	100.0%	100.0%
**No control**										
Graham et al. (no control)	2006	High	1	4	0	6	4.33	[0.22; 84.16]	100.0%	100.0%

Heterogeneity: $Tau^2 = 0.0475$; $Chi^2 = 4.66$, df = 4 (P = 0.32); $I^2 = 14\%$
Test for subgroup differences (common effect): $Chi^2 = 4.22$, df = 2 (P = 0.12)
Test for subgroup differences (random effects): $Chi^2 = 4.21$, df = 2 (P = 0.12)

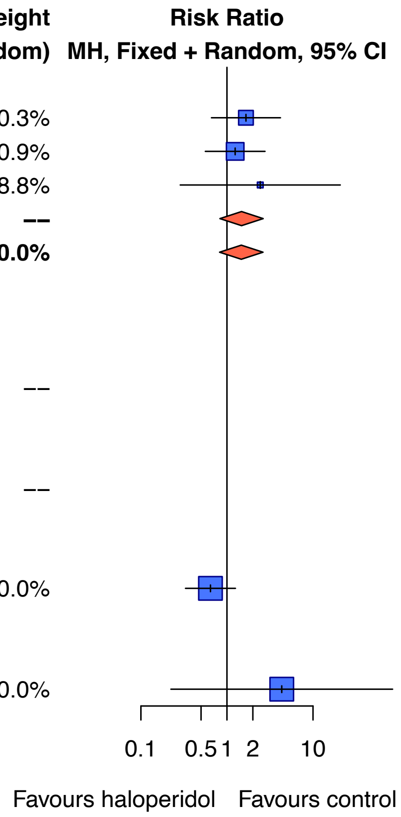

Figure S32: Subgroup analysis of QTc prolongation for haloperidol versus placebo; low-risk of bias versus high-risk of bias trials

Study or Subgroup	Year	Haloperidol Events	Haloperidol Total	Placebo Events	Placebo Total	RR	95% CI	Weight (fixed)	Weight (random)
**Risk of Bias = Low**									
Andersen–Ranberg et al. (placebo)	2022	12	501	7	486	1.66	[0.66; 4.19]	41.0%	42.8%
Girard et al. (placebo)	2018	13	192	10	184	1.25	[0.56; 2.77]	59.0%	57.2%
**Total (fixed effect, 95% CI)**		**25**	**693**	**17**	**670**	**1.42**	**[0.78; 2.59]**	**100.0%**	**--**
**Total (random effect, 95% CI)**						**1.41**	**[0.77; 2.58]**	**--**	**100.0%**
Heterogeneity: $Tau^2 = 0$; $Chi^2 = 0.21$, df = 1 (P = 0.64); $I^2 = 0\%$									
**Risk of Bias = High**									
ORIC–1 (placebo)	2017	3	16	1	13	2.44	[0.29; 20.75]	100.0%	100.0%

Heterogeneity: $Tau^2 = 0$; $Chi^2 = 0.45$, df = 2 (P = 0.80); $I^2 = 0\%$
Test for subgroup differences (common effect): $Chi^2 = 0.23$, df = 1 (P = 0.63)
Test for subgroup differences (random effects): $Chi^2 = 0.23$, df = 1 (P = 0.63)

Risk Ratio MH, Fixed + Random, 95% CI

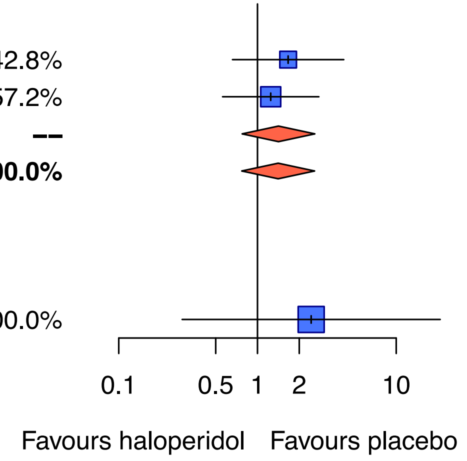

QTc prolongation. High risk versus low risk of bias

Figure S33: Sensitivity analysis best-worst case scenario of QTc prolongation

Study or Subgroup	Year	Risk of Bias	Haloperidol Events	Haloperidol Total	Control Events	Control Total	RR	98% CI	Weight (fixed)	Weight (random)
**Placebo**										
Andersen–Ranberg et al.	2022	Low	12	510	11	490	1.05	[0.40; 2.74]	47.6%	44.1%
Girard–placebo	2018	Low	13	192	10	184	1.25	[0.48; 3.22]	43.3%	45.2%
ORIC–1	2017	High	3	16	2	14	1.31	[0.19; 9.18]	9.1%	10.7%
**Total (fixed effect, 98% CI)**			**28**	**718**	**23**	**688**	**1.16**	**[0.61; 2.19]**	**100.0%**	**--**
**Total (random effect, 98% CI)**							**1.16**	**[0.61; 2.20]**	**--**	**100.0%**
Heterogeneity: $Tau^2 = 0$; $Chi^2 = 0.11$, df = 2 (P = 0.95); $I^2 = 0\%$										
**Other antipsychotics**										
Girard–ziprasidone	2018	Low	13	192	20	190	0.64	[0.29; 1.42]	100.0%	100.0%
**Dexmedetomidine**										
Bakri–dexmed	2015	High	0	32	0	32			--	--
**Antiemetics**										
Bakri–ondan	2015	High	0	32	0	32			--	--
**No control**										
Graham	2006	High	1	4	0	6	4.33	[0.13; 146.53]	100.0%	100.0%

Heterogeneity: $Tau^2 = 0$; $Chi^2 = 2.96$, df = 4 (P = 0.56); $I^2 = 0\%$
Test for subgroup differences (common effect): $Chi^2 = 2.78$, df = 2 (P = 0.25)
Test for subgroup differences (random effects): $Chi^2 = 2.85$, df = 2 (P = 0.24)

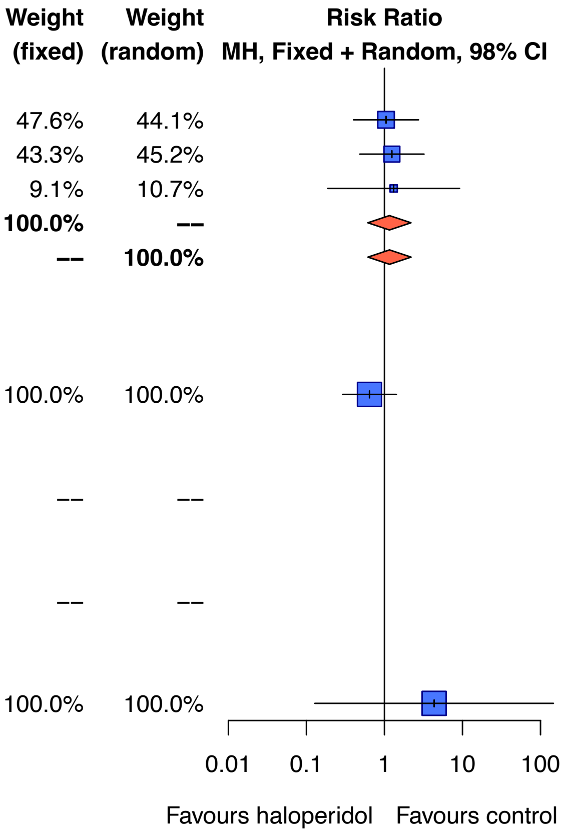

QTc prolongation: best–worst scenario

Figure S34: Sensitivity analysis worst-best case scenario of QTc prolongation

Study or Subgroup	Year	Risk of Bias	Haloperidol Events	Haloperidol Total	Control Events	Control Total	RR	95% CI	Weight (fixed)	Weight (random)
**Placebo**										
Andersen–Ranberg et al.	2022	Low	21	510	7	490	2.88	[1.24; 6.72]	38.8%	42.1%
Girard–placebo	2018	Low	13	192	10	184	1.25	[0.56; 2.77]	55.4%	43.8%
ORIC–1	2017	High	3	16	1	14	2.62	[0.31; 22.46]	5.8%	14.1%
**Total (fixed effect, 95% CI)**			**37**	**718**	**18**	**688**	**1.96**	**[1.13; 3.40]**	**100.0%**	**--**
**Total (random effect, 95% CI)**							**1.90**	**[1.06; 3.41]**	**--**	**100.0%**
Heterogeneity: $Tau^2 = 0.0152$; $Chi^2 = 2.1$, df = 2 (P = 0.35); $I^2 = 5\%$										
**Dexmedetomidine**										
Bakri–dexmed	2015	High	0	32	0	32			--	--
**Antiemetics**										
Bakri–ondan	2015	High	0	32	0	32			--	--
**Other antipsychotics**										
Girard–ziprasidone	2018	Low	13	192	20	190	0.64	[0.33; 1.26]	100.0%	100.0%
**No control**										
Graham	2006	High	1	4	0	6	4.33	[0.22; 84.16]	100.0%	100.0%

Heterogeneity: $Tau^2 = 0.3214$; $Chi^2 = 8.71$, df = 4 (P = 0.07); $I^2 = 54\%$
Test for subgroup differences (common effect): $Chi^2 = 6.97$, df = 2 (P = 0.03)
Test for subgroup differences (random effects): $Chi^2 = 6.44$, df = 2 (P = 0.04)

Risk Ratio MH, Fixed + Random, 95% CI

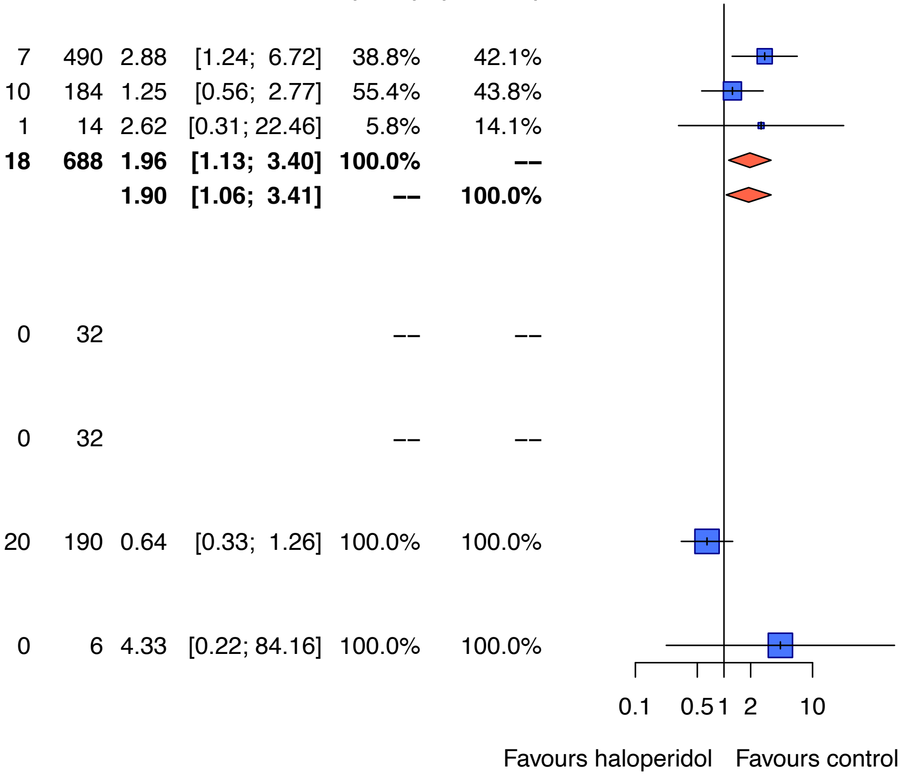

QTc prolongation: worst–best scenario

Definition of Serious Adverse Events and Serious Adverse Reactions

We defined SAEs and SARs according to the International Conference on Harmonisation Good Clinical Practice (ICH-GCP) and as reported in each trial. Consequently, an SAE was defined as any reported adverse event that resulted in death, was life-threatening, required hospitalisation or prolongation of existing hospitalisation, resulted in persistent or significant disability or incapacity. A SAR was defined as any reported adverse event related to haloperidol (according to the Summary of Product Characteristics of haloperidol) that resulted in death, was life-threatening, required hospitalisation or prolongation of existing hospitalisation, resulted in persistent or significant disability or incapacity.

Two methods were used to analyse SAEs/SARs: (1) highest proportion of reported SAEs/SARs which was the most frequently reported SAE/SAR in each group, this estimate assumes that patients who experienced other SAEs/SARs had already experienced the most frequently reported SAE/SAR; and (2) calculating the cumulative number of SAEs/SARs in each group which is the sum of all reported SAEs/SARs, this estimate assumes that each patient will only experience one SAE/SAR. We expected the actual number of patients experiencing one or more SAEs/SARs to fall between these two measures.

Table S2. Individual Serious Adverse Events and Serous Adverse Reactions reported in included trials

Placebo-controlled trials

Serious Adverse Event or Serious Adverse Reaction	Number of trials reporting the event	Events in haloperidol group	Events in placebo group
Fell or stepped out of bed	1	6	18
ARDS	1	0	3
Sepsis	1	0	3
Auto-removal of invasive devices	1	5	10
Renal failure	1	0	2
Tracheostomy	1	0	2
Pancytopenia	1	0	2
Removal of urinary catheter	1	5	9
Muscle rigidity and associated movement disorders	1	3	1
Failure to wean from ventilator	1	2	0
Pseudomonas pneumonia	1	2	0
Ventricular arrhythmia	2	9	5
Abdominal surgery closure	1	0	1
Bacterial peritonitis	1	0	1
Clostridium difficile colitis	1	0	1
DVT	1	0	1
Encephalopathy	1	0	1
Lower GI bleed	1	0	1
Right Upper Lung collapse	1	0	1
Sacral decubitus	1	0	1
VATS/Empyema	1	0	1
Mortality	5	272	295

Oversedation	1	42	46
Neuroleptic malignant syndrome	1	0	1
Extrapyramidal symptoms	2	4	2
Anaphylactic reaction	1	1	0
Tardive dyskinesia	1	1	0
Dystonia	1	1	0
Atrial fibrillation	1	1	0
Desaturation	1	1	0
Extremity ischemia	1	1	0
Gastric and duodenal ulcers	1	1	0
GU haemorrhage related to autoremoval of Foley catheter	1	1	0
Intrapulmonary haemorrhage	1	1	0
IVC	1	1	0
Lower lobectomy	1	1	0
OR cleansing and closure of leg wound	1	1	0
Polymicrobial bilateral pneumonia	1	1	0
Reintubation	1	1	0
Ventricular tachycardia	1	1	0
Torsades de Pointes	1	2	1
ICU readmission	1	27	23
Physical restraint	1	48	48
Pneumonia	1	2	2
Acute renal failure	1	1	1
Hepatic encephalopathy	1	1	1
Nosocomial pneumonia	1	1	1
Self-extubation	2	2	2
Acute hepatic failure	1	1	1

Agranulocytosis	0	0	0

Trials with other comparators (other antipsychotics, dexmedetomidine, benzodiazepines, morphine, antiemetics and no intervention).

Serious Adverse Event or Serious Adverse Reaction	Number trials reporting the event	Events in haloperidol group	Events in control group
Re-intubation	1	6	1
ICU re-admission	2	35	25
Oversedation	1	42	50
Extrapyramidal symptoms	2	7	1
Dystonia	1	1	0
Torsades de Pointes	1	2	1
Mortality	6	85	81
Self-extubation	1	1	1
Dystonia	1	0	0
